# Hawaiian picture‐winged *Drosophila* exhibit adaptive population divergence along a narrow climatic gradient on Hawaii Island

**DOI:** 10.1002/ece3.4844

**Published:** 2019-02-18

**Authors:** Jon Eldon, M. Renee Bellinger, Donald K. Price

**Affiliations:** ^1^ Tropical Conservation Biology and Environmental Science University of Hawaii Hilo Hawaii; ^2^ University of Hawaii Hilo Hawaii; ^3^ Present address: Indiana University Bloomington Indiana; ^4^ Present address: University of Nevada – Las Vegas Las Vegas Nevada

**Keywords:** climate change, gene expression, Hawaiian *Drosophila*, local adaptation, population divergence

## Abstract

1. Anthropogenic influences on global processes and climatic conditions are increasingly affecting ecosystems throughout the world.
2. Hawaii Island’s native ecosystems are well studied and local long‐term climatic trends well documented, making these ecosystems ideal for evaluating how native taxa may respond to a warming environment.
3. This study documents adaptive divergence of populations of a Hawaiian picture‐winged *Drosophila*, *D. sproati,* that are separated by only 7 km and 365 m in elevation.
4. Representative laboratory populations show divergent behavioral and physiological responses to an experimental low‐intensity increase in ambient temperature during maturation. The significant interaction of source population by temperature treatment for behavioral and physiological measurements indicates differential adaptation to temperature for the two populations.
5. Significant differences in gene expression among males were mostly explained by the source population, with eleven genes in males also showing a significant interaction of source population by temperature treatment.
6. The combined behavior, physiology, and gene expression differences between populations illustrate the potential for local adaptation to occur over a fine spatial scale and exemplify nuanced response to climate change.

## 1 INTRODUCTION

Global climatic changes ascribed to anthropogenic activities are causing increasing and likely irreversible changes to ecological systems, including those that have largely escaped the impacts of land conversion, invasive species, and other more direct forms of human disturbances (Barnosky et al., 2012; Lister & Garcia, 2018). Understanding how native species and ecosystems are responding to these changes is critical for mitigating the effects of changes in both natural and managed systems (Birkett, Blackburn, & Menéndez, 2018, Dahlhoff & Rank, 2000, Hoffmann & Sgrò 2011). Accordingly, anticipating these ecological responses is an urgent area of research for ecologists, with implications that extend beyond conservation into agriculture, ecosystem services, and other diverse topics (Cardinale et al., 2012).

The Hawaiian archipelago is a classic model system for the study of ecological and evolutionary processes, particularly those related to speciation, biogeography, and ecosystem change (Carson & Clague, 1995; Vitousek, 2004). The high islands of this volcanic archipelago contain steep environmental gradients over short geographical distances that result in highly heterogeneous landscapes (Wilson, 1963). This natural combination of isolating forces, diverse habitat, and variable biotic communities, which together encourage population differentiation and local adaptation, is thought to account for the large number of adaptive radiations and endemic species found within the archipelago (Gillespie & Roderick, 2002; Price & Clague 2002). These native species and ecosystems may be particularly vulnerable to global climate change due to the relatively small natural habitat patches and population sizes, negative impacts from invasive species, and dramatic habitat degradation, fragmentation, and loss (Hobbelen, Samuel, Foote, Tango, & LaPointe, 2013; Uy, LeDuc, Ganote, & Price, 2015). As evidence of this, numerous recent extinctions have occurred, and many of the remaining native species are increasingly confined to small preserves or found only at higher elevations (Benning, LaPointe, Atkinson, & Vitousek, 2002; Howarth & Gagné, 2012).

This archipelago is also closely associated with long‐term measurement of global atmospheric changes and the identification of the role of anthropogenic activities as a driving force (Benning et al., 2002; Manning, Nisbet, Keeling, & Liss, 2011). The 50 + year continuous monitoring of atmospheric CO_2_ levels on the Mauna Loa volcano of Hawaii's Big Island documents a historically unprecedented increase, known as the “Keeling Curve,” which is now referenced throughout the world as evidence of human‐induced climate change (Giambelluca, Diaz, & Luke, 2008; Keeling, Whorf, Wahlen, & Vanderplicht, 1995). The environmental implications of atmospheric accumulation of greenhouse gasses are well documented both globally and in Hawaii, and the latter has experienced a steady increase in air temperature and a 15% decrease in rainfall over the last few decades (Chu & Chen, 2005; Giambelluca et al., 2008). Atmospheric models project a further 1.4–5.8°C increase in global temperature by 2,100, with an accelerated increase at higher elevations and increasingly volatile weather patterns as a result (Lemke et al., 2007).

The Hawaiian *Drosophila* are a well‐studied part of these island ecosystems and have been used as indicators of biogeographic history, habitat disturbances, and other environmental changes (Eldon, Price, Magnacca, & Price, 2013; Price & Muir, 2008). Most of the approximately 800 species in this group appear to have radiated from a single colonization event approximately 25 million years ago, and 120 of these species belong to the large, charismatic, and well‐studied picture‐winged group (Katoh, Izumitani, Yamashita, & Watada, 2016; O'Grady et al., 2011). Twelve picture‐winged *Drosophila* species are currently listed as endangered or threatened, and monitoring over the last 30 years has documented sharp declines and reduced distributions for many of the nonlisted species (Richardson, 2006). These *Drosophila*, like many tropical ectotherms, are thought to be particularly vulnerable to climatic changes due to their narrow physiological tolerance windows (Saxon, O'Brien, & Bridle, 2018), as well as limited habitat ranges and highly specific native host plant associations (Magnacca, Foote, & O'Grady, 2008; Magnacca & Price, 2015).

Understanding how species respond to climate change is a pertinent theoretical question and an immediate conservation priority (Hoffmann & Sgrò 2011; Kellermann et al., 2009; Porcelli, Gaston, Butlin, & Snook, 2017). A recent study of two Hawaii Island endemic picture‐winged *Drosophila*, the rare *D. silvestris* and the more ubiquitous *D. sproati*, found strong species‐level differences in temperature tolerance (Uy et al., 2015), and previously observed clinal patterns of genetic differentiation in *D. silvestris* (Craddock & Carson 1989) suggest that temperature may also be driving adaptive population divergence. This current study continues such investigations of *D. sproati* by testing for adaptive divergence between the highest and lowest elevation populations within a fragmented portion of wet forest habitat on the east side of Hawaii Island. Wild‐caught individuals from each site were assessed for genetic differentiation at putative neutral loci and used to found representative laboratory populations that were tested for differences in behavior, physiology, and gene expression, following a nonfatal temperature increase during maturation. This experimental design mimics the predicted increase in ambient temperatures in association with climate change (Giambelluca et al., 2008), and the mixed‐methods approach has the potential to offer a comprehensive picture of adaptive population divergence (Flatt, 2016; Hoffmann, Sørensen, & Loeschcke, 2003).

## 2 MATERIALS AND METHODS

### 2.1 Site description

This study focuses on populations of *D. sproati* in the convergence zone of the Mauna Loa and Mauna Kea volcanoes on the east side of Hawaii Island. A series of lava flows in this region ending in the 1800 s created islands of montane wet forest habitat, known as “kipuka,” surrounded by young substrate that supports only pioneer species. *D. sproati, *a relatively common picture‐winged *Drosophila* endemic to montane wet forests around the island, can only be found in this region within kipuka between the elevations of 1,260 m and 1625 m (J. Eldon, pers. obs.). While less mobile species, such as the endemic *Tetragnatha* spiders, have been found to display neutral population differentiation among these kipukas, neutral differentiation among more mobile species has not been well studied in this habitat mosaic and only rarely at this scale (Vandergast, Gillespie, & Roderick, 2004), and potential for adaptive divergence is an underexplored area of investigation. Accordingly, this study compares *D. sproati* from the lowest elevation kipuka (“Low” at 1,260 m) where the species can be found in this region against those from the highest (“High” at 1625 m), which is approximately 7 km upslope. The suitability of the two sites as representing mature wet forest habitat was quantified through a surface cover and tree/tree fern surveys conducted at the center and edge of each kipuka. The presence of a climatic gradient corresponding to elevation was assessed through concurrent measurement of temperature and humidity during a 2‐week period using HOBO Pro v2 data loggers placed under shaded covers at the center and edge of each kipuka.

### 2.2 Collection and maintenance of fly stocks

A total of 35 and 32 adult *D. sproati* were captured from the center of each kipuka using fermented banana bait. The flies were immediately transferred to one‐gallon breeding jars kept in a climate‐controlled laboratory and maintained at 18°C, the standard rearing temperature for picture‐winged *Drosophila* (Uy et al., 2015, Table 1). Each jar contained a layer of sand to regulate moisture and vials containing a standard Hawaiian *Drosophila* agar food medium with a tissue soaked in pulverized rotten bark from *Cheirodendron trigynum,* the larval host plant for this species (Droney, 1992; Magnacca et al., 2008; Price & Boake, 1995). These vials were replaced every 3–4 days, and those that contained larvae were transferred to 1‐gallon pupation jars that contained a layer of slightly larger sand. Adults that emerged within these jars were transferred to new breeding jars. These laboratory populations were maintained in this controlled environment at a concentration of approximately 250 interbreeding adults for five generations. The wild‐caught founders were placed in 95% EtOH within 12 hr of dying and stored at −20°C for subsequent DNA extraction and analysis.

**Table 1:** Number of flies or mating groups sampled for each analysis. Wild‐caught flies were used to found the laboratory populations and were sequenced at the COII and YP1 gene regions. The number of analyzed sequences is given in Table S2. All other flies came from the sixth generation of the representative laboratory populations and were assessed during their fourth week of maturity

Analysis	Sex	Low elevation		High elevation	
		18°C	24°C	18°C	24°C
Wild‐caught	# Male	13		12	
	# Female	22		20	
Physiology—Heat knockdown	# Male	67	35	28	12
	# Female	60	53	28	30
Physiology—Chill coma recovery	# Male	33	32	19	13
	# Female	40	51	30	36
Behavior	# Groups	22	24	16	14
Gene expression	# Male	7	7	5	5
	# Female	6	5	6	3

### 2.3 Experimental temperature treatment

**Figure 1:**
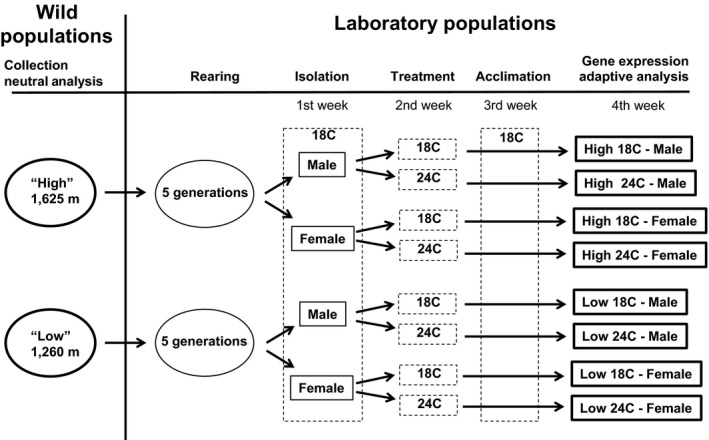
A schematic of the experimental design, showing the *Drosophila sproati* populations, treatments, and analyses. “Neutral analysis” consisted of sequence analysis of putatively neutral regions within the COII and YP1 genes. “Adaptive analysis” consisted of measures of heat knockdown resistance and cold chill coma recovery, activity levels and courtship displays, and microarray analysis of differential gene expression

Flies from the sixth laboratory generation were isolated by sex within 24 hr of emergence. Half of each population/sex group were maintained at 18°C for the first week of maturation, moved to a 24°C climate‐controlled laboratory for the second week, and returned to 18°C for the third week, while the other half matured at a constant 18°C (Figure 1). Initial pilot studies had found that one week at 24°C did not result in heat‐induced sterilization or fatality for *D. sproati *(J. Eldon, unpublished data). Subsequent analyses were performed on adult flies between 3 and 4 weeks old from each population/sex/temperature group, with different flies used for each physiological and behavioral analysis.

### 2.4 Behavioral analysis

Behavioral analysis focused on overall activity levels and male social displays, which are similar to the closely related *D. grimshawi* and described in Table S1 (Ringo & Hodosh, 1978). These measures are common assessments of species and population divergence in *Drosophila,* and thermal stress has been found to inhibit courtship behavior in some species (Patton & Krebs, 2001). *Drosophila sproati* is thought to form leks or collapsed leks during mating; thus, behavioral analysis was conducted on groups consisting of three virgin male and three virgin female flies (Droney, 1992). The numbers and types of independent male displays were recorded during six one‐minute observational periods at 15‐ to 20‐min intervals between the hours of 9:30 a.m. and 12:30 p.m. The number of stationary flies per group was recorded during each observational period as a measure of overall activity level.

### 2.5 Physiological analysis

The physiological analysis measured the response of each population/sex/temperature group to subsequent high‐intensity heat and cold shocks. Heat knockdown resistance was measured by exposing flies to 32.5°C and recording the time until knockdown at half hour intervals. Cold chill coma recovery was measured by chilling flies at 2°C for 1.5 hr to induce a comatose state, then returning them to 18°C and recording the number of minutes until each fly righted themselves. These two tests are conventional measures of thermal tolerance in *Drosophila* and have been shown to be heritable and ecologically relevant traits (Hoffmann, Anderson, & Hallas, 2002; Norry, Scannapieco, Sambucetti, Bertoli, & Loeschcke, 2008). An initial pilot study was performed to determine appropriate knockdown and chill coma temperatures for *D. sproati* (J. Eldon, unpublished data).

### 2.6 Data analysis and statistics

A two‐way analysis of variance (ANOVA) was used to assess the significance of source population, temperature treatment, and the interaction of the two factors on the behavioral and physiological measures, using the statistical program MiniTab version 15, with all data first tested for normality. For response variables that exhibited significant block effects due to differences among the weeks of the study, the residuals from an analysis of variance for the block effect were used in subsequent analysis of source population and treatment effects. The ANOVA analyses were performed on all response variables (or the residuals) in a two‐way interaction model with source population and temperature treatment as the main factors considered random variables (i.e., main effects mean squares were tested with the interaction mean square as the error term). Pairwise significance testing was performed among all population/sex/temperature groups for each measure using Tukey's multiple comparison tests and a significance threshold of *p* < 0.05.

### 2.7 DNA extraction and analysis of neutral loci

DNA was extracted from all wild‐caught flies using a nondestructive soaking technique specially developed for arthropods (Rowley et al., 2007) and subsequently purified with a Qiagen DNeasy kit (Qiagen, Hilden, Germany) and stored at −20°C. Between‐population genetic differentiation was measured using the mitochondrial cytochrome oxidase II gene (COII) and nuclear yolk protein I gene (YPI) as described in Eldon et al., 2013. These two genes showed no significant differentiation within *D. sproati* sampled among multiple locations within the large eastern wet forest region of Hawaii Island that encompasses the study site (Eldon et al., 2013, unpublished data). Sequencher version 4.9 was used to visually aligned and edit sequences and Arlequin version 3.1 was used to perform all statistical analysis of these two gene regions (Excoffier, Laval, & Schneider, 2005).

### 2.8 RNA extraction and gene expression analysis

A total of 44 flies (three to seven per population/sex/temperature treatment group) were subjected to microarray‐based gene expression analysis upon completion of their three‐week maturation period (Figure 1, Table 1). Individual flies were mechanically homogenized for total RNA extraction using a NucleoSpin^®^ RNA II Kit (Macherey Nagel). The RNA was normalized to a concentration of 30 ng/μl and submitted to the John A. Burns School of Medicine at the University of Hawaii at Manoa core genetics facility for microarray processing. Briefly, an Agilent Technologies low‐input quickAmp Labeling kit was used to reverse transcribe RNA into cDNA and amplify it in the presence of dye‐labeled nucleotides (Cyanine‐3 CTP). Labeled samples were hybridized at 65°C overnight on a commercially prepared Agilent Technologies (Santa Clara, CA) microarray slide, with males and females loaded on to separate slides. Each microarray contained 14,850 probes, of which 14,766 produced signals and represented 14,319 *D. grimshawi* genes. After hybridization, microarray slides were washed at room temperature (37°C) for 1 min each using Agilent wash buffers, followed by slide scans using Agilent scanner G2565CA. Adherent dye intensities were recorded using Agilent Technologies Feature extraction software. Statistical differences in log_2_ gene expression were measured using a two‐factor ANOVA with main factors source population and temperature treatment and a factor for their interaction. Data were analyzed separately by sex. In cases where the Agilent chip contained multiple probes per gene (828 probes for 383 genes), the probe expression values were averaged across each gene. To adjust for multiple tests, a Benjamini–Hochberg false discovery rate (BH‐FDR) was applied to ANOVA p‐values using a statistical significance threshold of *q* < 0.05 implemented in the R package “stats” v. 3.1.1.

### 2.9 Gene annotation and functional classification

Differentially expressed genes (DEGs) were assigned to gene ontology (GO) categories and tested for overrepresentation utilizing Protein ANalysis THrough Evolutionary Relationships (PANTHER, Thomas et al., 2003, version 13.1 released 2018‐02‐03; overrepresentation test function released 20171205; GO Ontology database released 2018–05–2). The analyzed gene lists were constructed by matching differentially expressed *D. sproati* genes (tracked by *D. grimshawi* FlyBase “Fbgn” identifiers, Appendix S1) to *D. melanogaster* gene orthologs and gene symbols procured from the FlyBase.org file “gene_orthologs_fb_2016_03.tsv.” PANTHER's built‐in Fisher's exact test with FDR multiple test corrections was used to assess statistical significance of overrepresented GO‐Slim categories, applying a threshold cutoff of *q* < 0.05 and *D. melanogaster* as a background genome. To permit direct comparison to *Drosophila* findings by Sørensen, Nielsen, Kruhøffer, Justesen, & Loeschcke, 2005 and PANTHER results, additional pathway enrichment tests were performed in DAVID (Huang, Sherman, & Lempicki, 2009a; Huang, Sherman, & Lempicki, 2009b; v6.8) using the Functional Annotation Tool and Pathway Viewer with built‐in BH‐FDR tests. *D. melanogaster* was again used as a background genome. Additional DEG annotation was performed by procuring gene function information from FlyBase's heat‐shock protein and cognate gene lists and the “jump to gene” query tool, and through peer‐reviewed literature searches for thermal adaptation genes.

## 3 RESULTS

### 3.1 Site descriptions

Mature wet forest tree and tree fern species were found at the center of each kipuka, with smaller individuals of the same species found at the edges prior to the sharp transition to the surrounding recent lava flows. Concurrent temperature measurement during December 2009 identified the low‐elevation site as having higher minimum, maximum, and daytime mean temperatures (Table 2).

**Table 2:** Ecological and climatic description of the two study locations. Trees and tree ferns over 2 m in height or 1 cm DBH were surveyed in four 3 m radius plots at the center and edge of each kipuka. Temperature was measured concurrently from December 16–29, 2009 with measurements made at 5‐min intervals at 2 m off the ground under artificial shade. The daytime means were calculated from 6 a.m. to 6 p.m.

	Low		High	
	Center	Edge	Center	Edge
Site description				
Elevation	1,260		1625	
Forest reserve	Hilo Watershed		Upper Waiakea	
GPS location	*N* 19.677613		*N* 19.665613	
	W −155.290150		W −155.350996	
Indicator tree and fern species				
Total count				
*Metrosideros polymorpha*	19	70	11	43
*Cheirodendron trigynum*	14	4	4	10
*Cibotium spp.*	40	15	39	3
Mean DBH				
*Metrosideros polymorpha*	10.0	7.0	26.2	9.9
*Cheirodendron trigynum*	8.6	4.5	11.2	3.7
Temperature (Dec 16–29, 2009)				
Daytime Mean	12.4	14.5	11.0	12.2
Observed Maximum	18.2	24.9	16.3	18.6
Observed Minimum	4.0	4.3	3.4	3.3

### 3.2 Behavior

**Figure 2:**
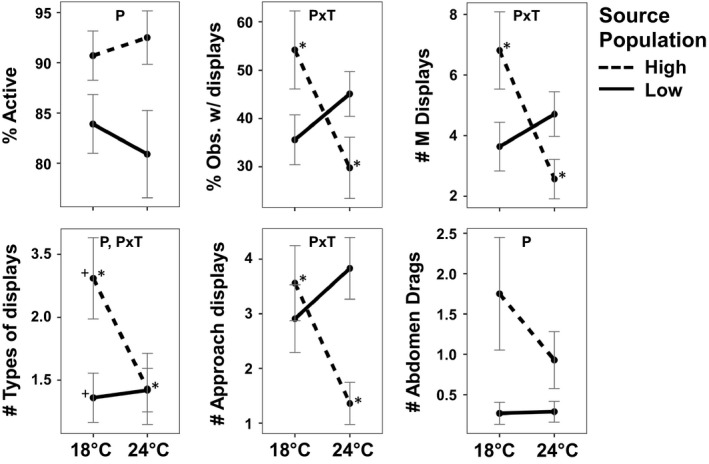
Means and standard errors of behavioral measures of *D. sproati *laboratory groups from low‐ and high‐elevation populations maintained at 18°C or exposed to 24°C during maturation. Significant two‐way ANOVA results are noted in each panel as P (source population), T (temperature treatment), and P  T (interaction). Significant differences between paired groups, as assessed through Tukey tests (*p* < 0.05), are indicated by matching symbols (* or +). *M* = Male‐only display; M:*F* = Male approaching female display

The four representative *D. sproati* laboratory groups (low‐ vs. high‐elevation source population, 18°C vs. 24°C maturation temperature) showed multiple significant differences in behavior that were explained by source population or the interaction between source population and temperature treatment (Figure 2, Table 3). The two‐way ANOVA identified source population alone as accounting for the significant difference in overall activity levels and the frequency of the male‐only abdomen drag display. The interaction of source population and temperature treatment explained significant differences in four behavioral measures: the percent of observations with at least one male display, the total number of male displays per social group, the numbers of types of male displays, and the number of “approach” displays that initiates courtship. Pairwise population comparisons through Tukey tests identified the two high‐elevation populations (18°C and 24°C) to be the primary source of these differences, with the flies that had experienced 24°C during maturation scoring significantly lower on many of the behavioral measures, including overall activity levels and the male‐only abdomen drag display.

**Table 3:** Summary statistics and two‐way ANOVA results for select physiological and behavioral measures of *D. sproati *laboratory groups from low‐ and high‐elevation populations maintained at 18°C or exposed to 24°C during maturation. Statistically significant results (*p*‐value <0.05) are bolded. All tests had 1 degree of freedom. The unit of the male displays is mean total number of displays per courtship group over the six observation periods

Measure		Sex	Mean				Standard error				ANOVA					
			Low elevation		High elevation		Low elevation		High elevation		Source population		Temperature treatment		Interaction	
			18°C	24°C	18°C	24°C	18°C	24°C	18°C	24°C	*F*	*p*‐value	*F*	*p*‐value	*F*	*p*‐value
Physiological																
Heat Knockdown (hr)		M	3.01	1.80	2.52	2.75	0.22	0.25	0.28	0.54	0.49	0.486	2.19	0.141	**4.76**	**0.031**
		F	3.42	2.83	3.59	4.90	0.20	0.27	0.29	0.39	**15.3**	**0.000**	1.59	0.201	**34.4**	**0.001**
Chill coma recovery (min)		M	18.38	19.77	16.21	19.38	0.87	1.00	1.29	1.42	1.23	0.27	**3.95**	**0.049**	0.61	0.438
		F	19.7	19.68	19.21	17.45	1.24	0.69	0.95	0.71	2.21	0.139	0.95	0.332	0.90	0.345
Behavioral																
% Active			83.9	80.9	90.7	92.5	2.92	4.34	2.45	2.66	**6.81**	**0.011**	0.03	0.873	0.46	0.501
% Obs with ≥1 display			35.6	45.1	54.2	29.8	5.17	4.63	8.08	6.32	0.07	0.787	1.61	0.208	**8.39**	**0.005**
# Male displays			3.64	4.71	6.81	2.57	0.80	0.74	1.28	0.65	0.07	0.788	2.70	0.105	**8.90**	**0.004**
# Types of displays			1.36	1.42	2.31	1.43	0.20	0.17	0.32	0.28	**4.31**	**0.042**	3.22	0.077	**4.10**	**0.047**
# Abdomen drag (M)			0.27	0.29	1.75	0.93	0.14	0.13	0.70	0.35	**9.41**	**0.003**	1.22	0.272	1.35	0.249
# Approach (M:F)			2.91	3.83	3.56	1.36	0.62	0.56	0.69	0.39	3.09	0.083	1.12	0.294	**7.76**	**0.007**

### 3.3 Physiology

**Figure 3:**
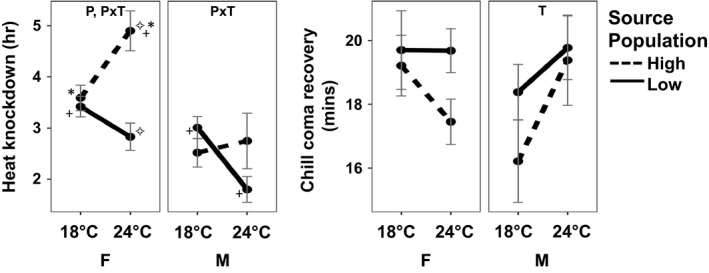
Mean and standard error for heat knockdown resistance and cold chill coma recovery of male (M) and female (F) *D. sproati *from laboratory groups founded from low‐ and high‐elevation populations and exposed to 18°C or 24°C during maturation. Significant two‐way ANOVA results are noted in panels as P (source population), T (temperature treatment), and P  T (interaction). Significant differences between paired groups, as assessed through Tukey tests (*p* < 0.05), are indicated by matching symbols

For heat knockdown resistance, the ANOVA analysis identified the interaction of source population and temperature treatment to be significant for both male and female flies, and as well as population alone for females (Figure 3). In the low‐elevation populations, both male and female flies exposed to 24°C during maturation were less resistant to subsequent heat shock than those maintained at 18°C, while the opposite was true for those from the high‐elevation population. The Tukey test of pairwise differences found that the only significant difference among males for knockdown resistance was between the 18°C and 24°C low‐elevation populations, while for females the 24°C high‐elevation population was significantly different from the other three female groups. For cold chill coma recovery, the ANOVA analysis identified temperature treatment alone was found to be significant in males, with flies exposed 24°C during maturation taking on average longer to recover than flies maintained at 18°C (Table 3, Figure 3).

### 3.4 Population genetic analysis

The two populations were not significantly differentiated (*p* > 0.05) at either the COII or YP1 gene as measured by pairwise *F*_ST_ (COII = 0.001, YP1 = 0.043) or by an exact test of population differentiation (COII = 0.158, YP1 = 0.084). Internal characteristics of the two populations were similar in both loci (Table S2).

### 3.5 Differential gene expression

**Figure 4:**
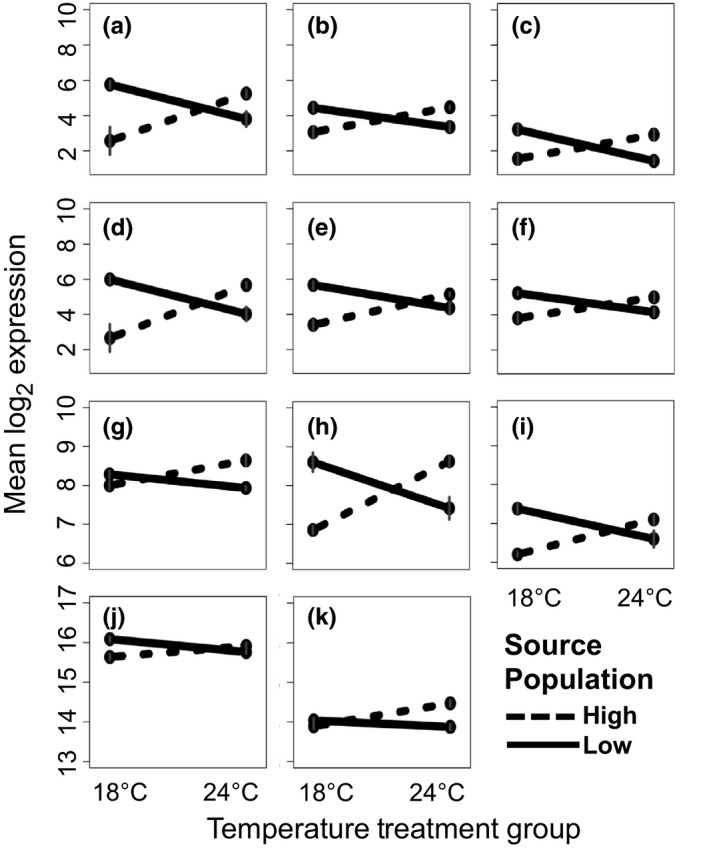
Interaction plots for 11 genes differentially expressed in low‐ and high‐elevation populations of male *Drosophila sproati* and explained by the interaction of source population and temperature treatment. FlyBase IDs and brief functional descriptions with corresponding *D. melanogaster* gene ortholog symbols or functional domain annotations (if available) are as follows: (a) FBgn0130876; (b) FBgn0117568 (CG31784); (c) FBgn0119956 (GH12477, contains domain cytochrome b561/ferric reductase transmembrane); (d) FBgn0132691; (e) FBgn0123024 (transcriptional co‐factor, nab); (f) FBgn0123345 (retinin‐like protein); (g) FBgn0120752 (CG17005); (h) FBgn0128660 (cuticle protein, Cpr49Ad); (i) FBgn0129493 (transcription factor, bap); (j) FBgn0126885 (contains succinate dehydrogenase, subunit C, mitochondrial protein, SdhC, domain); (k) FBgn0119942 (contains hypoxia‐induced protein domain). (a) and (d) contain a palmitoyltransferase, DHHC domain and match to multiple *D. melanogaster* orthologs (CG13029, CG17195–98, CG4956). Error bars are shown in gray

Significant differences in gene expression, explained by source population or the interaction between source population and temperature treatment, were observed for 1730 (12.1% of 14,319) genes in the male group and for 96 (0.7% of 14,319) genes in the female group (Appendix S1). Females and males shared 39 of these DEGs in common. The main factor population explained 99% (1,720) of the male and 100% of the female DEGs. Of the 1,720 male DEGs significant for population, 671 and 1,032 were log_2_ fold upregulated in the low‐ and high‐elevation population groups, respectively. The remaining 17 genes showed patterns of expression consistent with the interaction of population and temperature treatment (see below), despite nonsignificance for that term. Although no significant changes in gene expression were solely attributed to the main factor temperature treatment (males or females), 11 genes in males were significant for the population and temperature treatment interaction term (Figure 4, Table 4; one gene also significant for population in males). In all of these cases, male flies from the low‐elevation population responded to the elevated temperature treatment by down‐regulating gene expression, while males from the high‐elevation population responded to the elevated temperature treatment by up‐regulating gene expression. Due to the limited difference between females across treatment contrasts, and because their expression patterns followed male counterparts across 38 of the 39 genes in common, subsequent analyses are focused on interpretation of the male DEG data set.

**Table 4:** List of differentially expressed genes in male *Drosophila sproati *populations significant for the interaction of source population and temperature treatment (top row only), or significant for source population only and identified as responsive to heat stress in one or more comparison studies. Genes identified as heat responsive in two or more comparison studies are indicated by bold font

ANOVA Factor	Comparative study	Species	Gene symbol
Population x Temperature treatment	n/a	*D. sproati*	bap, CG31784, GH12477, CG17005, Cpr49Ad, CG13029^a^, CG17195–8^a^, CG4956^a^, FBgn0120752 (no gene symbol), CG9921^b^, nab, SdhC^c^
Population	Leemans et al. (2000)	*D. melanogaster*	**DNAJ−1**, Dlc90F, **Eip71CD**, Gpdh, HSC70–3, **HSP23**, **HSP83**, ImpL3, Shark, ovo
Population	Sørensen et al. (2005), Table 3^d^	*D. melanogaster*	aay, Cyp4ac2, CG2254, CG3244, CG5023, CG5804, CG5966, CG6426, CG7916, CG8774, CG9259, CG10383, CG10513, CG10514, CG10680, CG16762, CG16898, CG16985, CG17124, CG18585, **Eip71CD**, **GstD2**, GstD4, **GstD5**, hgo, **HSP23, HSP83**, ImpL3, Pepck, Thor (two paralogs), Trxr−1
Population	Sørensen et al. (2005), Table 4^e^	*D. melanogaster*	Cyp4ac2, DNAJ‐like−1 (**DNAJ−1**), CG1628, **CG4797**, CG5646, **CG15408**, GstE1,** GstD2**,** GstD5**, Hsc70Cb, **HSP83**, **PEK**, raw, Vha26
Population	Uy et al. (2015)	*D. sproati*	Arl5, **CG4797**, CG8665, CG14694, CG31975, fusl, fw, Ilp8, Prat2
Population	Uy et al. (2015)	*D. silvestris*	Alp4, ArfGAP3, Atg1, betaggt‐I, capu, CG5853, CG5958, CG10178, CG10621, CG11123, CG11529, CG11601, CG11854, CG12909, CG12975, **CG15408**, CG31431, CG32191, DCP1, **Droj2**, GIIIspla2, KCNQ, lft, lolal, mRpL17, mRpS6, MTF−1, Nbr, net, Pld, prc, RpLP0‐like, swi2
Population	Uy et al. (2015)	*D. sproati* and *silvestris*	baf, CG11267, CG13748, CG45782, fok, Hipk, **HSP23**, **HSP83**, MFS14, Pat1, Pdk, **PEK**, Slc45–1

a The *Drosophila grimshawi* FlyBase gene identifiers FBgn0130876 and FBgn0132691 match to the same multiple gene symbols and have multiple *D. melanogaster* orthologs.

b Also significant for factor population.

c Associated with mitochondrial function: blp (Roy et al. 2012), Hsc20, SdhC (Uhrigshardt et al., 2010), and CG11267.

d Heat‐responsive genes identified by Sørensen et al., 2005 and common to at least two additional stress response studies listed therein; See Table 3 of Sørensen et al., 2005.

e Genes identified by Sørensen et al., 2005 (see Table 4 of Sørensen et al., 2005) that belong to significant functional groups of genes that respond to heat stress in *Drosophila.*

### 3.6 Gene annotation and functional classification

FlyBase's high level of annotation permitted assignment of *D. melanogaster* gene symbols to 84% (566 of 671) and 93% (964 of 1,032) of the DEGs upregulated in the low‐ and high‐elevation population groups (males only). Based on these gene symbols, 471 and 777 DEGs were assigned to PANTHER GO category terms, after accounting for paralogs (*i.e.,* duplicate genes) and genes with no mapping information. Allocations of these genes to each of the three major GO categories—molecular function, biological process, and cellular component—indicate a greater number and diversity of overrepresented GO terms (*n* = 36) in the high‐elevation relative to the low‐elevation population groups (*n* = 4) (Appendix S2). Several overrepresented GO terms were associated with energy, metabolism, and mitochondria. No statistically enriched pathways were identified through PANTHER analysis, but results for the 1,467 gene symbols recognized by DAVID indicate significant enrichment of 17 KEGG pathways (Table S3), with 8 and 9 overrepresented in low‐ and high‐elevation populations groups, respectively. Most of the overrepresented pathways relate to metabolism (e.g., tryptophan, propanoate, ether lipid, and xenobiotics). The most notable pathway was glutathione metabolism, overrepresented in high‐elevation population groups and associated with heat and cold tolerance pathways in *D. melanogaster* (MacMillan et al., 2016; Sørensen et al., 2005). Approximately 100 DEGs were identified as responsive to temperature stress in other studies (Table 4), along with 18 heat‐shock proteins and cognates (Table 5; some genes in common between tables). Annotations for the 11 DEGs significant for the interaction of source population and temperature treatment (Figure 4, Table 4, Appendix S1) indicate that two are involved in mitochondrial function, two are involved in transcriptional activity, one contains a hypoxia‐induced protein domain (per InterPro, also significant for source population), a cuticle protein, and five genes having unknown function at this point in time.

**Table 5:** Heat‐shock protein family or cognate membership of 18 genes significant for the factor source population and differentially upregulated in low‐ or high‐elevation male *Drosophila sproati* population groups

Heat‐shock protein family/cognates	High elevation	Low elevation
Small heat‐shock protein (HSP20) family	HSP23 (two copies)	—
HSP 40/J‐protein Gene Group	blp^a^, CG2790, CG17187, DnaJ−1, Droj2, Hsc20^a^, P58IPK	CG8476, CG8531
HSP60 Gene Group, Chaperonins, Group I	CG11267^a^	HSP60B
HSP70 Superfamily	Hsc70Cb, HSC70–3	CG2918 (atypical HSP70)
HSP90 Gene Group, Chaperones	HSP83	—
Hsp100 Family	DnaK	—

a Associated with mitochondrial function: blp (Roy et al. 2012), Hsc20 (Uhrigshardt et al., 2010), and CG11267.

## 4 DISCUSSION

This integrated assessment of behavioral, physiological, population genetic, and gene expression measures indicates strong adaptive divergence between two populations of *D. sproati* at distributional extremes along a narrow 365 m elevation gradient. Despite these populations being separated by only 7 km and showing no significant population genetic structure, *D. sproati* males in laboratory populations exhibited opposing adaptive strategies in response to a slight increase in ambient temperature during maturation. Relative to the males from each elevation that matured at a constant 18°C, those from the low‐elevation population that were exposed to 24°C maintained normal levels of courtship but were physiologically more sensitive to subsequent high temperatures, while those from the high‐elevation population that were exposed to 24°C maintained normal heat‐shock resistance but displayed reduced courtship behavior. This divergent response among males was also shown through significant differences in gene expression attributed to the interaction of temperature and population, with the males in the two populations showed a uniformly opposite regulatory response to the low‐intensity temperature increase.

We found surprisingly high levels of behavioral and gene expression differentiation between the laboratory populations from the low‐ and high‐elevation sites irrespective of the temperature treatment. Flies descended from the high‐elevation site were generally more active than those from the low‐elevation site, and the males were more likely to perform a solitary pre‐courtship behavioral display. The males from the high‐elevation population site also demonstrated statistical overrepresentation of genes related to energy production and consumption, enzymatic activity, and gene regulation. In total, transcriptome profiles showed that nearly 12% of the functional genes targeted in the array were differentially expressed among males due to the source population, while none were associated with the temperature treatment alone and less than 0.1% were related to the interaction between the two factors.

Gene expression studies of temperature tolerance often assess short‐term responses to high‐intensity heat shocks (e.g., Leemans et al., 2000, Sørensen et al., 2005, Boardman, Mitchell, Terblanche, Jesper, & Sørensen, 2018), rather than long‐term responses to low‐intensity temperature differences. Both types of stress are associated with anthropogenic climate change and can drive adaptation, but it might be assumed that they would present radically different selective pressures and would induce distinct adaptive responses. However, we found that more than 100 of the DEGs significant for source population in this study were affected by heat shock in other *Drosophila *studies (Table 4)*. *These include 13.5% of the 74 heat‐shock‐inducible genes in identified by Leemans et al., 2000 and 15.6% of the 199 heat‐responsive genes identified by Sørensen et al., 2005, both in *D. melanogaster*. Our data set also includes 20.1% of 106 DEGs identified within *D. sproati* subjected to a one‐hour treatment at 25**°**C, as compared to those at a controlled temperature of 16**°**C, and 18.3% of 246 DEGs found in *D. silvestris *after the same one‐hour, 25**°**C treatment (Table 4, Uy et al., 2015). That certain genes were differentially expressed across multiple *Drosophila* temperature treatment studies and between our low‐ and high‐elevation population groups suggests these genes have important roles in adaptation to thermal conditions.

The identification of HSPs and cognates differentially expressed between low‐ and high‐elevation populations and common to other temperature tolerance *Drosophila* studies may aid in pinpointing proteins with broad roles in climate adaptation. HSPs and their cognates are part of the protein quality system that assists in degradation of denatured or aggregated proteins and is mounted when organisms are exposed to environmental stressors, including oxidative, physical activity, heavy metals, and temperature (Sørensen, Kristensen, & Loeschcke, 2003). We found that genes HSP23 and HSP83 were differentially expressed in low‐and high‐elevation *D. sproati *populations, and heat‐shocked *D. sproati *(Uy et al., 2015 *)*, *D. silvestris* (Uy et al., 2015), and *D. melanogaster* (Leemans et al., 2000; Sørensen et al., 2005). Also notable were two DEGs belonging to the HSP70 superfamily that showed differential expression in heat‐shocked *D. melanogaster: *HSC70–3 (Leemans et al., 2000) and HSC70Cb (Sørensen et al., 2005). We also found nine HSP40/J‐domain proteins, which help HSP70s target to their substrates and control the ATPase cycle (Mayer & Bukau, 2005). A subset of the HSPs/cognates are associated with mitochondria function, including iron–sulfur cluster assembly, and were co‐expressed with male DEGs IscU, an iron–sulfur cluster assembly enzyme, and SdhC, a subunit of the mitochondrial complex (Uhrigshardt et al. 2010). In sum, HSPs and cognates that show commonality across studies may have particularly important roles in the ability of these *Drosophila* to cope with both short intense heat shocks and more subtle long‐term increases in ambient temperature, both of which might be required to adapt to changing climatic conditions.

In addition to HSPs, several non‐HSP genes associated with temperature tolerance in other studies were differentially expressed between the low‐ and high‐elevation male population groups. Most notably, Glutathione S transferase (GstD2 and GstD5) genes have been identified as heat resistance genes through multiple lines of research: QTL mapping, differential gene expression, and gene deletion approaches (Leemans et al., 2000, Sørensen et al., 2005, Takahashi, Okada, & Teramura, 2011, see Table 5, this study). The GstD gene is functionally involved in Glutathione metabolism, a pathway enriched in the higher elevation population and that appears to have a role in cold and heat tolerance (MacMillan et al., 2016; Sørensen et al., 2005). This pathway is also speculated to correspond to natural climatic fluctuations in plants (Milner, Reade, & Cobb, 2007). Also notable are genes in the mitogen‐activated protein kinase (MAPK) family, which are associated with general stress‐responses and are induced by heat, oxidative, and UV light stress (Takahashi et al., 2011). We found one MAPK gene, *Extracellularly regulated kinase 7* (Erk7), to be differentially upregulated in the low‐elevation population. Clearly, temperature is only one of many selective pressures that drives adaptive divergence, and assessing ecological responses to anthropogenic climate change may benefit from broader surveys of gene expression responses under a variety of environmental conditions (*e.g.*, humidity, UV exposure). Our findings add to the growing body of literature that identifies candidate genes and biochemical pathways that may underlie physiological adaptations to local environmental conditions.

The evidence for adaptive divergence between the low‐ and high‐elevation populations is supported by significant differences in behavior, physiology and gene expression, and no significant population genetic structure as estimated using the COII and YP1 genes in the wild‐caught flies collected from the source populations. Further studies are needed to determine whether the underlying DNA sequence divergence between populations result in the important differences in behavioral and physiological traits and thus represent rapid local adaptation following recent isolation. Differentiation in the presence of ongoing gene flow is known to occur in *Drosophila* species, including natural populations of *D. melanogaster* and *D. buzzatii* (Michalak et al., 2001; Sarup, Sørensen, Dimitrov, Barker, & Loeschcke, 2006). An additional, unexplored possibility is that population differences are explained by epigenetic inheritance, which would allow for plasticity over the long term. However, the populations were housed in the same common environment room for five generations before the initiation of this study, which should limit any epigenetic effects. Since *D. sproati* are large and able fliers, and the previous island‐wide study did not find genetic structuring within the larger wet forest region on the eastern side of Hawaii Island (Eldon et al., 2013), divergent adaptation amidst ongoing gene flow appears to be the most parsimonious explanation for our observations.

This observation of potential adaptive divergence without differentiation at putatively neutral loci could also be explained if the laboratory populations were not in fact representative of the wild populations. This might occur from strong founder effects or selective bottlenecks during the establishment of the laboratory populations, or from dramatic genetic drift during the five generations in the laboratory. However, a previous survey of wild‐caught mature females from multiple Hawaiian *Drosophila* species found 99.6% insemination across the group, and 100% among *D. sproati* females (Kambysellis & Craddock, 1991). Another survey found *D. sproati* females to have the highest potential fecundity of the sampled Hawaiian *Drosophila *species, having on average 65 ovarioles per fly and 1–3 mature eggs per ovarioles (Kambysellis & Heed, 1971). The likelihood of a strong founder effect from the 20 + founding females per laboratory population or dramatic divergent drift in the five subsequent generations is therefore low.

In this study, we found evidence that indicates there is adaptive divergence of populations of *D. sproati* separated by only 7 kilometers and 365 m in elevation, but no evidence of population differentiation at genes commonly used to estimate population genetic structure. This finding suggests that the ecological responses to climatic differences may occur at finer scales and be more complex than is often assumed. For example, a common assumption is that close proximity or a lack of differentiation at putatively neutral gene sequences indicates lack of adaptive population divergence, but clearly that is not the case in this study. This finding is relevant to conservation planning efforts, which often use the former assumptions to determine management units. In addition, rather than observing a simple range shift, where one population was more stressed and the other less so, this study found that the marginal *D. sproati* populations are making opposing adaptive trade‐offs under increasing ambient temperatures. Such results caution against the reliance on neutral loci alone or broad ecological assumptions when planning conservation actions. Instead, the findings of this study suggest that we need to adopt a more precise and integrated approach to investigating ecological responses to global climatic change and a more data‐driven approach to drawing conclusions, predictions, and management recommendations.

## CONFLICT OF INTEREST

None declared.

## AUTHORS’ CONTRIBUTIONS

JE collected and maintained the *D. sproati *population used in this study and conducted the behavioral, physiological, and neutral genetic analysis. DP and RB conducted the gene expression analysis. RB modeled and analyzed gene expression profiles and performed functional annotation. DP guided the project design and supervised all research activities. All authors contributed critically to the drafts and gave final approval for publication.

## Data Availability

The microarray data discussed in this publication have been deposited in NCBI's Gene Expression Omnibus and are accessible through GEO Series accession number GSE122959. The D. sproati collection locations are available through DRYAD (https://doi.org/10.5061/dryad.cn946) and the COII and YP1 sequence data through GenBank (COII: JX455020‐JX455050, YP1: JX454999‐JX455019).
