# Supplementary material for: Hawaiian picture‐winged Drosophila exhibit adaptive population divergence along a narrow climatic gradient on Hawaii Island
Source: Ecol Evol. 2019 Feb 18;9(5):2436–48. doi: 10.1002/ece3.4844 (PMC6405895; doi:10.1002/ece3.4844)
Supplement: Supplementary file 1 [file ECE3-9-2436-s001.docx]

| Gender | *D. grimshawii* Flybase gene identifier | Population with Gene Upregulated | Significant 2-factor ANOVA term | *D. melangaster* ortholog | Gene symbol | | Microarray # | |
| --- | --- | --- | --- | --- | --- | --- | --- | --- |
| female | FBgn0117895 | HE | E | FBgn0032266 | CG18302 | 2 | |  |
| female | FBgn0118060 | LE | E | FBgn0031683 | CG4230 | 2 | |  |
| female | FBgn0118411 | LE | E | FBgn0031782 | WDR79 | 2 | |  |
| female | FBgn0118462 | HE | E | FBgn0003087 | pim | 2 | |  |
| female | FBgn0119143 | LE | E | FBgn0031602 | CG15431 | 2 | |  |
| female | FBgn0119283 | LE | E | FBgn0030151 | CG1354 | 2 | |  |
| female | FBgn0119337 | LE | E | ------ | ------ | 2 | |  |
| female | FBgn0119355 | HE | E | ------ | ------ | 2 | |  |
| female | FBgn0120142 | LE | E | FBgn0039945 | CG17159 | 2 | |  |
| female | FBgn0120242 | HE | E | FBgn0030603 | CG5541 | 2 | |  |
| female | FBgn0120589 | HE | E | FBgn0028950 | CG15255 | 2 | |  |
| female | FBgn0120738 | HE | E | FBgn0259716 | CG42370 | 2 | |  |
| female | FBgn0120889 | HE | E | FBgn0003087 | pim | 2 | |  |
| female | FBgn0121012 | LE | E | FBgn0031700 | CG14022 | 2 | |  |
| female | FBgn0121096 | HE | E | ----- | GH13620 | 2 | |  |
| female | FBgn0121250 | LE | E | FBgn0032612 | CG13282 | 2 | |  |
| female | FBgn0121356 | HE | E | FBgn0003087 | pim | 2 | |  |
| female | FBgn0121380 | LE | E | FBgn0002922 | nau | 2 | |  |
| female | FBgn0121777 | HE | E | FBgn0039648 | CG14515 | 2 | |  |
| female | FBgn0121905 | LE | E | FBgn0262737 | mub | 2 | |  |
| female | FBgn0122447 | HE | E | FBgn0004865 | Eip78C | 2 | |  |
| female | FBgn0122643 | HE | E | FBgn0004619 | GluRIA | 2 | |  |
| female | FBgn0122815 | HE | E | FBgn0036749 | CG7460 | 2 | |  |
| female | FBgn0122815 | HE | E | FBgn0036750 | CG6034 | 2 | |  |
| female | FBgn0122970 | LE | E | FBgn0265089 | eIF4E-3 | 2 | |  |
| female | FBgn0123046 | LE | E | FBgn0023479 | Tequila | 2 | |  |
| female | FBgn0123185 | LE | E | ------ | ------ | 2 | |  |
| female | FBgn0123241 | HE | E | ----- | GH15770 | 2 | |  |
| female | FBgn0123278 | LE | E | FBgn0037369 | CG2100 | 2 | |  |
| female | FBgn0123465 | HE | E | FBgn0035102 | CG7049 | 2 | |  |
| female | FBgn0123743 | LE | E | FBgn0263973 | jv | 2 | |  |
| female | FBgn0123898 | LE | E | FBgn0035464 | CG12006 | 2 | |  |
| female | FBgn0124125 | LE | E | ----- | GH16654 | 2 | |  |
| female | FBgn0124393 | HE | E | FBgn0036239 | Pop2 | 2 | |  |
| female | FBgn0124734 | HE | E | ------ | ------ | 2 | |  |
| female | FBgn0124974 | HE | E | FBgn0038966 | pinta | 2 | |  |
| female | FBgn0125022 | LE | E | ------ | ------ | 2 | |  |
| female | FBgn0125472 | HE | E | FBgn0036749 | CG7460 | 2 | |  |
| female | FBgn0125472 | HE | E | FBgn0036750 | CG6034 | 2 | |  |
| female | FBgn0125514 | LE | E | FBgn0039277 | CG13650 | 2 | |  |
| female | FBgn0125531 | LE | E | FBgn0015589 | Apc | 2 | |  |
| female | FBgn0125665 | LE | E | FBgn0263490 | mld | 2 | |  |
| female | FBgn0125852 | HE | E | FBgn0039751 | CG1983 | 2 | |  |
| female | FBgn0126013 | HE | E | FBgn0038147 | CCHa2 | 2 | |  |
| female | FBgn0126428 | HE | E | FBgn0039471 | CG6295 | 2 | |  |
| female | FBgn0126489 | HE | E | FBgn0014930 | CG2846 | 2 | |  |
| female | FBgn0126701 | LE | E | ------ | ------ | 2 | |  |
| female | FBgn0126747 | LE | E | FBgn0001112 | Gld | 2 | |  |
| female | FBgn0126860 | HE | E | FBgn0031489 | CG17224 | 2 | |  |
| female | FBgn0127077 | HE | E | FBgn0038752 | CG4462 | 2 | |  |
| female | FBgn0127176 | LE | E | FBgn0025637 | SkpA | 2 | |  |
| female | FBgn0127289 | HE | E | FBgn0010590 | Prosbeta1 | 2 | |  |
| female | FBgn0127466 | LE | E | FBgn0035024 | CG11414 | 2 | |  |
| female | FBgn0127469 | HE | E | FBgn0035028 | Start1 | 2 | |  |
| female | FBgn0127577 | HE | E | FBgn0028426 | JhI-1 | 2 | |  |
| female | FBgn0127713 | LE | E | FBgn0033465 | Etf-QO | 2 | |  |
| female | FBgn0127718 | LE | E | FBgn0028494 | CG6424 | 2 | |  |
| female | FBgn0127722 | HE | E | FBgn0033367 | PPO2 | 2 | |  |
| female | FBgn0128011 | LE | E | ------ | ------ | 2 | |  |
| female | FBgn0128035 | LE | E | FBgn0020930 | Dgkepsilon | 2 | |  |
| female | FBgn0128097 | LE | E | FBgn0033523 | CG12895 | 2 | |  |
| female | FBgn0128147 | LE | E | ------ | ------ | 2 | |  |
| female | FBgn0128167 | LE | E | ------ | ------ | 2 | |  |
| female | FBgn0128208 | HE | E | FBgn0034160 | CG5550 | 2 | |  |
| female | FBgn0128302 | LE | E | FBgn0034511 | GNBP-like3 | 2 | |  |
| female | FBgn0128302 | LE | E | FBgn0033301 | CG12780 | 2 | |  |
| female | FBgn0128316 | LE | E | FBgn0010339 | 128up | 2 | |  |
| female | FBgn0128398 | LE | E | FBgn0040502 | CG8343 | 2 | |  |
| female | FBgn0128488 | LE | E | FBgn0050443 | Opbp | 2 | |  |
| female | FBgn0128560 | LE | E | FBgn0033302 | Cyp6a14 | 2 | |  |
| female | FBgn0128598 | HE | E | FBgn0034670 | CG13488 | 2 | |  |
| female | FBgn0128646 | LE | E | FBgn0034624 | CG17974 | 2 | |  |
| female | FBgn0128683 | LE | E | FBgn0034883 | CG17664 | 2 | |  |
| female | FBgn0128830 | HE | E | FBgn0034731 | CG10384 | 2 | |  |
| female | FBgn0128875 | LE | E | FBgn0034232 | CG4866 | 2 | |  |
| female | FBgn0128885 | LE | E | FBgn0260866 | dnr1 | 2 | |  |
| female | FBgn0128888 | HE | E | FBgn0033479 | CG2292 | 2 | |  |
| female | FBgn0129032 | HE | E | FBgn0039463 | CG18472 | 2 | |  |
| female | FBgn0129201 | HE | E | FBgn0283437 | PPO1 | 2 | |  |
| female | FBgn0129220 | LE | E | ----- | GH21759 | 2 | |  |
| female | FBgn0129381 | LE | E | FBgn0262560 | wcd | 2 | |  |
| female | FBgn0129407 | LE | E | ------ | ------ | 2 | |  |
| female | FBgn0129445 | LE | E | FBgn0034529 | FAM21 | 2 | |  |
| female | FBgn0129924 | LE | E | ----- | GH22466 | 2 | |  |
| female | FBgn0131158 | HE | E | FBgn0031373 | CG15358 | 2 | |  |
| female | FBgn0131348 | LE | E | ------ | ------ | 2 | |  |
| female | FBgn0131709 | LE | E | FBgn0023529 | CG2918 | 2 | |  |
| female | FBgn0131848 | LE | E | ------ | ------ | 2 | |  |
| female | FBgn0131966 | HE | E | ------ | ------ | 2 | |  |
| female | FBgn0132035 | LE | E | FBgn0000382 | csw | 2 | |  |
| female | FBgn0132115 | HE | E | ------ | ------ | 2 | |  |
| female | FBgn0132116 | LE | E | FBgn0030245 | CG1637 | 2 | |  |
| female | FBgn0132117 | HE | E | ------ | ------ | 2 | |  |
| female | FBgn0132152 | HE | E | FBgn0025835 | CG17707 | 2 | |  |
| female | FBgn0132221 | LE | E | FBgn0030321 | CG1703 | 2 | |  |
| female | FBgn0132351 | LE | E | FBgn0030528 | CG11095 | 2 | |  |
| female | FBgn0132517 | HE | E | ------ | ------ | 2 | |  |
| female | FBgn0132573 | HE | E | FBgn0011705 | rost | 2 | |  |
| female | FBgn0132706 | HE | E | FBgn0259716 | CG42370 | 2 | |  |
| male | FBgn0117568 | LE/HE | T x E | FBgn0051784 | CG31784 | 1 | |  |
| male | FBgn0119956 | LE/HE | T x E | --- | GH12477 | 1 | |  |
| male | FBgn0120752 | LE/HE | T x E | FBgn0032109 | CG17005 | 1 | |  |
| male | FBgn0123024 | LE/HE | T x E | FBgn0259986 | nab | 1 | |  |
| male | FBgn0123345 | LE/HE | T x E | --- | --- | 1 | |  |
| male | FBgn0126885 | LE/HE | T x E | FBgn0037873 | SdhC | 1 | |  |
| male | FBgn0128660 | LE/HE | T x E | FBgn0033726 | Cpr49Ad | 1 | |  |
| male | FBgn0129493 | LE/HE | T x E | FBgn0004862 | bap | 1 | |  |
| male | FBgn0130876 | LE/HE | T x E | FBgn0036670 | CG13029, CG17195-8, CG4956 | 1 | |  |
| male | FBgn0132691 | LE/HE | T x E | FBgn0036670, FBgn0039366-70 | CG13029, CG17195-98, CG4956 | 1 | |  |
| male | FBgn0119942 | LE/HE | E, T x E | FBgn0030743 | hip | 1 | |  |
| male | FBgn0020168 | LE | E | FBgn0004583 | ex | 1 | |  |
| male | FBgn0020177 | HE | E | FBgn0038717 | CG17751 | 1 | |  |
| male | FBgn0020177 | HE | E | FBgn0038718 | CG17752 | 1 | |  |
| male | FBgn0083757 | HE | E | FBgn0020506 | Amyrel | 1 | |  |
| male | FBgn0117537 | HE | E | FBgn0031312 | Tango14 | 1 | |  |
| male | FBgn0117539 | HE | E | FBgn0002931 | net | 1 | |  |
| male | FBgn0117545 | LE | E | FBgn0053107 | CG33107 | 1 | |  |
| male | FBgn0117553 | HE | E | FBgn0031929 | CG18585 | 1 | |  |
| male | FBgn0117562 | HE | E | FBgn0032634 | Rpb11 | 1 | |  |
| male | FBgn0117563 | LE | E | FBgn0032633 | Lrch | 1 | |  |
| male | FBgn0117580 | HE | E | --- | --- | 1 | |  |
| male | FBgn0117590 | HE | E | FBgn0020443 | Elf | 1 | |  |
| male | FBgn0117602 | HE | E | FBgn0032449 | CG17036 | 1 | |  |
| male | FBgn0117605 | LE | E | FBgn0053003 | CG33003 | 1 | |  |
| male | FBgn0117607 | LE/HE | E | FBgn0085207 | CG34178 | 1 | |  |
| male | FBgn0117609 | HE | E | FBgn0031603 | CG15432 | 1 | |  |
| male | FBgn0117614 | LE | E | FBgn0263355 | CG31688 | 1 | |  |
| male | FBgn0117616 | HE | E | FBgn0032429 | CG5446 | 1 | |  |
| male | FBgn0117620 | LE | E | FBgn0032422 | atilla | 1 | |  |
| male | FBgn0117636 | HE | E | FBgn0032643 | GCS2beta | 1 | |  |
| male | FBgn0117647 | LE | E | FBgn0032699 | CG10383 | 1 | |  |
| male | FBgn0117672 | HE | E | FBgn0015797 | Rab6 | 1 | |  |
| male | FBgn0117675 | HE | E | FBgn0032408 | CG6712 | 1 | |  |
| male | FBgn0117687 | HE | E | FBgn0261068 | LSm7 | 1 | |  |
| male | FBgn0117695 | LE | E | FBgn0261560 | Thor | 1 | |  |
| male | FBgn0117697 | LE | E | FBgn0261560 | Thor | 1 | |  |
| male | FBgn0117698 | HE | E | FBgn0017457 | U2af38 | 1 | |  |
| male | FBgn0117704 | LE | E | FBgn0031239 | CG17075 | 1 | |  |
| male | FBgn0117710 | HE | E | FBgn0031232 | CG11617 | 1 | |  |
| male | FBgn0117718 | LE | E | FBgn0000256 | capu | 1 | |  |
| male | FBgn0117746 | LE | E | --- | --- | 1 | |  |
| male | FBgn0117747 | LE | E | FBgn0261608 | RpL37A | 1 | |  |
| male | FBgn0117759 | HE | E | --- | --- | 1 | |  |
| male | FBgn0117763 | LE | E | FBgn0031971 | Sirup | 1 | |  |
| male | FBgn0117774 | HE | E | FBgn0000318 | cl | 1 | |  |
| male | FBgn0117808 | HE | E | FBgn0031344 | CG7420 | 1 | |  |
| male | FBgn0117824 | HE | E | FBgn0032479 | CG16974 | 1 | |  |
| male | FBgn0117843 | HE | E | FBgn0027779 | VhaSFD | 1 | |  |
| male | FBgn0117849 | LE | E | FBgn0261836 | Msp300 | 1 | |  |
| male | FBgn0117856 | LE | E | FBgn0033136 | Tsp42Eo | 1 | |  |
| male | FBgn0117858 | HE | E | FBgn0031875 | CG3430 | 1 | |  |
| male | FBgn0117887 | HE | E | FBgn0032913 | CG9259 | 1 | |  |
| male | FBgn0117888 | HE | E | FBgn0032914 | CG14397 | 1 | |  |
| male | FBgn0117890 | HE | E | FBgn0032916 | CG9257 | 1 | |  |
| male | FBgn0117900 | LE | E | FBgn0032276 | CG17098 | 1 | |  |
| male | FBgn0117912 | LE | E | FBgn0032125 | Cpr30B | 1 | |  |
| male | FBgn0117914 | LE | E | FBgn0032127 | CG13114 | 1 | |  |
| male | FBgn0117921 | LE | E | FBgn0004867 | RpS2 | 1 | |  |
| male | FBgn0117922 | HE | E | FBgn0032154 | mtDNA-helicase | 1 | |  |
| male | FBgn0117929 | LE | E | FBgn0038348 | AOX2 | 1 | |  |
| male | FBgn0117946 | HE | E | FBgn0002564 | Lsp1gamma | 1 | |  |
| male | FBgn0117951 | LE | E | --- | --- | 1 | |  |
| male | FBgn0117955 | HE | E | FBgn0031519 | CG3326 | 1 | |  |
| male | FBgn0117958 | LE | E | FBgn0032260 | CG13144 | 1 | |  |
| male | FBgn0117961 | HE | E | FBgn0263051 | CG43346 | 1 | |  |
| male | FBgn0117965 | LE | E | FBgn0031939 | CG13796 | 1 | |  |
| male | FBgn0117965 | LE | E | FBgn0260479 | CG31904 | 1 | |  |
| male | FBgn0117974 | HE | E | FBgn0032801 | CG10165 | 1 | |  |
| male | FBgn0117976 | HE | E | FBgn0031284 | CG3876 | 1 | |  |
| male | FBgn0117988 | LE | E | FBgn0031264 | CG11835 | 1 | |  |
| male | FBgn0117994 | HE | E | FBgn0043364 | cbt | 1 | |  |
| male | FBgn0118005 | LE | E | FBgn0263079 | CG43338 | 1 | |  |
| male | FBgn0118033 | HE | E | FBgn0031312 | Tango14 | 1 | |  |
| male | FBgn0118050 | HE | E | FBgn0031455 | Snapin | 1 | |  |
| male | FBgn0118051 | HE | E | FBgn0031454 | CG9960 | 1 | |  |
| male | FBgn0118058 | HE | E | FBgn0031436 | ND-B17.2 | 1 | |  |
| male | FBgn0118070 | HE | E | FBgn0051676 | CG31676 | 1 | |  |
| male | FBgn0118072 | LE | E | FBgn0032897 | CG9336 | 1 | |  |
| male | FBgn0118078 | HE | E | FBgn0031689 | Cyp28d1 | 1 | |  |
| male | FBgn0118095 | HE | E | FBgn0260407 | mRpS23 | 1 | |  |
| male | FBgn0118103 | LE | E | FBgn0028540 | CG9008 | 1 | |  |
| male | FBgn0118112 | LE | E | FBgn0032521 | CG7110 | 1 | |  |
| male | FBgn0118120 | HE | E | FBgn0032483 | CG15482 | 1 | |  |
| male | FBgn0118126 | HE | E | FBgn0032753 | CG17572 | 1 | |  |
| male | FBgn0118140 | LE | E | --- | --- | 1 | |  |
| male | FBgn0118148 | LE | E | FBgn0032167 | CG5853 | 1 | |  |
| male | FBgn0118152 | LE | E | FBgn0032130 | CG3838 | 1 | |  |
| male | FBgn0118157 | HE | E | FBgn0040070 | Trx-2 | 1 | |  |
| male | FBgn0118163 | LE | E | FBgn0031474 | CG2991 | 1 | |  |
| male | FBgn0118166 | HE | E | FBgn0051950 | CG31950 | 1 | |  |
| male | FBgn0118176 | HE | E | FBgn0031732 | CG11149 | 1 | |  |
| male | FBgn0118188 | LE | E | FBgn0046704 | Liprin-alpha | 1 | |  |
| male | FBgn0118199 | LE | E | FBgn0086265 | psd | 1 | |  |
| male | FBgn0118200 | LE | E | FBgn0003979 | Vm26Aa | 1 | |  |
| male | FBgn0118218 | LE | E | FBgn0031573 | CG3407 | 1 | |  |
| male | FBgn0118222 | HE | E | FBgn0036767 | CG16775 | 1 | |  |
| male | FBgn0118224 | HE | E | FBgn0036767 | CG16775 | 1 | |  |
| male | FBgn0118225 | HE | E | --- | --- | 1 | |  |
| male | FBgn0118226 | LE | E | FBgn0031803 | ppk14 | 1 | |  |
| male | FBgn0118247 | LE | E | FBgn0040211 | hgo | 1 | |  |
| male | FBgn0118251 | HE | E | FBgn0032050 | CG13096 | 1 | |  |
| male | FBgn0118253 | HE | E | FBgn0026718 | fu12 | 1 | |  |
| male | FBgn0118268 | HE | E | FBgn0015776 | nrv1 | 1 | |  |
| male | FBgn0118302 | HE | E | FBgn0283659 | THG | 1 | |  |
| male | FBgn0118312 | LE | E | --- | --- | 1 | |  |
| male | FBgn0118317 | HE | E | FBgn0261437 | CSN8 | 1 | |  |
| male | FBgn0118321 | HE | E | FBgn0032030 | Wdr82 | 1 | |  |
| male | FBgn0118335 | HE | E | FBgn0016054 | phr6-4 | 1 | |  |
| male | FBgn0118346 | LE | E | --- | --- | 1 | |  |
| male | FBgn0118347 | LE | E | FBgn0031374 | Wdr62 | 1 | |  |
| male | FBgn0118368 | HE | E | FBgn0032026 | CG7627 | 1 | |  |
| male | FBgn0118368 | HE | E | FBgn0032908 | CG9270 | 1 | |  |
| male | FBgn0118368 | HE | E | FBgn0051792 | CG31792 | 1 | |  |
| male | FBgn0118368 | HE | E | FBgn0051793 | CG31793 | 1 | |  |
| male | FBgn0118372 | HE | E | FBgn0002022 | Catsup | 1 | |  |
| male | FBgn0118377 | LE | E | FBgn0032749 | Phlpp | 1 | |  |
| male | FBgn0118389 | HE | E | FBgn0051956 | pgant4 | 1 | |  |
| male | FBgn0118390 | HE | E | FBgn0051956 | pgant4 | 1 | |  |
| male | FBgn0118391 | HE | E | FBgn0040228 | DCTN5-p25 | 1 | |  |
| male | FBgn0118395 | HE | E | FBgn0041713 | yellow-c | 1 | |  |
| male | FBgn0118398 | HE | E | --- | --- | 1 | |  |
| male | FBgn0118404 | HE | E | FBgn0031741 | CG11034 | 1 | |  |
| male | FBgn0118434 | HE | E | FBgn0043002 | Chrac-14 | 1 | |  |
| male | FBgn0118438 | LE | E | FBgn0262029 | d | 1 | |  |
| male | FBgn0118439 | HE | E | FBgn0032052 | PIG-U | 1 | |  |
| male | FBgn0118462 | HE | E | FBgn0003087 | pim | 1 | |  |
| male | FBgn0118470 | LE | E | FBgn0259714 | DIP-epsilon | 1 | |  |
| male | FBgn0118471 | HE | E | FBgn0031805 | CG9505 | 1 | |  |
| male | FBgn0118474 | HE | E | FBgn0031801 | CG9498 | 1 | |  |
| male | FBgn0118491 | LE | E | FBgn0043362 | bchs | 1 | |  |
| male | FBgn0118493 | HE | E | FBgn0001128 | Gpdh | 1 | |  |
| male | FBgn0118496 | LE | E | FBgn0003980 | Vm26Ab | 1 | |  |
| male | FBgn0118522 | HE | E | FBgn0028940 | Cyp28a5 | 1 | |  |
| male | FBgn0118523 | LE | E | FBgn0031730 | CG7236 | 1 | |  |
| male | FBgn0118533 | HE | E | FBgn0051694 | CG31694 | 1 | |  |
| male | FBgn0118540 | LE | E | FBgn0032129 | jp | 1 | |  |
| male | FBgn0118541 | HE | E | FBgn0032166 | CG4619 | 1 | |  |
| male | FBgn0118549 | HE | E | ------ | ------ | 1 | |  |
| male | FBgn0118554 | LE | E | FBgn0032178 | Spn31A | 1 | |  |
| male | FBgn0118570 | HE | E | ----- | ----- | 1 | |  |
| male | FBgn0118573 | HE | E | FBgn0032486 | CG5705 | 1 | |  |
| male | FBgn0118577 | LE | E | FBgn0032523 | CG16956 | 1 | |  |
| male | FBgn0118601 | LE | E | --- | --- | 1 | |  |
| male | FBgn0118602 | LE | E | FBgn0028534 | CG7916 | 1 | |  |
| male | FBgn0118610 | LE | E | FBgn0027929 | NimB1 | 1 | |  |
| male | FBgn0118611 | LE | E | FBgn0028936 | NimB5 | 1 | |  |
| male | FBgn0118613 | HE | E | FBgn0027348 | bgm | 1 | |  |
| male | FBgn0118620 | LE | E | FBgn0261563 | wb | 1 | |  |
| male | FBgn0118630 | LE | E | --- | --- | 1 | |  |
| male | FBgn0118635 | HE | E | FBgn0031684 | ND-13A | 1 | |  |
| male | FBgn0118666 | LE | E | FBgn0031313 | CG5080 | 1 | |  |
| male | FBgn0118671 | LE | E | FBgn0031645 | CG3036 | 1 | |  |
| male | FBgn0118683 | HE | E | FBgn0032668 | CG17681 | 1 | |  |
| male | FBgn0118693 | LE | E | FBgn0032682 | grnd | 1 | |  |
| male | FBgn0118694 | HE | E | FBgn0032684 | CG10178 | 1 | |  |
| male | FBgn0118700 | LE | E | FBgn0011244 | Hsp60B | 1 | |  |
| male | FBgn0118706 | HE | E | FBgn0031256 | CG4164 | 1 | |  |
| male | FBgn0118720 | LE | E | FBgn0041250 | Gr21a | 1 | |  |
| male | FBgn0118723 | LE | E | FBgn0086130 | Dbp21E2 | 1 | |  |
| male | FBgn0118742 | HE | E | FBgn0032259 | CG6144 | 1 | |  |
| male | FBgn0118743 | HE | E | FBgn0031517 | CG15406 | 1 | |  |
| male | FBgn0118744 | LE | E | FBgn0031518 | CG3277 | 1 | |  |
| male | FBgn0118745 | HE | E | FBgn0031520 | CG8837 | 1 | |  |
| male | FBgn0118747 | HE | E | FBgn0031522 | CG3285 | 1 | |  |
| male | FBgn0118748 | HE | E | --- | --- | 1 | |  |
| male | FBgn0118749 | HE | E | FBgn0031523 | CG15408 | 1 | |  |
| male | FBgn0118754 | LE | E | --- | --- | 1 | |  |
| male | FBgn0118770 | LE | E | FBgn0032965 | CG11629 | 1 | |  |
| male | FBgn0118785 | HE | E | FBgn0259713 | CG42367 | 1 | |  |
| male | FBgn0118787 | HE | E | FBgn0032297 | CG17124 | 1 | |  |
| male | FBgn0118790 | HE | E | FBgn0033000 | CG14464 | 1 | |  |
| male | FBgn0118791 | LE | E | --- | --- | 1 | |  |
| male | FBgn0118800 | HE | E | --- | --- | 1 | |  |
| male | FBgn0118814 | HE | E | FBgn0053508 | ppk13 | 1 | |  |
| male | FBgn0118816 | HE | E | FBgn0053510 | CG33510 | 1 | |  |
| male | FBgn0118817 | HE | E | FBgn0053511 | CG33511 | 1 | |  |
| male | FBgn0118830 | LE | E | FBgn0051823 | CG31823 | 1 | |  |
| male | FBgn0118841 | HE | E | FBgn0031873 | Gas41 | 1 | |  |
| male | FBgn0118854 | LE | E | --- | --- | 1 | |  |
| male | FBgn0118856 | HE | E | FBgn0000409 | Cyt-c-p | 1 | |  |
| male | FBgn0118863 | HE | E | FBgn0031948 | CG7149 | 1 | |  |
| male | FBgn0118880 | HE | E | FBgn0051922 | CG31922 | 1 | |  |
| male | FBgn0118894 | HE | E | FBgn0031256 | CG4164 | 1 | |  |
| male | FBgn0118901 | HE | E | FBgn0011570 | cpb | 1 | |  |
| male | FBgn0118917 | LE | E | FBgn0032343 | CG6201 | 1 | |  |
| male | FBgn0118924 | LE | E | FBgn0032330 | Samuel | 1 | |  |
| male | FBgn0118929 | LE | E | FBgn0031716 | CG14015 | 1 | |  |
| male | FBgn0118953 | HE | E | --- | --- | 1 | |  |
| male | FBgn0118968 | HE | E | FBgn0031676 | senju | 1 | |  |
| male | FBgn0118987 | HE | E | FBgn0031244 | CG11601 | 1 | |  |
| male | FBgn0118994 | LE | E | FBgn0032002 | CG8353 | 1 | |  |
| male | FBgn0119000 | LE | E | FBgn0032723 | ssp3 | 1 | |  |
| male | FBgn0119029 | HE | E | FBgn0020622 | Pi3K21B | 1 | |  |
| male | FBgn0119031 | LE | E | --- | --- | 1 | |  |
| male | FBgn0119033 | LE | E | FBgn0031961 | CG7102 | 1 | |  |
| male | FBgn0119043 | HE | E | FBgn0032601 | yellow-b | 1 | |  |
| male | FBgn0119044 | LE | E | FBgn0264695 | Mhc | 1 | |  |
| male | FBgn0119061 | HE | E | FBgn0031534 | Snx1 | 1 | |  |
| male | FBgn0119067 | HE | E | FBgn0032014 | CG7840 | 1 | |  |
| male | FBgn0119071 | HE | E | FBgn0032022 | CG14275 | 1 | |  |
| male | FBgn0119079 | HE | E | FBgn0032729 | CG10639 | 1 | |  |
| male | FBgn0119080 | LE | E | FBgn0015772 | Nak | 1 | |  |
| male | FBgn0119082 | LE | E | FBgn0032726 | CG10621 | 1 | |  |
| male | FBgn0119090 | HE | E | FBgn0031977 | baf | 1 | |  |
| male | FBgn0119101 | HE | E | FBgn0032640 | Sgt | 1 | |  |
| male | FBgn0119122 | LE | E | FBgn0000114 | aret | 1 | |  |
| male | FBgn0119124 | HE | E | --- | --- | 1 | |  |
| male | FBgn0119133 | HE | E | FBgn0032860 | CG15130 | 1 | |  |
| male | FBgn0119135 | HE | E | FBgn0032857 | CG10947 | 1 | |  |
| male | FBgn0119138 | HE | E | FBgn0019982 | Gs1l | 1 | |  |
| male | FBgn0119143 | LE | E | FBgn0031602 | CG15431 | 1 | |  |
| male | FBgn0119148 | LE | E | FBgn0259176 | bun | 1 | |  |
| male | FBgn0119150 | LE | E | --- | --- | 1 | |  |
| male | FBgn0119158 | HE | E | FBgn0265298 | SC35 | 1 | |  |
| male | FBgn0119160 | HE | E | FBgn0032261 | CG6094 | 1 | |  |
| male | FBgn0119166 | HE | E | --- | --- | 1 | |  |
| male | FBgn0119168 | HE | E | --- | --- | 1 | |  |
| male | FBgn0119185 | HE | E | FBgn0031630 | CG15629 | 1 | |  |
| male | FBgn0119188 | HE | E | FBgn0031590 | CG3702 | 1 | |  |
| male | FBgn0119195 | HE | E | FBgn0051975 | CG31975 | 1 | |  |
| male | FBgn0119199 | HE | E | FBgn0026787 | Nhe1 | 1 | |  |
| male | FBgn0119207 | HE | E | --- | --- | 1 | |  |
| male | FBgn0119221 | HE | E | FBgn0037251 | CG9804 | 1 | |  |
| male | FBgn0119228 | HE | E | FBgn0037818 | CG6465 | 1 | |  |
| male | FBgn0119244 | LE | E | --- | --- | 1 | |  |
| male | FBgn0119261 | LE | E | --- | --- | 1 | |  |
| male | FBgn0119271 | HE | E | FBgn0028691 | Rpn9 | 1 | |  |
| male | FBgn0119276 | LE | E | FBgn0032681 | CG10283 | 1 | |  |
| male | FBgn0119285 | HE | E | FBgn0019929 | Ser7 | 1 | |  |
| male | FBgn0119291 | LE | E | FBgn0052694 | CG32694 | 1 | |  |
| male | FBgn0119294 | LE | E | FBgn0263132 | Cht6 | 1 | |  |
| male | FBgn0119295 | LE | E | FBgn0263132 | Cht6 | 1 | |  |
| male | FBgn0119296 | LE | E | --- | --- | 1 | |  |
| male | FBgn0119300 | LE | E | FBgn0001083 | fw | 1 | |  |
| male | FBgn0119311 | HE | E | FBgn0028327 | l(1)G0320 | 1 | |  |
| male | FBgn0119326 | HE | E | FBgn0020369 | Rpt6 | 1 | |  |
| male | FBgn0119330 | HE | E | FBgn0030518 | CG11134 | 1 | |  |
| male | FBgn0119332 | HE | E | FBgn0030122 | CG16892 | 1 | |  |
| male | FBgn0119337 | LE/HE | E | --- | --- | 1 | |  |
| male | FBgn0119340 | HE | E | --- | --- | 1 | |  |
| male | FBgn0119355 | HE | E | --- | --- | 1 | |  |
| male | FBgn0119360 | LE | E | FBgn0026144 | CBP | 1 | |  |
| male | FBgn0119361 | LE | E | --- | --- | 1 | |  |
| male | FBgn0119366 | HE | E | FBgn0029950 | CG9657 | 1 | |  |
| male | FBgn0119371 | HE | E | FBgn0000618 | e(y)2 | 1 | |  |
| male | FBgn0119372 | LE | E | --- | --- | 1 | |  |
| male | FBgn0119373 | HE | E | FBgn0040899 | CG17776 | 1 | |  |
| male | FBgn0119374 | HE | E | --- | --- | 1 | |  |
| male | FBgn0119377 | HE | E | FBgn0029133 | REG | 1 | |  |
| male | FBgn0119398 | HE | E | FBgn0030345 | CG1847 | 1 | |  |
| male | FBgn0119399 | LE | E | FBgn0030520 | Pdcd4 | 1 | |  |
| male | FBgn0119401 | LE | E | FBgn0052703 | Erk7 | 1 | |  |
| male | FBgn0119405 | HE | E | FBgn0030801 | Rcp | 1 | |  |
| male | FBgn0119407 | HE | E | FBgn0000152 | Axs | 1 | |  |
| male | FBgn0119419 | HE | E | FBgn0030510 | CG12177 | 1 | |  |
| male | FBgn0119423 | HE | E | FBgn0024995 | CG2680 | 1 | |  |
| male | FBgn0119435 | HE | E | FBgn0030593 | CG9512 | 1 | |  |
| male | FBgn0119440 | HE | E | FBgn0030588 | CG9521 | 1 | |  |
| male | FBgn0119440 | HE | E | FBgn0030589 | CG9519 | 1 | |  |
| male | FBgn0119449 | LE | E | FBgn0283471 | wupA | 1 | |  |
| male | FBgn0119451 | LE | E | FBgn0260753 | Pdfr | 1 | |  |
| male | FBgn0119454 | HE | E | FBgn0001218 | Hsc70-3 | 1 | |  |
| male | FBgn0119457 | HE | E | FBgn0023510 | Rbcn-3B | 1 | |  |
| male | FBgn0119462 | HE | E | FBgn0040340 | TRAM | 1 | |  |
| male | FBgn0119496 | LE/HE | E | FBgn0083167 | Neb-cGP | 1 | |  |
| male | FBgn0119504 | HE | E | FBgn0030218 | CG1628 | 1 | |  |
| male | FBgn0119506 | LE | E | FBgn0030108 | Gr8a | 1 | |  |
| male | FBgn0119509 | HE | E | FBgn0030749 | AnxB11 | 1 | |  |
| male | FBgn0119510 | HE | E | FBgn0030746 | CG9981 | 1 | |  |
| male | FBgn0119525 | HE | E | FBgn0029783 | Sirt4 | 1 | |  |
| male | FBgn0119526 | HE | E | FBgn0039965 | CG13865 | 1 | |  |
| male | FBgn0119527 | LE | E | FBgn0265767 | zyd | 1 | |  |
| male | FBgn0119551 | LE | E | FBgn0030066 | CG1885 | 1 | |  |
| male | FBgn0119562 | HE | E | FBgn0031099 | CG17065 | 1 | |  |
| male | FBgn0119577 | HE | E | FBgn0031050 | Arp10 | 1 | |  |
| male | FBgn0119582 | HE | E | FBgn0029930 | CG12541 | 1 | |  |
| male | FBgn0119594 | LE | E | FBgn0029737 | CG6903 | 1 | |  |
| male | FBgn0119595 | HE | E | FBgn0024987 | ssx | 1 | |  |
| male | FBgn0119631 | HE | E | FBgn0030734 | CG9911 | 1 | |  |
| male | FBgn0119632 | HE | E | FBgn0052576 | CG32576 | 1 | |  |
| male | FBgn0119634 | HE | E | FBgn0028427 | Ilk | 1 | |  |
| male | FBgn0119650 | HE | E | FBgn0065035 | AlkB | 1 | |  |
| male | FBgn0119658 | HE | E | FBgn0030292 | CG11752 | 1 | |  |
| male | FBgn0119659 | HE | E | FBgn0030293 | CG1737 | 1 | |  |
| male | FBgn0119693 | HE | E | FBgn0015010 | Ag5r | 1 | |  |
| male | FBgn0119701 | HE | E | FBgn0001491 | l(1)10Bb | 1 | |  |
| male | FBgn0119722 | LE | E | FBgn0053181 | CG33181 | 1 | |  |
| male | FBgn0119739 | HE | E | --- | --- | 1 | |  |
| male | FBgn0119751 | HE | E | FBgn0003996 | w | 1 | |  |
| male | FBgn0119754 | LE | E | FBgn0000376 | crm | 1 | |  |
| male | FBgn0119802 | LE | E | --- | --- | 1 | |  |
| male | FBgn0119805 | LE | E | FBgn0031190 | CG12576 | 1 | |  |
| male | FBgn0119809 | HE | E | FBgn0030060 | CG2004 | 1 | |  |
| male | FBgn0119818 | HE | E | FBgn0030928 | CG15044 | 1 | |  |
| male | FBgn0119822 | LE | E | FBgn0030925 | Hayan | 1 | |  |
| male | FBgn0119823 | LE | E | --- | --- | 1 | |  |
| male | FBgn0119840 | LE | E | FBgn0030027 | CG1632 | 1 | |  |
| male | FBgn0119843 | HE | E | FBgn0031174 | CG1486 | 1 | |  |
| male | FBgn0119854 | HE | E | FBgn0001186 | Hex-A | 1 | |  |
| male | FBgn0119855 | HE | E | FBgn0030552 | mRpL38 | 1 | |  |
| male | FBgn0119877 | HE | E | FBgn0029665 | CG14270 | 1 | |  |
| male | FBgn0119879 | LE | E | FBgn0029662 | CG12206 | 1 | |  |
| male | FBgn0119889 | LE | E | FBgn0015519 | nAChRalpha3 | 1 | |  |
| male | FBgn0119930 | LE | E | FBgn0052732 | CG32732 | 1 | |  |
| male | FBgn0119932 | LE | E | --- | --- | 1 | |  |
| male | FBgn0119935 | LE | E | FBgn0040153 | l(1)G0469 | 1 | |  |
| male | FBgn0119936 | HE | E | FBgn0030092 | fh | 1 | |  |
| male | FBgn0119949 | HE | E | FBgn0030348 | CG10352 | 1 | |  |
| male | FBgn0119966 | LE | E | FBgn0028369 | kirre | 1 | |  |
| male | FBgn0120010 | HE | E | FBgn0030955 | CG6891 | 1 | |  |
| male | FBgn0120013 | HE | E | FBgn0014391 | sun | 1 | |  |
| male | FBgn0120023 | HE | E | FBgn0030711 | Rrp47 | 1 | |  |
| male | FBgn0120025 | HE | E | FBgn0033836 | CG18278 | 1 | |  |
| male | FBgn0120025 | HE | E | FBgn0260475 | CG30059 | 1 | |  |
| male | FBgn0120029 | HE | E | FBgn0026076 | UBL3 | 1 | |  |
| male | FBgn0120038 | HE | E | FBgn0030733 | UQCR-14 | 1 | |  |
| male | FBgn0120038 | HE | E | FBgn0039576 | UQCR-14L | 1 | |  |
| male | FBgn0120044 | LE | E | FBgn0259168 | mnb | 1 | |  |
| male | FBgn0120053 | LE | E | FBgn0020513 | ade5 | 1 | |  |
| male | FBgn0120067 | HE | E | FBgn0024986 | CG3719 | 1 | |  |
| male | FBgn0120076 | HE | E | FBgn0050195 | CG30195 | 1 | |  |
| male | FBgn0120080 | HE | E | FBgn0024983 | CG4293 | 1 | |  |
| male | FBgn0120083 | HE | E | FBgn0004143 | nullo | 1 | |  |
| male | FBgn0120092 | HE | E | FBgn0030234 | CG15211 | 1 | |  |
| male | FBgn0120101 | HE | E | --- | --- | 1 | |  |
| male | FBgn0120123 | HE | E | FBgn0030834 | CG8675 | 1 | |  |
| male | FBgn0120134 | HE | E | FBgn0030237 | CG15209 | 1 | |  |
| male | FBgn0120137 | LE | E | FBgn0032779 | CG16771 | 1 | |  |
| male | FBgn0120142 | LE | E | FBgn0039945 | CG17159 | 1 | |  |
| male | FBgn0120160 | HE | E | FBgn0037973 | CG18547 | 1 | |  |
| male | FBgn0120168 | HE | E | FBgn0029878 | Pat1 | 1 | |  |
| male | FBgn0120196 | HE | E | FBgn0030196 | Psf3 | 1 | |  |
| male | FBgn0120218 | HE | E | FBgn0040350 | CG3690 | 1 | |  |
| male | FBgn0120222 | HE | E | FBgn0030352 | sicily | 1 | |  |
| male | FBgn0120227 | LE | E | FBgn0040377 | Vha36-3 | 1 | |  |
| male | FBgn0120232 | HE | E | FBgn0020653 | Trxr-1 | 1 | |  |
| male | FBgn0120243 | HE | E | FBgn0030605 | ND-B18 | 1 | |  |
| male | FBgn0120246 | LE | E | FBgn0004110 | tin | 1 | |  |
| male | FBgn0120260 | HE | E | FBgn0040234 | c12.2 | 1 | |  |
| male | FBgn0120270 | HE | E | FBgn0030802 | DENR | 1 | |  |
| male | FBgn0120274 | LE | E | FBgn0030114 | CG17754 | 1 | |  |
| male | FBgn0120298 | LE | E | FBgn0283680 | IP3K2 | 1 | |  |
| male | FBgn0120308 | HE | E | FBgn0029723 | Proc-R | 1 | |  |
| male | FBgn0120358 | LE | E | FBgn0030723 | dpr18 | 1 | |  |
| male | FBgn0120361 | HE | E | FBgn0001105 | Gbeta13F | 1 | |  |
| male | FBgn0120367 | HE | E | FBgn0029067 | Dd | 1 | |  |
| male | FBgn0120381 | HE | E | FBgn0040235 | c12.1 | 1 | |  |
| male | FBgn0120383 | LE | E | FBgn0030484 | GstT4 | 1 | |  |
| male | FBgn0120395 | HE | E | --- | --- | 1 | |  |
| male | FBgn0120399 | HE | E | --- | --- | 1 | |  |
| male | FBgn0120407 | LE | E | FBgn0030558 | CG1461 | 1 | |  |
| male | FBgn0120427 | HE | E | --- | --- | 1 | |  |
| male | FBgn0120451 | LE | E | --- | --- | 1 | |  |
| male | FBgn0120469 | LE | E | --- | --- | 1 | |  |
| male | FBgn0120488 | LE | E | --- | --- | 1 | |  |
| male | FBgn0120489 | HE | E | FBgn0032236 | mRpS7 | 1 | |  |
| male | FBgn0120490 | HE | E | FBgn0250816 | AGO3 | 1 | |  |
| male | FBgn0120499 | LE | E | FBgn0032940 | Mondo | 1 | |  |
| male | FBgn0120501 | HE | E | FBgn0032119 | CG3769 | 1 | |  |
| male | FBgn0120511 | HE | E | --- | --- | 1 | |  |
| male | FBgn0120522 | LE | E | FBgn0032820 | fbp | 1 | |  |
| male | FBgn0120525 | HE | E | --- | --- | 1 | |  |
| male | FBgn0120532 | HE | E | FBgn0031817 | CG9531 | 1 | |  |
| male | FBgn0120535 | HE | E | FBgn0032906 | RPA2 | 1 | |  |
| male | FBgn0120539 | HE | E | FBgn0032390 | dgt2 | 1 | |  |
| male | FBgn0120578 | LE | E | FBgn0086673 | CG13272 | 1 | |  |
| male | FBgn0120582 | LE | E | FBgn0032613 | CG13283 | 1 | |  |
| male | FBgn0120587 | HE | E | FBgn0028949 | CG15254 | 1 | |  |
| male | FBgn0120588 | HE | E | --- | --- | 1 | |  |
| male | FBgn0120601 | LE | E | FBgn0259244 | CG42342 | 1 | |  |
| male | FBgn0120605 | LE | E | FBgn0003475 | spir | 1 | |  |
| male | FBgn0120607 | LE | E | FBgn0031611 | FIG4 | 1 | |  |
| male | FBgn0120608 | HE | E | FBgn0031619 | CG3355 | 1 | |  |
| male | FBgn0120616 | HE | E | FBgn0039265 | CG11790 | 1 | |  |
| male | FBgn0120622 | HE | E | FBgn0031913 | CG5958 | 1 | |  |
| male | FBgn0120633 | LE | E | FBgn0031897 | CG13784 | 1 | |  |
| male | FBgn0120634 | LE | E | FBgn0051211 | CG31211 | 1 | |  |
| male | FBgn0120658 | LE | E | FBgn0031560 | CG16713 | 1 | |  |
| male | FBgn0120662 | HE | E | FBgn0031558 | CG16704 | 1 | |  |
| male | FBgn0120674 | LE | E | FBgn0014396 | tim | 1 | |  |
| male | FBgn0120676 | LE | E | FBgn0260486 | Ziz | 1 | |  |
| male | FBgn0120680 | LE | E | FBgn0031299 | CG4629 | 1 | |  |
| male | FBgn0120684 | LE | E | --- | --- | 1 | |  |
| male | FBgn0120688 | HE | E | FBgn0032451 | spict | 1 | |  |
| male | FBgn0120694 | HE | E | FBgn0032924 | Nbr | 1 | |  |
| male | FBgn0120695 | HE | E | FBgn0028411 | Nxt1 | 1 | |  |
| male | FBgn0120696 | HE | E | FBgn0032922 | CG9249 | 1 | |  |
| male | FBgn0120707 | LE | E | FBgn0003209 | raw | 1 | |  |
| male | FBgn0120717 | LE | E | FBgn0263773 | fok | 1 | |  |
| male | FBgn0120719 | HE | E | FBgn0003978 | vls | 1 | |  |
| male | FBgn0120721 | HE | E | FBgn0031824 | CG9547 | 1 | |  |
| male | FBgn0120724 | HE | E | FBgn0031822 | CG9548 | 1 | |  |
| male | FBgn0120725 | LE/HE | E | FBgn0031821 | KFase | 1 | |  |
| male | FBgn0120756 | LE/HE | E | FBgn0031881 | MME1 | 1 | |  |
| male | FBgn0120771 | HE | E | FBgn0021761 | Nup154 | 1 | |  |
| male | FBgn0120793 | HE | E | FBgn0032945 | CG8665 | 1 | |  |
| male | FBgn0120801 | HE | E | FBgn0032116 | Mco1 | 1 | |  |
| male | FBgn0120805 | HE | E | FBgn0015000 | betaggt-I | 1 | |  |
| male | FBgn0120819 | LE/HE | E | FBgn0031877 | CG10399 | 1 | |  |
| male | FBgn0120826 | HE | E | FBgn0058439 | CG40439 | 1 | |  |
| male | FBgn0120840 | HE | E | FBgn0053303 | CG33303 | 1 | |  |
| male | FBgn0120845 | HE | E | FBgn0032222 | CG5037 | 1 | |  |
| male | FBgn0120863 | LE | E | FBgn0264442 | ab | 1 | |  |
| male | FBgn0120865 | LE | E | FBgn0040232 | cmet | 1 | |  |
| male | FBgn0120865 | LE | E | FBgn0040223 | cana | 1 | |  |
| male | FBgn0120880 | LE | E | FBgn0000056 | Adhr | 1 | |  |
| male | FBgn0120889 | HE | E | FBgn0003087 | pim | 1 | |  |
| male | FBgn0120897 | LE | E | FBgn0001321 | knk | 1 | |  |
| male | FBgn0120903 | HE | E | FBgn0032810 | CG13077 | 1 | |  |
| male | FBgn0120906 | HE | E | FBgn0028507 | CG3793 | 1 | |  |
| male | FBgn0120911 | HE | E | FBgn0000075 | amd | 1 | |  |
| male | FBgn0120923 | LE | E | FBgn0032772 | CG17350 | 1 | |  |
| male | FBgn0120925 | HE | E | FBgn0032781 | RtcB | 1 | |  |
| male | FBgn0120930 | HE | E | FBgn0038491 | CG5292 | 1 | |  |
| male | FBgn0120931 | HE | E | FBgn0032511 | ND-B22 | 1 | |  |
| male | FBgn0120936 | HE | E | FBgn0051852 | Tap42 | 1 | |  |
| male | FBgn0120940 | HE | E | FBgn0025724 | beta'COP | 1 | |  |
| male | FBgn0120956 | HE | E | FBgn0086443 | AsnRS | 1 | |  |
| male | FBgn0120959 | HE | E | FBgn0000422 | Ddc | 1 | |  |
| male | FBgn0120966 | LE | E | FBgn0032042 | CG13398 | 1 | |  |
| male | FBgn0120977 | HE | E | FBgn0032801 | CG10165 | 1 | |  |
| male | FBgn0120989 | HE | E | FBgn0005771 | noc | 1 | |  |
| male | FBgn0120991 | LE | E | FBgn0031693 | Cyp4ac1 | 1 | |  |
| male | FBgn0120991 | LE | E | FBgn0031694 | Cyp4ac2 | 1 | |  |
| male | FBgn0120991 | LE | E | FBgn0031695 | Cyp4ac3 | 1 | |  |
| male | FBgn0120992 | HE | E | FBgn0032205 | CG4957 | 1 | |  |
| male | FBgn0120994 | HE | E | FBgn0085396 | CG34367 | 1 | |  |
| male | FBgn0120997 | HE | E | FBgn0038631 | CG7695 | 1 | |  |
| male | FBgn0121008 | LE | E | FBgn0038511 | cysu | 1 | |  |
| male | FBgn0121011 | HE | E | FBgn0031702 | fusl | 1 | |  |
| male | FBgn0121012 | HE | E | FBgn0031700 | CG14022 | 1 | |  |
| male | FBgn0121029 | HE | E | FBgn0032198 | eEF1delta | 1 | |  |
| male | FBgn0121030 | LE | E | FBgn0038516 | P5cr-2 | 1 | |  |
| male | FBgn0121042 | HE | E | FBgn0039969 | Fis1 | 1 | |  |
| male | FBgn0121044 | HE | E | --- | --- | 1 | |  |
| male | FBgn0121046 | HE | E | --- | --- | 1 | |  |
| male | FBgn0121054 | HE | E | FBgn0264922 | smt3 | 1 | |  |
| male | FBgn0121061 | LE | E | FBgn0032299 | CG17127 | 1 | |  |
| male | FBgn0121066 | LE | E | FBgn0262599 | SmydA-3 | 1 | |  |
| male | FBgn0121082 | HE | E | FBgn0032230 | lft | 1 | |  |
| male | FBgn0121087 | HE | E | FBgn0067622 | LSm-4 | 1 | |  |
| male | FBgn0121088 | HE | E | FBgn0032247 | CG5188 | 1 | |  |
| male | FBgn0121089 | HE | E | FBgn0032240 | CG17768 | 1 | |  |
| male | FBgn0121098 | HE | E | FBgn0031217 | CG11377 | 1 | |  |
| male | FBgn0121103 | HE | E | FBgn0002566 | lt | 1 | |  |
| male | FBgn0121124 | LE | E | FBgn0032194 | CG4901 | 1 | |  |
| male | FBgn0121158 | HE | E | FBgn0261976 | Psf2 | 1 | |  |
| male | FBgn0121174 | LE | E | FBgn0015663 | Dot | 1 | |  |
| male | FBgn0121176 | HE | E | --- | --- | 1 | |  |
| male | FBgn0121177 | LE | E | FBgn0031564 | CG2816 | 1 | |  |
| male | FBgn0121179 | LE | E | FBgn0003386 | Shaw | 1 | |  |
| male | FBgn0121182 | LE | E | FBgn0024321 | NK7.1 | 1 | |  |
| male | FBgn0121184 | LE | E | FBgn0263846 | CG43707 | 1 | |  |
| male | FBgn0121201 | HE | E | FBgn0031902 | Wnt6 | 1 | |  |
| male | FBgn0121209 | HE | E | FBgn0031908 | CG5177 | 1 | |  |
| male | FBgn0121217 | LE | E | --- | --- | 1 | |  |
| male | FBgn0121224 | HE | E | FBgn0011638 | La | 1 | |  |
| male | FBgn0121228 | LE | E | --- | --- | 1 | |  |
| male | FBgn0121242 | HE | E | FBgn0028518 | CG18480 | 1 | |  |
| male | FBgn0121262 | LE | E | FBgn0031413 | CG9967 | 1 | |  |
| male | FBgn0121263 | HE | E | --- | --- | 1 | |  |
| male | FBgn0121280 | LE | E | FBgn0004367 | mei-41 | 1 | |  |
| male | FBgn0121284 | HE | E | --- | --- | 1 | |  |
| male | FBgn0121288 | LE/HE | E | FBgn0032382 | Mal-B2 | 1 | |  |
| male | FBgn0121295 | HE | E | FBgn0261266 | zuc | 1 | |  |
| male | FBgn0121300 | HE | E | FBgn0032149 | CG4036 | 1 | |  |
| male | FBgn0121312 | LE | E | FBgn0032836 | CG10680 | 1 | |  |
| male | FBgn0121315 | HE | E | --- | --- | 1 | |  |
| male | FBgn0121317 | HE | E | FBgn0031830 | COX5B | 1 | |  |
| male | FBgn0121320 | HE | E | FBgn0263916 | Ent2 | 1 | |  |
| male | FBgn0121325 | LE | E | FBgn0264556 | Gr39a | 1 | |  |
| male | FBgn0121331 | LE | E | FBgn0032219 | CG4995 | 1 | |  |
| male | FBgn0121332 | HE | E | FBgn0004106 | Cdk1 | 1 | |  |
| male | FBgn0121334 | HE | E | FBgn0032218 | CG5381 | 1 | |  |
| male | FBgn0121342 | LE | E | FBgn0052986 | CG32986 | 1 | |  |
| male | FBgn0121356 | HE | E | FBgn0003087 | pim | 1 | |  |
| male | FBgn0121373 | HE | E | FBgn0031312 | Tango14 | 1 | |  |
| male | FBgn0121380 | LE | E | FBgn0002922 | nau | 1 | |  |
| male | FBgn0121409 | LE | E | FBgn0013756 | Mtor | 1 | |  |
| male | FBgn0121414 | LE | E | FBgn0039641 | CG14511 | 1 | |  |
| male | FBgn0121426 | HE | E | FBgn0036501 | CG7272 | 1 | |  |
| male | FBgn0121429 | LE | E | --- | --- | 1 | |  |
| male | FBgn0121434 | HE | E | FBgn0032810 | CG13077 | 1 | |  |
| male | FBgn0121445 | LE | E | FBgn0010015 | CanA1 | 1 | |  |
| male | FBgn0121446 | HE | E | FBgn0016123 | Alp4 | 1 | |  |
| male | FBgn0121455 | HE | E | FBgn0039828 | CG1542 | 1 | |  |
| male | FBgn0121457 | HE | E | FBgn0010803 | TrpRS | 1 | |  |
| male | FBgn0121464 | LE | E | FBgn0039430 | CG5455 | 1 | |  |
| male | FBgn0121465 | HE | E | FBgn0027583 | CG7601 | 1 | |  |
| male | FBgn0121466 | HE | E | FBgn0039687 | CG7593 | 1 | |  |
| male | FBgn0121474 | LE | E | FBgn0024556 | EfTuM | 1 | |  |
| male | FBgn0121476 | HE | E | FBgn0015622 | Cnx99A | 1 | |  |
| male | FBgn0121484 | LE | E | FBgn0039647 | CG14509 | 1 | |  |
| male | FBgn0121491 | HE | E | FBgn0034480 | CG16898 | 1 | |  |
| male | FBgn0121491 | HE | E | FBgn0053301 | CG33301 | 1 | |  |
| male | FBgn0121519 | HE | E | FBgn0039768 | CG15533 | 1 | |  |
| male | FBgn0121523 | HE | E | FBgn0051469 | CG31469 | 1 | |  |
| male | FBgn0121527 | LE | E | FBgn0037536 | CG2698 | 1 | |  |
| male | FBgn0121529 | LE | E | FBgn0039241 | CG11089 | 1 | |  |
| male | FBgn0121530 | HE | E | FBgn0039768 | CG15533 | 1 | |  |
| male | FBgn0121541 | HE | E | FBgn0039769 | CG15534 | 1 | |  |
| male | FBgn0121549 | HE | E | FBgn0039453 | CG6403 | 1 | |  |
| male | FBgn0121556 | HE | E | FBgn0037756 | CG8507 | 1 | |  |
| male | FBgn0121557 | HE | E | FBgn0037756 | CG8507 | 1 | |  |
| male | FBgn0121561 | LE | E | FBgn0266756 | btsz | 1 | |  |
| male | FBgn0121564 | LE | E | FBgn0038256 | CG7530 | 1 | |  |
| male | FBgn0121572 | LE | E | FBgn0039806 | CG15545 | 1 | |  |
| male | FBgn0121595 | HE | E | FBgn0039877 | CG2118 | 1 | |  |
| male | FBgn0121599 | HE | E | FBgn0039873 | Smvt | 1 | |  |
| male | FBgn0121600 | HE | E | FBgn0017448 | CG2187 | 1 | |  |
| male | FBgn0121600 | HE | E | FBgn0039872 | salt | 1 | |  |
| male | FBgn0121601 | HE | E | FBgn0261479 | nero | 1 | |  |
| male | FBgn0121607 | HE | E | --- | --- | 1 | |  |
| male | FBgn0121610 | LE | E | --- | --- | 1 | |  |
| male | FBgn0121620 | LE | E | --- | --- | 1 | |  |
| male | FBgn0121622 | LE | E | --- | --- | 1 | |  |
| male | FBgn0121635 | HE | E | FBgn0037934 | CG6830 | 1 | |  |
| male | FBgn0121635 | LE | E | FBgn0027655 | htt | 1 | |  |
| male | FBgn0121641 | HE | E | FBgn0040575 | CG15922 | 1 | |  |
| male | FBgn0121653 | HE | E | FBgn0037770 | Art4 | 1 | |  |
| male | FBgn0121694 | LE | E | FBgn0039804 | CG15544 | 1 | |  |
| male | FBgn0121695 | LE | E | FBgn0003720 | tll | 1 | |  |
| male | FBgn0121705 | HE | E | --- | --- | 1 | |  |
| male | FBgn0121706 | HE | E | FBgn0038262 | CG14857 | 1 | |  |
| male | FBgn0121708 | HE | E | FBgn0086605 | CG9853 | 1 | |  |
| male | FBgn0121719 | HE | E | FBgn0039459 | IntS12 | 1 | |  |
| male | FBgn0121730 | HE | E | FBgn0014141 | cher | 1 | |  |
| male | FBgn0121732 | HE | E | --- | --- | 1 | |  |
| male | FBgn0121733 | HE | E | FBgn0265189 | sud1 | 1 | |  |
| male | FBgn0121739 | HE | E | FBgn0037537 | CG2767 | 1 | |  |
| male | FBgn0121748 | LE | E | FBgn0038180 | Cht5 | 1 | |  |
| male | FBgn0121750 | HE | E | FBgn0259721 | CG42375 | 1 | |  |
| male | FBgn0121761 | LE | E | FBgn0046875 | Obp83g | 1 | |  |
| male | FBgn0121763 | LE | E | FBgn0026077 | Gasp | 1 | |  |
| male | FBgn0121768 | HE | E | FBgn0037755 | CG12945 | 1 | |  |
| male | FBgn0121773 | HE | E | FBgn0014869 | Pglym78 | 1 | |  |
| male | FBgn0121781 | HE | E | FBgn0062442 | Cisd2 | 1 | |  |
| male | FBgn0121785 | LE | E | FBgn0004369 | Ptp99A | 1 | |  |
| male | FBgn0121786 | HE | E | FBgn0027785 | NP15.6 | 1 | |  |
| male | FBgn0121794 | HE | E | FBgn0039686 | CG15506 | 1 | |  |
| male | FBgn0121797 | HE | E | FBgn0042213 | CG18731 | 1 | |  |
| male | FBgn0121799 | LE | E | FBgn0039689 | CIA30 | 1 | |  |
| male | FBgn0121800 | HE | E | FBgn0039690 | CG1969 | 1 | |  |
| male | FBgn0121803 | HE | E | FBgn0260990 | yata | 1 | |  |
| male | FBgn0121808 | LE | E | FBgn0262112 | sro | 1 | |  |
| male | FBgn0121813 | HE | E | FBgn0024330 | MED6 | 1 | |  |
| male | FBgn0121834 | HE | E | FBgn0037580 | DppIII | 1 | |  |
| male | FBgn0121842 | LE | E | FBgn0037581 | CG7352 | 1 | |  |
| male | FBgn0121855 | HE | E | FBgn0036967 | CG6597 | 1 | |  |
| male | FBgn0121857 | HE | E | FBgn0250791 | alphaSnap | 1 | |  |
| male | FBgn0121871 | HE | E | FBgn0036878 | Cpr76Ba | 1 | |  |
| male | FBgn0121876 | HE | E | FBgn0036875 | CG9449 | 1 | |  |
| male | FBgn0121878 | LE | E | --- | --- | 1 | |  |
| male | FBgn0121885 | LE | E | --- | --- | 1 | |  |
| male | FBgn0121886 | LE | E | --- | --- | 1 | |  |
| male | FBgn0121890 | LE/HE | E | --- | --- | 1 | |  |
| male | FBgn0121893 | HE | E | FBgn0036862 | Gbs-76A | 1 | |  |
| male | FBgn0121894 | HE | E | FBgn0036857 | CG9629 | 1 | |  |
| male | FBgn0121895 | HE | E | FBgn0038421 | CG17931 | 1 | |  |
| male | FBgn0121898 | HE | E | FBgn0026630 | nes | 1 | |  |
| male | FBgn0121900 | HE | E | FBgn0036351 | CG14107 | 1 | |  |
| male | FBgn0121909 | HE | E | --- | --- | 1 | |  |
| male | FBgn0121912 | HE | E | FBgn0037146 | CG7470 | 1 | |  |
| male | FBgn0121914 | LE | E | FBgn0004514 | Oct-TyrR | 1 | |  |
| male | FBgn0121924 | HE | E | FBgn0052195 | CG32195 | 1 | |  |
| male | FBgn0121930 | HE | E | FBgn0036790 | AstC-R1 | 1 | |  |
| male | FBgn0121943 | HE | E | FBgn0036756 | cln3 | 1 | |  |
| male | FBgn0121945 | HE | E | FBgn0052191 | CG32191 | 1 | |  |
| male | FBgn0121956 | HE | E | FBgn0036335 | mRpL20 | 1 | |  |
| male | FBgn0121971 | LE | E | FBgn0260945 | Atg1 | 1 | |  |
| male | FBgn0121972 | LE | E | --- | --- | 1 | |  |
| male | FBgn0121974 | HE | E | FBgn0036920 | CG8004 | 1 | |  |
| male | FBgn0121986 | LE | E | FBgn0026160 | tna | 1 | |  |
| male | FBgn0121993 | HE | E | FBgn0035743 | CG15829 | 1 | |  |
| male | FBgn0122014 | LE | E | --- | --- | 1 | |  |
| male | FBgn0122017 | HE | E | FBgn0036501 | CG7272 | 1 | |  |
| male | FBgn0122020 | HE | E | FBgn0000565 | Eip71CD | 1 | |  |
| male | FBgn0122025 | HE | E | FBgn0038294 | Mf | 1 | |  |
| male | FBgn0122028 | HE | E | FBgn0036998 | CG5969 | 1 | |  |
| male | FBgn0122029 | HE | E | FBgn0036999 | isoQC | 1 | |  |
| male | FBgn0122046 | LE | E | FBgn0035392 | CG1271 | 1 | |  |
| male | FBgn0122053 | LE | E | FBgn0052846 | CG32846 | 1 | |  |
| male | FBgn0122063 | HE | E | FBgn0037011 | CG4858 | 1 | |  |
| male | FBgn0122073 | HE | E | FBgn0036820 | CG6852 | 1 | |  |
| male | FBgn0122075 | HE | E | FBgn0014075 | Ugt | 1 | |  |
| male | FBgn0122088 | LE | E | FBgn0036849 | CG14079 | 1 | |  |
| male | FBgn0122091 | HE | E | FBgn0039296 | CG10420 | 1 | |  |
| male | FBgn0122101 | LE | E | FBgn0005386 | ash1 | 1 | |  |
| male | FBgn0122112 | HE | E | FBgn0036136 | Ufd1-like | 1 | |  |
| male | FBgn0122116 | HE | E | FBgn0003462 | Sod | 1 | |  |
| male | FBgn0122132 | HE | E | FBgn0036909 | CG15881 | 1 | |  |
| male | FBgn0122135 | HE | E | FBgn0037345 | rev7 | 1 | |  |
| male | FBgn0122145 | HE | E | FBgn0283535 | Vha26 | 1 | |  |
| male | FBgn0122148 | HE | E | FBgn0015075 | Ddx1 | 1 | |  |
| male | FBgn0122151 | LE | E | FBgn0036361 | CG10154 | 1 | |  |
| male | FBgn0122155 | HE | E | FBgn0052121 | CG32121 | 1 | |  |
| male | FBgn0122156 | HE | E | FBgn0051450 | mRpS18A | 1 | |  |
| male | FBgn0122157 | HE | E | FBgn0036356 | CG10222 | 1 | |  |
| male | FBgn0122164 | HE | E | FBgn0052407 | CG32407 | 1 | |  |
| male | FBgn0122167 | LE | E | FBgn0051453 | pch2 | 1 | |  |
| male | FBgn0122173 | LE | E | FBgn0045477 | Gr64c | 1 | |  |
| male | FBgn0122174 | LE | E | FBgn0045476 | Gr64e | 1 | |  |
| male | FBgn0122188 | LE | E | FBgn0262524 | ver | 1 | |  |
| male | FBgn0122190 | HE | E | FBgn0036300 | CG10688 | 1 | |  |
| male | FBgn0122201 | HE | E | FBgn0052112 | CG32112 | 1 | |  |
| male | FBgn0122204 | HE | E | FBgn0037102 | CRIF | 1 | |  |
| male | FBgn0122214 | HE | E | FBgn0052554 | CG32554 | 1 | |  |
| male | FBgn0122223 | HE | E | FBgn0037589 | Obp85a | 1 | |  |
| male | FBgn0122228 | LE | E | FBgn0036433 | CG9628 | 1 | |  |
| male | FBgn0122234 | HE | E | --- | --- | 1 | |  |
| male | FBgn0122238 | HE | E | FBgn0010348 | Arf79F | 1 | |  |
| male | FBgn0122250 | HE | E | FBgn0036550 | CG17026 | 1 | |  |
| male | FBgn0122251 | HE | E | FBgn0036551 | CG17029 | 1 | |  |
| male | FBgn0122252 | HE | E | FBgn0036553 | CG17027 | 1 | |  |
| male | FBgn0122257 | HE | E | FBgn0040634 | CG4186 | 1 | |  |
| male | FBgn0122264 | HE | E | FBgn0263911 | COX8 | 1 | |  |
| male | FBgn0122271 | LE | E | FBgn0036354 | Poc1 | 1 | |  |
| male | FBgn0122285 | HE | E | FBgn0037182 | ArfGAP3 | 1 | |  |
| male | FBgn0122289 | HE | E | FBgn0039312 | CG10514 | 1 | |  |
| male | FBgn0122296 | HE | E | FBgn0031523 | CG15408 | 1 | |  |
| male | FBgn0122300 | HE | E | FBgn0039308 | CG11889 | 1 | |  |
| male | FBgn0122300 | HE | E | FBgn0039309 | CG11889 | 1 | |  |
| male | FBgn0122300 | HE | E | FBgn0039310 | CG11889 | 1 | |  |
| male | FBgn0122300 | HE | E | FBgn0039311 | CG11889 | 1 | |  |
| male | FBgn0122300 | HE | E | FBgn0039309 | CG11891 | 1 | |  |
| male | FBgn0122300 | HE | E | FBgn0039310 | CG11878 | 1 | |  |
| male | FBgn0122311 | HE | E | FBgn0039311 | CG10513 | 1 | |  |
| male | FBgn0122322 | HE | E | FBgn0267975 | vib | 1 | |  |
| male | FBgn0122326 | LE | E | --- | --- | 1 | |  |
| male | FBgn0122336 | HE | E | FBgn0036184 | PCID2 | 1 | |  |
| male | FBgn0122340 | HE | E | FBgn0015828 | TfIIEalpha | 1 | |  |
| male | FBgn0122363 | HE | E | FBgn0035788 | CG8541 | 1 | |  |
| male | FBgn0122418 | LE | E | FBgn0037046 | CG10581 | 1 | |  |
| male | FBgn0122431 | HE | E | FBgn0037883 | CG14701 | 1 | |  |
| male | FBgn0122446 | HE | E | FBgn0028427 | Ilk | 1 | |  |
| male | FBgn0122447 | HE | E | FBgn0004865 | Eip78C | 1 | |  |
| male | FBgn0122463 | LE | E | --- | --- | 1 | |  |
| male | FBgn0122466 | LE | E | --- | --- | 1 | |  |
| male | FBgn0122470 | HE | E | FBgn0035300 | CG1139 | 1 | |  |
| male | FBgn0122477 | HE | E | FBgn0015359 | CG2034 | 1 | |  |
| male | FBgn0122484 | LE | E | FBgn0036696 | CG14057 | 1 | |  |
| male | FBgn0122486 | HE | E | FBgn0263106 | DnaJ-1 | 1 | |  |
| male | FBgn0122496 | LE | E | FBgn0035663 | CG6462 | 1 | |  |
| male | FBgn0122508 | HE | E | FBgn0035673 | CG6602 | 1 | |  |
| male | FBgn0122524 | LE | E | FBgn0052264 | CG32264 | 1 | |  |
| male | FBgn0122529 | HE | E | FBgn0035875 | Cpr66Cb | 1 | |  |
| male | FBgn0122549 | HE | E | FBgn0261381 | mtTFB1 | 1 | |  |
| male | FBgn0122554 | LE | E | FBgn0003499 | sr | 1 | |  |
| male | FBgn0122574 | HE | E | FBgn0052447 | CG32447 | 1 | |  |
| male | FBgn0122576 | HE | E | FBgn0022943 | Cbp20 | 1 | |  |
| male | FBgn0122581 | HE | E | FBgn0026418 | Hsc70Cb | 1 | |  |
| male | FBgn0122583 | LE | E | FBgn0267795 | Frl | 1 | |  |
| male | FBgn0122584 | LE | E | FBgn0013563 | Pex1 | 1 | |  |
| male | FBgn0122591 | HE | E | FBgn0036947 | obst-F | 1 | |  |
| male | FBgn0122602 | HE | E | FBgn0036684 | CG3764 | 1 | |  |
| male | FBgn0122607 | LE | E | --- | --- | 1 | |  |
| male | FBgn0122612 | LE | E | FBgn0023129 | aay | 1 | |  |
| male | FBgn0122623 | LE | E | FBgn0036007 | path | 1 | |  |
| male | FBgn0122632 | LE | E | --- | --- | 1 | |  |
| male | FBgn0122641 | LE | E | FBgn0035708 | CG8398 | 1 | |  |
| male | FBgn0122654 | LE | E | FBgn0052296 | Mrtf | 1 | |  |
| male | FBgn0122670 | HE | E | FBgn0040239 | bc10 | 1 | |  |
| male | FBgn0122673 | HE | E | FBgn0035534 | mRpS6 | 1 | |  |
| male | FBgn0122680 | HE | E | FBgn0043458 | CG12084 | 1 | |  |
| male | FBgn0122681 | LE | E | FBgn0020248 | stet | 1 | |  |
| male | FBgn0122693 | HE | E | FBgn0010786 | l(3)02640 | 1 | |  |
| male | FBgn0122694 | HE | E | FBgn0036665 | CG13024 | 1 | |  |
| male | FBgn0122702 | LE | E | FBgn0063485 | Lasp | 1 | |  |
| male | FBgn0122703 | LE | E | FBgn0264605 | CG43954 | 1 | |  |
| male | FBgn0122704 | HE | E | FBgn0040512 | zetaCOP | 1 | |  |
| male | FBgn0122707 | HE | E | FBgn0040298 | Myt1 | 1 | |  |
| male | FBgn0122712 | HE | E | FBgn0266124 | ghi | 1 | |  |
| male | FBgn0122728 | HE | E | FBgn0047135 | CG32276 | 1 | |  |
| male | FBgn0122738 | LE | E | FBgn0035903 | CG6765 | 1 | |  |
| male | FBgn0122746 | HE | E | --- | --- | 1 | |  |
| male | FBgn0122747 | HE | E | FBgn0016031 | lama | 1 | |  |
| male | FBgn0122755 | LE | E | FBgn0040843 | CG15213 | 1 | |  |
| male | FBgn0122760 | LE | E | FBgn0035844 | CG13676 | 1 | |  |
| male | FBgn0122763 | HE | E | FBgn0042112 | mRpL36 | 1 | |  |
| male | FBgn0122765 | HE | E | FBgn0037288 | CG14661 | 1 | |  |
| male | FBgn0122769 | LE | E | FBgn0035290 | dsb | 1 | |  |
| male | FBgn0122770 | HE | E | FBgn0000109 | Aprt | 1 | |  |
| male | FBgn0122782 | HE | E | FBgn0036259 | CG9760 | 1 | |  |
| male | FBgn0122788 | HE | E | FBgn0037913 | fabp | 1 | |  |
| male | FBgn0122816 | LE | E | FBgn0036746 | Crtc | 1 | |  |
| male | FBgn0122817 | LE | E | --- | --- | 1 | |  |
| male | FBgn0122823 | LE | E | --- | --- | 1 | |  |
| male | FBgn0122827 | LE | E | FBgn0036735 | Edc3 | 1 | |  |
| male | FBgn0122831 | LE | E | FBgn0014388 | sty | 1 | |  |
| male | FBgn0122842 | HE | E | FBgn0029117 | Surf1 | 1 | |  |
| male | FBgn0122858 | HE | E | FBgn0035734 | CG14823 | 1 | |  |
| male | FBgn0122870 | LE | E | FBgn0052105 | Lmx1a | 1 | |  |
| male | FBgn0122883 | LE | E | --- | --- | 1 | |  |
| male | FBgn0122886 | HE | E | FBgn0035147 | Gale | 1 | |  |
| male | FBgn0122892 | HE | E | FBgn0260755 | CG42553 | 1 | |  |
| male | FBgn0122904 | HE | E | FBgn0036046 | Ilp2 | 1 | |  |
| male | FBgn0122910 | HE | E | FBgn0053965 | CG33965 | 1 | |  |
| male | FBgn0122918 | LE | E | FBgn0052333 | CG32333 | 1 | |  |
| male | FBgn0122927 | HE | E | FBgn0037999 | CG4860 | 1 | |  |
| male | FBgn0122934 | HE | E | --- | --- | 1 | |  |
| male | FBgn0122935 | HE | E | FBgn0260645 | CG42537 | 1 | |  |
| male | FBgn0122935 | HE | E | FBgn0261630 | CG42713 | 1 | |  |
| male | FBgn0122936 | HE | E | --- | --- | 1 | |  |
| male | FBgn0122944 | LE | E | FBgn0035131 | mthl9 | 1 | |  |
| male | FBgn0122948 | LE | E | FBgn0020386 | Pdk1 | 1 | |  |
| male | FBgn0122951 | HE | E | --- | --- | 1 | |  |
| male | FBgn0122956 | LE | E | --- | --- | 1 | |  |
| male | FBgn0122959 | LE | E | FBgn0264272 | mwh | 1 | |  |
| male | FBgn0122960 | LE | E | FBgn0037994 | CG4810 | 1 | |  |
| male | FBgn0122978 | LE | E | FBgn0035761 | RhoGEF4 | 1 | |  |
| male | FBgn0123007 | HE | E | FBgn0261952 | srw | 1 | |  |
| male | FBgn0123009 | HE | E | FBgn0035520 | CG11586 | 1 | |  |
| male | FBgn0123011 | HE | E | FBgn0035515 | CG14997 | 1 | |  |
| male | FBgn0123032 | LE | E | FBgn0037040 | CG12983 | 1 | |  |
| male | FBgn0123042 | HE | E | FBgn0047038 | ND-13B | 1 | |  |
| male | FBgn0123045 | HE | E | FBgn0035960 | CG4942 | 1 | |  |
| male | FBgn0123057 | LE | E | FBgn0004569 | aos | 1 | |  |
| male | FBgn0123066 | HE | E | FBgn0036621 | roq | 1 | |  |
| male | FBgn0123072 | LE/HE | E | FBgn0040795 | CG13038 | 1 | |  |
| male | FBgn0123076 | HE | E | --- | --- | 1 | |  |
| male | FBgn0123098 | LE | E | FBgn0036577 | CG13073 | 1 | |  |
| male | FBgn0123100 | LE | E | FBgn0036576 | CG5151 | 1 | |  |
| male | FBgn0123106 | HE | E | FBgn0005626 | ple | 1 | |  |
| male | FBgn0123107 | HE | E | FBgn0035710 | SP1173 | 1 | |  |
| male | FBgn0123110 | HE | E | FBgn0035968 | Slc45-1 | 1 | |  |
| male | FBgn0123110 | HE | E | FBgn0267429 | CG45782 | 1 | |  |
| male | FBgn0123122 | LE | E | FBgn0020643 | Lcp65Ab2 | 1 | |  |
| male | FBgn0123122 | LE | E | FBgn0020644 | Lcp65Ab1 | 1 | |  |
| male | FBgn0123133 | LE | E | FBgn0025592 | Gk1 | 1 | |  |
| male | FBgn0123162 | HE | E | FBgn0015805 | HDAC1 | 1 | |  |
| male | FBgn0123185 | LE | E | --- | --- | 1 | |  |
| male | FBgn0123189 | HE | E | FBgn0035272 | mRpL46 | 1 | |  |
| male | FBgn0123194 | LE | E | FBgn0022702 | Cht2 | 1 | |  |
| male | FBgn0123204 | HE | E | FBgn0035103 | Vdup1 | 1 | |  |
| male | FBgn0123206 | HE | E | FBgn0035120 | wac | 1 | |  |
| male | FBgn0123207 | HE | E | FBgn0035194 | Psf1 | 1 | |  |
| male | FBgn0123208 | HE | E | FBgn0035122 | mRpL17 | 1 | |  |
| male | FBgn0123229 | LE | E | FBgn0035343 | CG16762 | 1 | |  |
| male | FBgn0123232 | LE | E | FBgn0035266 | Gk2 | 1 | |  |
| male | FBgn0123236 | HE | E | FBgn0035271 | CG2021 | 1 | |  |
| male | FBgn0123237 | HE | E | FBgn0035469 | CG14977 | 1 | |  |
| male | FBgn0123262 | HE | E | FBgn0035570 | CG13712 | 1 | |  |
| male | FBgn0123263 | HE | E | FBgn0035569 | CG15876 | 1 | |  |
| male | FBgn0123272 | LE | E | FBgn0014073 | Tie | 1 | |  |
| male | FBgn0123282 | LE | E | --- | --- | 1 | |  |
| male | FBgn0123288 | LE | E | FBgn0035397 | CG11486 | 1 | |  |
| male | FBgn0123302 | HE | E | FBgn0061492 | loj | 1 | |  |
| male | FBgn0123321 | HE | E | FBgn0038542 | TyrR | 1 | |  |
| male | FBgn0123330 | HE | E | FBgn0263606 | Hsc20 | 1 | |  |
| male | FBgn0123338 | HE | E | --- | --- | 1 | |  |
| male | FBgn0123342 | HE | E | FBgn0036598 | CG4982 | 1 | |  |
| male | FBgn0123347 | HE | E | --- | --- | 1 | |  |
| male | FBgn0123353 | HE | E | FBgn0036612 | CG4998 | 1 | |  |
| male | FBgn0123357 | HE | E | FBgn0283681 | Tcs3 | 1 | |  |
| male | FBgn0123358 | LE | E | FBgn0036617 | Cpr72Ea | 1 | |  |
| male | FBgn0123360 | HE | E | FBgn0036620 | CG4842 | 1 | |  |
| male | FBgn0123360 | HE | E | FBgn0042137 | CG18814 | 1 | |  |
| male | FBgn0123378 | HE | E | FBgn0035770 | pst | 1 | |  |
| male | FBgn0123417 | HE | E | FBgn0040305 | MTF-1 | 1 | |  |
| male | FBgn0123420 | HE | E | FBgn0038829 | CG17271 | 1 | |  |
| male | FBgn0123421 | LE | E | FBgn0036690 | Ilp8 | 1 | |  |
| male | FBgn0123437 | LE | E | --- | --- | 1 | |  |
| male | FBgn0123444 | LE | E | FBgn0035866 | Arl5 | 1 | |  |
| male | FBgn0123449 | HE | E | --- | --- | 1 | |  |
| male | FBgn0123451 | LE | E | FBgn0035855 | CG7366 | 1 | |  |
| male | FBgn0123465 | HE | E | FBgn0035102 | CG7049 | 1 | |  |
| male | FBgn0123475 | LE | E | FBgn0028887 | CG3491 | 1 | |  |
| male | FBgn0123483 | HE | E | FBgn0028429 | I-2 | 1 | |  |
| male | FBgn0123487 | HE | E | FBgn0044049 | Ilp4 | 1 | |  |
| male | FBgn0123489 | LE | E | FBgn0036044 | Zasp67 | 1 | |  |
| male | FBgn0123495 | HE | E | FBgn0052251 | Claspin | 1 | |  |
| male | FBgn0123497 | HE | E | FBgn0052250 | PMP34 | 1 | |  |
| male | FBgn0123508 | HE | E | FBgn0035157 | CG13894 | 1 | |  |
| male | FBgn0123522 | LE | E | --- | --- | 1 | |  |
| male | FBgn0123524 | HE | E | FBgn0026404 | Dronc | 1 | |  |
| male | FBgn0123526 | HE | E | FBgn0263251 | vnc | 1 | |  |
| male | FBgn0123532 | HE | E | FBgn0036289 | CG10657 | 1 | |  |
| male | FBgn0123538 | LE | E | FBgn0052103 | CG32103 | 1 | |  |
| male | FBgn0123540 | LE | E | FBgn0038735 | CG4662 | 1 | |  |
| male | FBgn0123547 | HE | E | FBgn0036272 | CG4300 | 1 | |  |
| male | FBgn0123561 | HE | E | FBgn0015298 | Srp19 | 1 | |  |
| male | FBgn0123564 | LE | E | FBgn0035767 | CG8596 | 1 | |  |
| male | FBgn0123574 | HE | E | FBgn0261445 | sgl | 1 | |  |
| male | FBgn0123577 | LE | E | FBgn0041194 | Prat2 | 1 | |  |
| male | FBgn0123594 | LE | E | FBgn0036741 | CG7510 | 1 | |  |
| male | FBgn0123597 | HE | E | FBgn0036745 | CG7484 | 1 | |  |
| male | FBgn0123609 | LE | E | FBgn0035928 | CG13310 | 1 | |  |
| male | FBgn0123610 | HE | E | FBgn0035926 | CG5804 | 1 | |  |
| male | FBgn0123611 | HE | E | --- | --- | 1 | |  |
| male | FBgn0123612 | LE | E | FBgn0003149 | Prm | 1 | |  |
| male | FBgn0123626 | HE | E | FBgn0035896 | CG6983 | 1 | |  |
| male | FBgn0123635 | LE | E | FBgn0262624 | Tmhs | 1 | |  |
| male | FBgn0123658 | HE | E | FBgn0260856 | Membrin | 1 | |  |
| male | FBgn0123664 | HE | E | --- | --- | 1 | |  |
| male | FBgn0123665 | HE | E | FBgn0035902 | CG6683 | 1 | |  |
| male | FBgn0123667 | LE | E | FBgn0036264 | CG11529 | 1 | |  |
| male | FBgn0123674 | HE | E | FBgn0001233 | Hsp83 | 1 | |  |
| male | FBgn0123676 | HE | E | FBgn0035416 | gry | 1 | |  |
| male | FBgn0123687 | HE | E | FBgn0044419 | Pmi | 1 | |  |
| male | FBgn0123690 | LE | E | FBgn0000414 | Dab | 1 | |  |
| male | FBgn0123691 | LE | E | --- | --- | 1 | |  |
| male | FBgn0123692 | HE | E | FBgn0036659 | CG9701 | 1 | |  |
| male | FBgn0123695 | HE | E | FBgn0036661 | CG9705 | 1 | |  |
| male | FBgn0123704 | LE | E | FBgn0035142 | Hipk | 1 | |  |
| male | FBgn0123709 | LE | E | FBgn0035217 | FucTD | 1 | |  |
| male | FBgn0123713 | LE | E | FBgn0035621 | CG10591 | 1 | |  |
| male | FBgn0123717 | HE | E | FBgn0029118 | Sucb | 1 | |  |
| male | FBgn0123720 | HE | E | --- | --- | 1 | |  |
| male | FBgn0123722 | HE | E | FBgn0035610 | Lkr | 1 | |  |
| male | FBgn0123750 | HE | E | FBgn0001258 | ImpL3 | 1 | |  |
| male | FBgn0123778 | LE | E | FBgn0267796 | CG46121 | 1 | |  |
| male | FBgn0123783 | LE | E | FBgn0051431 | CG31431 | 1 | |  |
| male | FBgn0123785 | LE | E | FBgn0053696 | CNMaR | 1 | |  |
| male | FBgn0123792 | LE | E | FBgn0035986 | CG4022 | 1 | |  |
| male | FBgn0123793 | LE | E | FBgn0036676 | CG13028 | 1 | |  |
| male | FBgn0123809 | LE | E | FBgn0260393 | CG17147 | 1 | |  |
| male | FBgn0123813 | LE | E | FBgn0265959 | rdgC | 1 | |  |
| male | FBgn0123816 | LE | E | FBgn0051465 | CG31465 | 1 | |  |
| male | FBgn0123827 | HE | E | FBgn0260935 | Vps15 | 1 | |  |
| male | FBgn0123836 | HE | E | FBgn0052446 | Atox1 | 1 | |  |
| male | FBgn0123838 | LE | E | FBgn0015625 | CycB3 | 1 | |  |
| male | FBgn0123840 | HE | E | FBgn0037117 | CG11248 | 1 | |  |
| male | FBgn0123846 | HE | E | FBgn0036770 | Prestin | 1 | |  |
| male | FBgn0123851 | HE | E | FBgn0036206 | CG5964 | 1 | |  |
| male | FBgn0123864 | HE | E | FBgn0035355 | CG16985 | 1 | |  |
| male | FBgn0123872 | HE | E | FBgn0039237 | CG13640 | 1 | |  |
| male | FBgn0123882 | HE | E | FBgn0001248 | Idh | 1 | |  |
| male | FBgn0123883 | HE | E | FBgn0265266 | CG13639 | 1 | |  |
| male | FBgn0123888 | HE | E | FBgn0259224 | CG42324 | 1 | |  |
| male | FBgn0123900 | LE | E | FBgn0004875 | enc | 1 | |  |
| male | FBgn0123914 | HE | E | FBgn0011016 | SsRbeta | 1 | |  |
| male | FBgn0123928 | HE | E | FBgn0004465 | Su(P) | 1 | |  |
| male | FBgn0123933 | LE | E | FBgn0035625 | Blimp-1 | 1 | |  |
| male | FBgn0123938 | LE | E | FBgn0016756 | Usp47 | 1 | |  |
| male | FBgn0123945 | LE | E | FBgn0264002 | MsR2 | 1 | |  |
| male | FBgn0123949 | HE | E | FBgn0034472 | CG8517 | 1 | |  |
| male | FBgn0123949 | HE | E | FBgn0035334 | CG8993 | 1 | |  |
| male | FBgn0123963 | LE | E | --- | --- | 1 | |  |
| male | FBgn0123966 | LE | E | --- | --- | 1 | |  |
| male | FBgn0123983 | LE | E | FBgn0003041 | pbl | 1 | |  |
| male | FBgn0123991 | HE | E | FBgn0046258 | CG12880 | 1 | |  |
| male | FBgn0123995 | HE | E | FBgn0037061 | CG12975 | 1 | |  |
| male | FBgn0124001 | LE | E | --- | --- | 1 | |  |
| male | FBgn0124006 | LE | E | FBgn0035945 | CG5026 | 1 | |  |
| male | FBgn0124039 | LE/HE | E | --- | --- | 1 | |  |
| male | FBgn0124071 | HE | E | --- | --- | 1 | |  |
| male | FBgn0124074 | HE | E | --- | --- | 1 | |  |
| male | FBgn0124078 | HE | E | FBgn0261679 | CG42726 | 1 | |  |
| male | FBgn0124084 | HE | E | FBgn0010333 | Rac1 | 1 | |  |
| male | FBgn0124084 | HE | E | FBgn0014011 | Rac2 | 1 | |  |
| male | FBgn0124089 | LE | E | FBgn0038744 | CG4733 | 1 | |  |
| male | FBgn0124090 | HE | E | FBgn0038324 | CG5038 | 1 | |  |
| male | FBgn0124104 | HE | E | FBgn0036180 | CG6091 | 1 | |  |
| male | FBgn0124105 | LE | E | FBgn0003292 | rt | 1 | |  |
| male | FBgn0124124 | HE | E | --- | --- | 1 | |  |
| male | FBgn0124144 | LE | E | FBgn0039500 | CG5984 | 1 | |  |
| male | FBgn0124150 | LE | E | FBgn0036377 | CG10710 | 1 | |  |
| male | FBgn0124151 | HE | E | FBgn0086785 | Vps36 | 1 | |  |
| male | FBgn0124164 | LE | E | FBgn0037165 | CG11437 | 1 | |  |
| male | FBgn0124165 | HE | E | FBgn0037166 | CG11426 | 1 | |  |
| male | FBgn0124206 | HE | E | FBgn0036853 | mRpL21 | 1 | |  |
| male | FBgn0124212 | HE | E | FBgn0015283 | Rpn10 | 1 | |  |
| male | FBgn0124234 | LE | E | FBgn0037185 | CG11367 | 1 | |  |
| male | FBgn0124235 | LE | E | FBgn0037188 | CG7369 | 1 | |  |
| male | FBgn0124243 | HE | E | FBgn0053170 | CG33170 | 1 | |  |
| male | FBgn0124247 | HE | E | FBgn0037202 | Ssl1 | 1 | |  |
| male | FBgn0124270 | LE | E | FBgn0036436 | CG4914 | 1 | |  |
| male | FBgn0124278 | HE | E | FBgn0036428 | Gbs-70E | 1 | |  |
| male | FBgn0124292 | HE | E | FBgn0264561 | Glg1 | 1 | |  |
| male | FBgn0124308 | LE | E | --- | --- | 1 | |  |
| male | FBgn0124313 | HE | E | FBgn0036298 | nst | 1 | |  |
| male | FBgn0124334 | HE | E | FBgn0001224 | Hsp23 | 1 | |  |
| male | FBgn0124335 | HE | E | --- | --- | 1 | |  |
| male | FBgn0124338 | HE | E | FBgn0035169 | CG13890 | 1 | |  |
| male | FBgn0124342 | LE | E | FBgn0028999 | nerfin-1 | 1 | |  |
| male | FBgn0124354 | HE | E | FBgn0052267 | CG32267 | 1 | |  |
| male | FBgn0124360 | LE | E | FBgn0035647 | CG10486 | 1 | |  |
| male | FBgn0124363 | HE | E | FBgn0014002 | Pdi | 1 | |  |
| male | FBgn0124366 | HE | E | FBgn0019925 | Surf4 | 1 | |  |
| male | FBgn0124367 | HE | E | FBgn0260049 | flr | 1 | |  |
| male | FBgn0124368 | LE | E | FBgn0036362 | CG10725 | 1 | |  |
| male | FBgn0124383 | HE | E | FBgn0036910 | Cyp305a1 | 1 | |  |
| male | FBgn0124390 | LE | E | FBgn0022959 | yps | 1 | |  |
| male | FBgn0124400 | HE | E | --- | --- | 1 | |  |
| male | FBgn0124401 | HE | E | FBgn0036135 | mRpL2 | 1 | |  |
| male | FBgn0124403 | LE | E | FBgn0052082 | CG32082 | 1 | |  |
| male | FBgn0124404 | HE | E | --- | --- | 1 | |  |
| male | FBgn0124405 | HE | E | FBgn0001120 | gnu | 1 | |  |
| male | FBgn0124413 | HE | E | FBgn0029094 | asf1 | 1 | |  |
| male | FBgn0124418 | HE | E | FBgn0036850 | Gem2 | 1 | |  |
| male | FBgn0124419 | HE | E | FBgn0036847 | CG11577 | 1 | |  |
| male | FBgn0124425 | LE | E | FBgn0002945 | nkd | 1 | |  |
| male | FBgn0124429 | HE | E | FBgn0036836 | CG11619 | 1 | |  |
| male | FBgn0124449 | HE | E | FBgn0037017 | CG4074 | 1 | |  |
| male | FBgn0124454 | LE | E | FBgn0037012 | Rcd2 | 1 | |  |
| male | FBgn0124457 | HE | E | FBgn0011769 | Fdx1 | 1 | |  |
| male | FBgn0124460 | HE | E | FBgn0035375 | pgant6 | 1 | |  |
| male | FBgn0124479 | HE | E | FBgn0001224 | Hsp23 | 1 | |  |
| male | FBgn0124483 | LE | E | FBgn0037007 | CG5059 | 1 | |  |
| male | FBgn0124484 | HE | E | FBgn0037000 | ZnT77C | 1 | |  |
| male | FBgn0124489 | LE | E | FBgn0036992 | CG11796 | 1 | |  |
| male | FBgn0124504 | HE | E | FBgn0035644 | DNApol-epsilon58 | 1 | |  |
| male | FBgn0124549 | HE | E | FBgn0036334 | CG11267 | 1 | |  |
| male | FBgn0124567 | LE | E | FBgn0036416 | CG7924 | 1 | |  |
| male | FBgn0124590 | LE | E | FBgn0003997 | hid | 1 | |  |
| male | FBgn0124594 | HE | E | FBgn0036775 | CG5147 | 1 | |  |
| male | FBgn0124608 | HE | E | FBgn0037138 | P5CDh1 | 1 | |  |
| male | FBgn0124627 | HE | E | FBgn0038806 | CG5412 | 1 | |  |
| male | FBgn0124630 | LE | E | FBgn0036350 | CG14111 | 1 | |  |
| male | FBgn0124634 | HE | E | FBgn0028573 | prc | 1 | |  |
| male | FBgn0124636 | HE | E | FBgn0260857 | Bet1 | 1 | |  |
| male | FBgn0124640 | HE | E | FBgn0036856 | CG9666 | 1 | |  |
| male | FBgn0124641 | LE | E | FBgn0036859 | CG14085 | 1 | |  |
| male | FBgn0124648 | HE | E | FBgn0036879 | Cpr76Bb | 1 | |  |
| male | FBgn0124655 | HE | E | FBgn0036927 | CG7433 | 1 | |  |
| male | FBgn0124657 | LE | E | FBgn0023097 | bon | 1 | |  |
| male | FBgn0124663 | LE | E | FBgn0036814 | CG14073 | 1 | |  |
| male | FBgn0124674 | HE | E | FBgn0036536 | CG12713 | 1 | |  |
| male | FBgn0124695 | LE | E | --- | --- | 1 | |  |
| male | FBgn0124701 | LE | E | --- | --- | 1 | |  |
| male | FBgn0124703 | LE | E | --- | --- | 1 | |  |
| male | FBgn0124709 | HE | E | FBgn0053172 | CG33172 | 1 | |  |
| male | FBgn0124713 | LE | E | --- | --- | 1 | |  |
| male | FBgn0124740 | HE | E | FBgn0038826 | Syp | 1 | |  |
| male | FBgn0124744 | HE | E | FBgn0038832 | CG15695 | 1 | |  |
| male | FBgn0124753 | HE | E | FBgn0016650 | Lgr1 | 1 | |  |
| male | FBgn0124760 | HE | E | FBgn0064119 | CG33934 | 1 | |  |
| male | FBgn0124765 | LE | E | FBgn0038579 | CG14313 | 1 | |  |
| male | FBgn0124782 | HE | E | FBgn0038847 | CG12278 | 1 | |  |
| male | FBgn0124788 | HE | E | --- | --- | 1 | |  |
| male | FBgn0124795 | LE | E | FBgn0046225 | CG17230 | 1 | |  |
| male | FBgn0124798 | HE | E | FBgn0263316 | Mrp4 | 1 | |  |
| male | FBgn0124799 | HE | E | FBgn0263316 | Mrp4 | 1 | |  |
| male | FBgn0124813 | HE | E | FBgn0010389 | htl | 1 | |  |
| male | FBgn0124816 | LE | E | FBgn0052755 | CG32755 | 1 | |  |
| male | FBgn0124818 | HE | E | FBgn0037884 | Arfip | 1 | |  |
| male | FBgn0124819 | HE | E | FBgn0037882 | CG17187 | 1 | |  |
| male | FBgn0124825 | HE | E | FBgn0051390 | MED7 | 1 | |  |
| male | FBgn0124838 | HE | E | FBgn0039313 | CG11892 | 1 | |  |
| male | FBgn0124844 | HE | E | FBgn0051460 | CG31460 | 1 | |  |
| male | FBgn0124852 | LE | E | FBgn0039299 | CG11854 | 1 | |  |
| male | FBgn0124855 | LE | E | FBgn0038295 | Gyc88E | 1 | |  |
| male | FBgn0124879 | HE | E | FBgn0037747 | CG8481 | 1 | |  |
| male | FBgn0124901 | LE | E | FBgn0028646 | aralar1 | 1 | |  |
| male | FBgn0124905 | HE | E | FBgn0039766 | CG15536 | 1 | |  |
| male | FBgn0124915 | HE | E | FBgn0039580 | Gfat2 | 1 | |  |
| male | FBgn0124918 | HE | E | FBgn0038235 | CG8461 | 1 | |  |
| male | FBgn0124927 | HE | E | FBgn0051148 | Gba1a | 1 | |  |
| male | FBgn0124929 | HE | E | FBgn0039106 | CG10301 | 1 | |  |
| male | FBgn0124943 | LE | E | FBgn0037275 | CG14655 | 1 | |  |
| male | FBgn0124962 | LE | E | FBgn0038510 | CG14331 | 1 | |  |
| male | FBgn0124966 | LE | E | FBgn0038504 | Sur-8 | 1 | |  |
| male | FBgn0124974 | HE | E | FBgn0038966 | pinta | 1 | |  |
| male | FBgn0124975 | HE | E | FBgn0038964 | Nop56 | 1 | |  |
| male | FBgn0124999 | LE | E | --- | --- | 1 | |  |
| male | FBgn0125014 | HE | E | FBgn0032139 | CG13116 | 1 | |  |
| male | FBgn0125019 | LE | E | FBgn0032772 | CG17350 | 1 | |  |
| male | FBgn0125022 | LE | E | --- | --- | 1 | |  |
| male | FBgn0125026 | LE | E | FBgn0030376 | CG2750 | 1 | |  |
| male | FBgn0125029 | LE | E | FBgn0267001 | Ten-a | 1 | |  |
| male | FBgn0125030 | LE | E | --- | --- | 1 | |  |
| male | FBgn0125049 | LE | E | FBgn0030893 | RhoGAP16F | 1 | |  |
| male | FBgn0125055 | HE | E | FBgn0015036 | Cyp4ae1 | 1 | |  |
| male | FBgn0125057 | HE | E | FBgn0054015 | CG34015 | 1 | |  |
| male | FBgn0125058 | HE | E | FBgn0037602 | SLIRP2 | 1 | |  |
| male | FBgn0125064 | LE | E | FBgn0003366 | sev | 1 | |  |
| male | FBgn0125080 | HE | E | FBgn0037606 | CG8032 | 1 | |  |
| male | FBgn0125086 | LE | E | FBgn0261703 | gce | 1 | |  |
| male | FBgn0125088 | LE | E | FBgn0030432 | CG4404 | 1 | |  |
| male | FBgn0125120 | HE | E | FBgn0044030 | mRpS14 | 1 | |  |
| male | FBgn0125122 | HE | E | FBgn0031021 | ND-18 | 1 | |  |
| male | FBgn0125136 | LE | E | --- | --- | 1 | |  |
| male | FBgn0125138 | LE | E | --- | --- | 1 | |  |
| male | FBgn0125140 | HE | E | FBgn0024238 | Fim | 1 | |  |
| male | FBgn0125141 | HE | E | FBgn0029594 | CG14806 | 1 | |  |
| male | FBgn0125149 | LE | E | --- | --- | 1 | |  |
| male | FBgn0125162 | LE | E | --- | --- | 1 | |  |
| male | FBgn0125173 | HE | E | FBgn0029712 | CG15912 | 1 | |  |
| male | FBgn0125183 | HE | E | FBgn0265413 | CG44325 | 1 | |  |
| male | FBgn0125188 | LE | E | FBgn0083228 | Frq2 | 1 | |  |
| male | FBgn0125195 | LE | E | FBgn0030914 | CG6106 | 1 | |  |
| male | FBgn0125215 | HE | E | FBgn0261675 | Npc1b | 1 | |  |
| male | FBgn0125216 | HE | E | FBgn0040931 | CG9034 | 1 | |  |
| male | FBgn0125219 | LE | E | FBgn0037304 | CG1113 | 1 | |  |
| male | FBgn0125230 | LE | E | FBgn0250874 | ttm50 | 1 | |  |
| male | FBgn0125247 | LE | E | FBgn0031118 | RhoGAP19D | 1 | |  |
| male | FBgn0125250 | LE | E | --- | --- | 1 | |  |
| male | FBgn0125272 | HE | E | FBgn0030082 | HP1b | 1 | |  |
| male | FBgn0125278 | HE | E | FBgn0024807 | DIP1 | 1 | |  |
| male | FBgn0125279 | HE | E | FBgn0052500 | CG32500 | 1 | |  |
| male | FBgn0125279 | HE | E | FBgn0052857 | CG32857 | 1 | |  |
| male | FBgn0125279 | HE | E | FBgn0053502 | CG33502 | 1 | |  |
| male | FBgn0125299 | LE | E | --- | --- | 1 | |  |
| male | FBgn0125305 | LE | E | --- | --- | 1 | |  |
| male | FBgn0125320 | HE | E | FBgn0028969 | deltaCOP | 1 | |  |
| male | FBgn0125336 | LE | E | FBgn0027291 | l(1)G0156 | 1 | |  |
| male | FBgn0125336 | LE | E | FBgn0052026 | CG32026 | 1 | |  |
| male | FBgn0125338 | HE | E | FBgn0040890 | ksh | 1 | |  |
| male | FBgn0125348 | LE | E | --- | --- | 1 | |  |
| male | FBgn0125357 | LE | E | FBgn0037781 | Fancl | 1 | |  |
| male | FBgn0125362 | LE | E | FBgn0011837 | Tis11 | 1 | |  |
| male | FBgn0125365 | HE | E | FBgn0029969 | CG10932 | 1 | |  |
| male | FBgn0125378 | HE | E | FBgn0037777 | CG11722 | 1 | |  |
| male | FBgn0125408 | LE | E | FBgn0030870 | CG6398 | 1 | |  |
| male | FBgn0125409 | HE | E | FBgn0040285 | Scamp | 1 | |  |
| male | FBgn0125423 | HE | E | FBgn0025394 | inc | 1 | |  |
| male | FBgn0125434 | LE | E | FBgn0030386 | CG2574 | 1 | |  |
| male | FBgn0125455 | HE | E | --- | --- | 1 | |  |
| male | FBgn0125464 | LE | E | FBgn0032681 | CG10283 | 1 | |  |
| male | FBgn0125468 | LE | E | --- | --- | 1 | |  |
| male | FBgn0125489 | LE | E | --- | --- | 1 | |  |
| male | FBgn0125491 | LE | E | FBgn0039272 | CG11836 | 1 | |  |
| male | FBgn0125492 | HE | E | FBgn0039274 | CG11920 | 1 | |  |
| male | FBgn0125497 | HE | E | FBgn0011770 | Gip | 1 | |  |
| male | FBgn0125504 | HE | E | FBgn0004898 | fd96Cb | 1 | |  |
| male | FBgn0125505 | HE | E | FBgn0039266 | CG11791 | 1 | |  |
| male | FBgn0125506 | LE | E | --- | --- | 1 | |  |
| male | FBgn0125508 | HE | E | FBgn0037723 | SpdS | 1 | |  |
| male | FBgn0125513 | HE | E | FBgn0052230 | ND-MLRQ | 1 | |  |
| male | FBgn0125516 | LE | E | --- | --- | 1 | |  |
| male | FBgn0125523 | LE | E | FBgn0039290 | CG13654 | 1 | |  |
| male | FBgn0125528 | HE | E | FBgn0037835 | CG14687 | 1 | |  |
| male | FBgn0125531 | LE | E | FBgn0015589 | Apc | 1 | |  |
| male | FBgn0125533 | LE | E | FBgn0038475 | Keap1 | 1 | |  |
| male | FBgn0125537 | HE | E | FBgn0038461 | CG3678 | 1 | |  |
| male | FBgn0125537 | HE | E | FBgn0038462 | CG17556 | 1 | |  |
| male | FBgn0125547 | HE | E | FBgn0261286 | Mat89Ba | 1 | |  |
| male | FBgn0125553 | LE | E | FBgn0037443 | Dmtn | 1 | |  |
| male | FBgn0125589 | LE | E | FBgn0283499 | InR | 1 | |  |
| male | FBgn0125592 | HE | E | FBgn0262801 | twr | 1 | |  |
| male | FBgn0125598 | LE | E | FBgn0004054 | zen2 | 1 | |  |
| male | FBgn0125611 | HE | E | FBgn0028833 | Dak1 | 1 | |  |
| male | FBgn0125625 | HE | E | FBgn0037377 | CG1218 | 1 | |  |
| male | FBgn0125626 | HE | E | FBgn0038453 | CG10326 | 1 | |  |
| male | FBgn0125628 | HE | E | FBgn0038675 | CG6013 | 1 | |  |
| male | FBgn0125645 | LE | E | FBgn0039424 | ppk15 | 1 | |  |
| male | FBgn0125652 | HE | E | FBgn0250820 | meigo | 1 | |  |
| male | FBgn0125655 | LE | E | --- | --- | 1 | |  |
| male | FBgn0125658 | HE | E | FBgn0053105 | p24-2, eca | 1 | |  |
| male | FBgn0125658 | HE | E | FBgn0069242 | p24-2, eca | 1 | |  |
| male | FBgn0125663 | LE | E | FBgn0266758 | Esyt2 | 1 | |  |
| male | FBgn0125665 | LE | E | FBgn0263490 | mld | 1 | |  |
| male | FBgn0125668 | LE | E | FBgn0038348 | AOX2 | 1 | |  |
| male | FBgn0125686 | HE | E | FBgn0038359 | CG5614 | 1 | |  |
| male | FBgn0125687 | HE | E | FBgn0038360 | CG9590 | 1 | |  |
| male | FBgn0125714 | HE | E | FBgn0038426 | mRpS33 | 1 | |  |
| male | FBgn0125718 | LE | E | FBgn0038422 | CG14880 | 1 | |  |
| male | FBgn0125721 | LE | E | FBgn0037634 | hng2 | 1 | |  |
| male | FBgn0125723 | HE | E | FBgn0028468 | rtet | 1 | |  |
| male | FBgn0125733 | HE | E | FBgn0037930 | CG14715 | 1 | |  |
| male | FBgn0125744 | LE | E | FBgn0038851 | dmrt93B | 1 | |  |
| male | FBgn0125751 | HE | E | FBgn0037717 | CG8301 | 1 | |  |
| male | FBgn0125752 | HE | E | FBgn0037718 | P58IPK | 1 | |  |
| male | FBgn0125753 | HE | E | FBgn0017567 | ND-23 | 1 | |  |
| male | FBgn0125756 | LE | E | FBgn0038432 | CG14883 | 1 | |  |
| male | FBgn0125774 | HE | E | FBgn0037501 | Ir84a | 1 | |  |
| male | FBgn0125777 | HE | E | --- | --- | 1 | |  |
| male | FBgn0125799 | LE | E | FBgn0053110 | CG33110 | 1 | |  |
| male | FBgn0125807 | HE | E | FBgn0024432 | Dlc90F | 1 | |  |
| male | FBgn0125825 | LE | E | FBgn0038203 | Or88a | 1 | |  |
| male | FBgn0125830 | LE | E | FBgn0038099 | CG7091 | 1 | |  |
| male | FBgn0125837 | HE | E | FBgn0002354 | l(3)87Df | 1 | |  |
| male | FBgn0125842 | HE | E | FBgn0037356 | CG12170 | 1 | |  |
| male | FBgn0125845 | LE | E | FBgn0038840 | CG5621 | 1 | |  |
| male | FBgn0125850 | LE | E | FBgn0038125 | CG8141 | 1 | |  |
| male | FBgn0125852 | HE | E | FBgn0039751 | CG1983 | 1 | |  |
| male | FBgn0125869 | HE | E | FBgn0001280 | janA | 1 | |  |
| male | FBgn0125880 | HE | E | FBgn0039562 | Gp93 | 1 | |  |
| male | FBgn0125882 | HE | E | FBgn0039764 | CG15535 | 1 | |  |
| male | FBgn0125888 | LE | E | FBgn0039757 | RpS7 | 1 | |  |
| male | FBgn0125897 | HE | E | FBgn0038136 | CG8774 | 1 | |  |
| male | FBgn0125912 | HE | E | FBgn0039218 | Rpb10 | 1 | |  |
| male | FBgn0125918 | LE | E | FBgn0259233 | CG42331 | 1 | |  |
| male | FBgn0125922 | LE | E | FBgn0038977 | CG5376 | 1 | |  |
| male | FBgn0125926 | LE | E | FBgn0037796 | CG12814 | 1 | |  |
| male | FBgn0125928 | LE | E | FBgn0027539 | lili | 1 | |  |
| male | FBgn0125932 | HE | E | FBgn0038974 | CG5377 | 1 | |  |
| male | FBgn0125943 | HE | E | FBgn0259704 | Nsun5 | 1 | |  |
| male | FBgn0125955 | HE | E | FBgn0039145 | CG6000 | 1 | |  |
| male | FBgn0125969 | LE | E | FBgn0026598 | Apc2 | 1 | |  |
| male | FBgn0125981 | HE | E | FBgn0038337 | CG6125 | 1 | |  |
| male | FBgn0125986 | LE | E | FBgn0039728 | CG7896 | 1 | |  |
| male | FBgn0126010 | LE | E | FBgn0038150 | yellow-e3 | 1 | |  |
| male | FBgn0126020 | LE/HE | E | FBgn0083953 | CG34117 | 1 | |  |
| male | FBgn0126023 | HE | E | FBgn0038585 | Non3 | 1 | |  |
| male | FBgn0126027 | HE | E | FBgn0020238 | 14-3-3epsilon | 1 | |  |
| male | FBgn0126035 | HE | E | FBgn0260005 | wtrw | 1 | |  |
| male | FBgn0126037 | HE | E | FBgn0037608 | mRpL19 | 1 | |  |
| male | FBgn0126038 | HE | E | FBgn0037612 | CG8112 | 1 | |  |
| male | FBgn0126041 | HE | E | FBgn0016685 | Nlp | 1 | |  |
| male | FBgn0126062 | HE | E | FBgn0037329 | POLDIP2 | 1 | |  |
| male | FBgn0126070 | HE | E | FBgn0039357 | CG4743 | 1 | |  |
| male | FBgn0126077 | HE | E | FBgn0039348 | Npl4 | 1 | |  |
| male | FBgn0126088 | HE | E | FBgn0051198 | CG31198 | 1 | |  |
| male | FBgn0126117 | HE | E | FBgn0039868 | CG11563 | 1 | |  |
| male | FBgn0126118 | LE | E | FBgn0000557 | Ef1alpha100E | 1 | |  |
| male | FBgn0126128 | LE | E | FBgn0039020 | CG17141 | 1 | |  |
| male | FBgn0126143 | HE | E | FBgn0039627 | CG11837 | 1 | |  |
| male | FBgn0126165 | HE | E | FBgn0040623 | Spase12 | 1 | |  |
| male | FBgn0126178 | LE | E | FBgn0045469 | Gr93c | 1 | |  |
| male | FBgn0126179 | HE | E | FBgn0045470 | Gr93b | 1 | |  |
| male | FBgn0126180 | LE | E | --- | --- | 1 | |  |
| male | FBgn0126202 | LE | E | FBgn0037236 | Skp2 | 1 | |  |
| male | FBgn0126209 | HE | E | FBgn0037845 | CG14694 | 1 | |  |
| male | FBgn0126210 | HE | E | FBgn0038318 | CG6236 | 1 | |  |
| male | FBgn0126212 | HE | E | FBgn0011476 | l(3)neo43 | 1 | |  |
| male | FBgn0126220 | HE | E | FBgn0038306 | Art3 | 1 | |  |
| male | FBgn0126221 | HE | E | FBgn0051344 | CG31344 | 1 | |  |
| male | FBgn0126222 | HE | E | FBgn0051155 | Rpb7 | 1 | |  |
| male | FBgn0126232 | LE | E | FBgn0010379 | Akt1 | 1 | |  |
| male | FBgn0126235 | LE | E | FBgn0063649 | CG6006 | 1 | |  |
| male | FBgn0126240 | LE | E | --- | --- | 1 | |  |
| male | FBgn0126247 | HE | E | --- | --- | 1 | |  |
| male | FBgn0126249 | HE | E | FBgn0039205 | CG13623 | 1 | |  |
| male | FBgn0126253 | LE | E | --- | --- | 1 | |  |
| male | FBgn0126273 | LE | E | --- | --- | 1 | |  |
| male | FBgn0126279 | HE | E | FBgn0039184 | CG6432 | 1 | |  |
| male | FBgn0126290 | LE | E | FBgn0039199 | CG13615 | 1 | |  |
| male | FBgn0126297 | LE | E | FBgn0262167 | ana1 | 1 | |  |
| male | FBgn0126300 | HE | E | FBgn0010438 | mtSSB | 1 | |  |
| male | FBgn0126304 | HE | E | FBgn0038400 | CG5903 | 1 | |  |
| male | FBgn0126305 | LE | E | FBgn0026059 | Mhcl | 1 | |  |
| male | FBgn0126306 | LE | E | --- | --- | 1 | |  |
| male | FBgn0126310 | LE | E | FBgn0038302 | CG4210 | 1 | |  |
| male | FBgn0126317 | LE | E | --- | --- | 1 | |  |
| male | FBgn0126322 | HE | E | FBgn0038313 | CG4338 | 1 | |  |
| male | FBgn0126348 | HE | E | FBgn0038194 | Cyp6d5 | 1 | |  |
| male | FBgn0126350 | LE | E | FBgn0038880 | SIFaR | 1 | |  |
| male | FBgn0126364 | LE | E | FBgn0250910 | Octbeta3R | 1 | |  |
| male | FBgn0126370 | HE | E | FBgn0039674 | CG1907 | 1 | |  |
| male | FBgn0126384 | HE | E | FBgn0039175 | beta-PheRS | 1 | |  |
| male | FBgn0126385 | HE | E | FBgn0039172 | Spase22-23 | 1 | |  |
| male | FBgn0126410 | LE | E | FBgn0039857 | RpL6 | 1 | |  |
| male | FBgn0126425 | LE/HE | E | FBgn0039474 | CG6283 | 1 | |  |
| male | FBgn0126427 | LE | E | FBgn0039474 | CG6283 | 1 | |  |
| male | FBgn0126437 | HE | E | --- | --- | 1 | |  |
| male | FBgn0126438 | HE | E | FBgn0051472 | sgll | 1 | |  |
| male | FBgn0126463 | HE | E | FBgn0025825 | HDAC3 | 1 | |  |
| male | FBgn0126466 | HE | E | FBgn0037468 | CG1943 | 1 | |  |
| male | FBgn0126472 | HE | E | FBgn0051248 | CG31248 | 1 | |  |
| male | FBgn0126475 | LE | E | FBgn0011020 | Sas-4 | 1 | |  |
| male | FBgn0126489 | HE | E | FBgn0014930 | CG2846 | 1 | |  |
| male | FBgn0126501 | HE | E | FBgn0038584 | mTerf5 | 1 | |  |
| male | FBgn0126510 | HE | E | FBgn0038145 | Droj2 | 1 | |  |
| male | FBgn0126535 | LE | E | FBgn0039094 | CG10184 | 1 | |  |
| male | FBgn0126546 | LE | E | FBgn0038342 | B9d1 | 1 | |  |
| male | FBgn0126572 | HE | E | FBgn0037819 | CG14688 | 1 | |  |
| male | FBgn0126573 | LE | E | FBgn0001235 | hth | 1 | |  |
| male | FBgn0126589 | HE | E | FBgn0038319 | mRpL9 | 1 | |  |
| male | FBgn0126602 | LE | E | --- | --- | 1 | |  |
| male | FBgn0126606 | HE | E | --- | --- | 1 | |  |
| male | FBgn0126607 | LE | E | FBgn0037696 | GstZ1 | 1 | |  |
| male | FBgn0126611 | HE | E | FBgn0021750 | SerRS-m | 1 | |  |
| male | FBgn0126614 | LE | E | FBgn0038132 | CG15887 | 1 | |  |
| male | FBgn0126625 | LE | E | FBgn0010113 | hdc | 1 | |  |
| male | FBgn0126626 | LE | E | --- | --- | 1 | |  |
| male | FBgn0126644 | HE | E | FBgn0260468 | CG7950 | 1 | |  |
| male | FBgn0126652 | LE | E | FBgn0038127 | CG8476 | 1 | |  |
| male | FBgn0126654 | LE | E | FBgn0038126 | CG8483 | 1 | |  |
| male | FBgn0126665 | HE | E | FBgn0038110 | CG8031 | 1 | |  |
| male | FBgn0126668 | HE | E | FBgn0038098 | CG7381 | 1 | |  |
| male | FBgn0126697 | HE | E | FBgn0038608 | WRNexo | 1 | |  |
| male | FBgn0126700 | LE | E | FBgn0038603 | PKD | 1 | |  |
| male | FBgn0126701 | LE | E | --- | --- | 1 | |  |
| male | FBgn0126709 | HE | E | FBgn0265274 | Inx3 | 1 | |  |
| male | FBgn0126716 | LE | E | FBgn0038897 | CG5849 | 1 | |  |
| male | FBgn0126719 | HE | E | FBgn0051233 | CG31233 | 1 | |  |
| male | FBgn0126720 | HE | E | FBgn0051198 | CG31198 | 1 | |  |
| male | FBgn0126724 | LE | E | FBgn0042104 | CG18747 | 1 | |  |
| male | FBgn0126732 | HE | E | FBgn0015572 | alpha-Est4 | 1 | |  |
| male | FBgn0126741 | HE | E | FBgn0015577 | alpha-Est9 | 1 | |  |
| male | FBgn0126747 | LE | E | FBgn0001112 | Gld | 1 | |  |
| male | FBgn0126757 | LE | E | FBgn0004087 | Dhfr | 1 | |  |
| male | FBgn0126785 | HE | E | FBgn0037935 | CG6834 | 1 | |  |
| male | FBgn0126796 | LE | E | FBgn0037636 | CG9821 | 1 | |  |
| male | FBgn0126797 | HE | E | FBgn0037635 | CG9837 | 1 | |  |
| male | FBgn0126805 | LE | E | FBgn0039851 | mey | 1 | |  |
| male | FBgn0126812 | HE | E | FBgn0037661 | Ada | 1 | |  |
| male | FBgn0126881 | LE | E | FBgn0263118 | tx | 1 | |  |
| male | FBgn0126886 | LE | E | FBgn0040250 | Ugt86Dj | 1 | |  |
| male | FBgn0126893 | HE | E | FBgn0039252 | CG11771 | 1 | |  |
| male | FBgn0126894 | HE | E | FBgn0042094 | Adk3 | 1 | |  |
| male | FBgn0126900 | HE | E | FBgn0037378 | CG2046 | 1 | |  |
| male | FBgn0126910 | HE | E | ------ | ------ | 1 | |  |
| male | FBgn0126933 | HE | E | FBgn0051086 | CG31086 | 1 | |  |
| male | FBgn0126933 | HE | E | FBgn0052633 | CG32633 | 1 | |  |
| male | FBgn0126942 | HE | E | FBgn0003339 | Scr | 1 | |  |
| male | FBgn0126948 | HE | E | FBgn0026737 | CG6171 | 1 | |  |
| male | FBgn0126954 | HE | E | FBgn0004778 | Ccp84Af | 1 | |  |
| male | FBgn0126958 | HE | E | FBgn0038893 | Archease | 1 | |  |
| male | FBgn0126962 | HE | E | FBgn0037263 | slx1 | 1 | |  |
| male | FBgn0126969 | HE | E | FBgn0037363 | Atg17 | 1 | |  |
| male | FBgn0126974 | LE | E | FBgn0037921 | CG6808 | 1 | |  |
| male | FBgn0126980 | HE | E | --- | --- | 1 | |  |
| male | FBgn0127002 | LE | E | FBgn0037446 | Zif | 1 | |  |
| male | FBgn0127003 | HE | E | --- | --- | 1 | |  |
| male | FBgn0127019 | LE | E | FBgn0004577 | Pxd | 1 | |  |
| male | FBgn0127043 | HE | E | FBgn0005585 | Calr | 1 | |  |
| male | FBgn0127048 | HE | E | FBgn0039271 | CG11839 | 1 | |  |
| male | FBgn0127049 | HE | E | FBgn0011336 | OstStt3 | 1 | |  |
| male | FBgn0127050 | LE | E | --- | --- | 1 | |  |
| male | FBgn0127054 | LE | E | FBgn0263977 | Tim17b | 1 | |  |
| male | FBgn0127056 | LE | E | FBgn0023527 | CG3071 | 1 | |  |
| male | FBgn0127058 | HE | E | --- | --- | 1 | |  |
| male | FBgn0127086 | LE | E | FBgn0031926 | CG6739 | 1 | |  |
| male | FBgn0127106 | LE | E | FBgn0031645 | CG3036 | 1 | |  |
| male | FBgn0127115 | HE | E | FBgn0039636 | Atg14 | 1 | |  |
| male | FBgn0127130 | HE | E | FBgn0040001 | FASN3 | 1 | |  |
| male | FBgn0127144 | HE | E | FBgn0001098 | Gdh | 1 | |  |
| male | FBgn0127190 | HE | E | FBgn0014269 | prod | 1 | |  |
| male | FBgn0127201 | LE | E | --- | --- | 1 | |  |
| male | FBgn0127202 | LE | E | --- | --- | 1 | |  |
| male | FBgn0127210 | LE | E | FBgn0033932 | Dh44-R1 | 1 | |  |
| male | FBgn0127219 | HE | E | FBgn0001308 | Khc | 1 | |  |
| male | FBgn0127227 | LE | E | FBgn0260768 | CG42566 | 1 | |  |
| male | FBgn0127229 | HE | E | --- | --- | 1 | |  |
| male | FBgn0127230 | HE | E | FBgn0034761 | CG4250 | 1 | |  |
| male | FBgn0127231 | HE | E | FBgn0034761 | CG4250 | 1 | |  |
| male | FBgn0127236 | HE | E | FBgn0034763 | RYBP | 1 | |  |
| male | FBgn0127256 | HE | E | FBgn0034782 | CG12490 | 1 | |  |
| male | FBgn0127256 | HE | E | FBgn0034783 | CG9825 | 1 | |  |
| male | FBgn0127256 | HE | E | FBgn0050265 | CG30265 | 1 | |  |
| male | FBgn0127260 | HE | E | --- | --- | 1 | |  |
| male | FBgn0127262 | HE | E | FBgn0010651 | MFS14 | 1 | |  |
| male | FBgn0127268 | HE | E | FBgn0034382 | CG18609 | 1 | |  |
| male | FBgn0127280 | HE | E | FBgn0033463 | CG1513 | 1 | |  |
| male | FBgn0127289 | HE | E | FBgn0010590 | Prosbeta1 | 1 | |  |
| male | FBgn0127292 | HE | E | FBgn0034061 | CG8386 | 1 | |  |
| male | FBgn0127293 | HE | E | FBgn0020236 | ATPCL | 1 | |  |
| male | FBgn0127299 | HE | E | FBgn0022343 | CG3760 | 1 | |  |
| male | FBgn0127304 | LE | E | FBgn0035091 | CG3829 | 1 | |  |
| male | FBgn0127308 | LE | E | FBgn0003701 | thr | 1 | |  |
| male | FBgn0127309 | HE | E | FBgn0026316 | Ubc10 | 1 | |  |
| male | FBgn0127311 | HE | E | FBgn0038924 | CG6028 | 1 | |  |
| male | FBgn0127313 | LE | E | FBgn0028740 | CG6362 | 1 | |  |
| male | FBgn0127345 | LE | E | FBgn0034552 | CG17999 | 1 | |  |
| male | FBgn0127359 | HE | E | FBgn0061361 | CG33786 | 1 | |  |
| male | FBgn0127361 | LE | E | FBgn0034530 | Rcd6 | 1 | |  |
| male | FBgn0127365 | LE | E | FBgn0034110 | Atg9 | 1 | |  |
| male | FBgn0127366 | HE | E | FBgn0038916 | dnd | 1 | |  |
| male | FBgn0127368 | LE | E | FBgn0085428 | Nox | 1 | |  |
| male | FBgn0127383 | LE | E | FBgn0017558 | Pdk | 1 | |  |
| male | FBgn0127394 | HE | E | FBgn0033422 | Or45b | 1 | |  |
| male | FBgn0127399 | HE | E | FBgn0033584 | CG7737 | 1 | |  |
| male | FBgn0127400 | LE | E | --- | --- | 1 | |  |
| male | FBgn0127401 | HE | E | --- | --- | 1 | |  |
| male | FBgn0127409 | HE | E | --- | --- | 1 | |  |
| male | FBgn0127416 | HE | E | FBgn0011227 | ox | 1 | |  |
| male | FBgn0127419 | HE | E | FBgn0033769 | CG8768 | 1 | |  |
| male | FBgn0127420 | HE | E | FBgn0019972 | Drice | 1 | |  |
| male | FBgn0127439 | HE | E | FBgn0019643 | Dat | 1 | |  |
| male | FBgn0127445 | LE | E | FBgn0263593 | Lpin | 1 | |  |
| male | FBgn0127479 | HE | E | FBgn0033208 | mRpL52 | 1 | |  |
| male | FBgn0127480 | LE | E | FBgn0033207 | CG12826 | 1 | |  |
| male | FBgn0127487 | HE | E | FBgn0004132 | boca | 1 | |  |
| male | FBgn0127506 | HE | E | FBgn0261276 | Opa1 | 1 | |  |
| male | FBgn0127523 | HE | E | FBgn0034503 | MED8 | 1 | |  |
| male | FBgn0127539 | HE | E | FBgn0029006 | Smurf | 1 | |  |
| male | FBgn0127548 | HE | E | FBgn0022027 | Vps25 | 1 | |  |
| male | FBgn0127551 | LE | E | FBgn0003892 | ptc | 1 | |  |
| male | FBgn0127554 | LE | E | FBgn0034720 | Liprin-gamma | 1 | |  |
| male | FBgn0127555 | LE | E | FBgn0017482 | T3dh | 1 | |  |
| male | FBgn0127578 | HE | E | FBgn0003660 | Syb | 1 | |  |
| male | FBgn0127586 | LE | E | FBgn0033494 | KCNQ | 1 | |  |
| male | FBgn0127604 | HE | E | FBgn0033100 | CG3420 | 1 | |  |
| male | FBgn0127607 | HE | E | FBgn0033097 | Zip42C.2 | 1 | |  |
| male | FBgn0127650 | HE | E | FBgn0001149 | GstD1 | 1 | |  |
| male | FBgn0127676 | HE | E | FBgn0011241 | cbx | 1 | |  |
| male | FBgn0127681 | HE | E | FBgn0013983 | imd | 1 | |  |
| male | FBgn0127698 | LE | E | --- | --- | 1 | |  |
| male | FBgn0127706 | HE | E | FBgn0038447 | CG14892 | 1 | |  |
| male | FBgn0127710 | HE | E | --- | --- | 1 | |  |
| male | FBgn0127711 | HE | E | FBgn0263260 | sel | 1 | |  |
| male | FBgn0127718 | LE | E | FBgn0028494 | CG6424 | 1 | |  |
| male | FBgn0127726 | LE | E | FBgn0033362 | CG8172 | 1 | |  |
| male | FBgn0127735 | HE | E | --- | --- | 1 | |  |
| male | FBgn0127741 | LE | E | FBgn0052843 | Dh31-R | 1 | |  |
| male | FBgn0127752 | HE | E | FBgn0261014 | TER94 | 1 | |  |
| male | FBgn0127774 | LE | E | FBgn0265623 | Su(z)2 | 1 | |  |
| male | FBgn0127792 | HE | E | FBgn0035046 | ND-19 | 1 | |  |
| male | FBgn0127810 | HE | E | FBgn0034893 | mRpL43 | 1 | |  |
| male | FBgn0127813 | HE | E | FBgn0027619 | Adam | 1 | |  |
| male | FBgn0127820 | HE | E | FBgn0035063 | CG3594 | 1 | |  |
| male | FBgn0127834 | LE | E | FBgn0026389 | Or43a | 1 | |  |
| male | FBgn0127837 | LE | E | FBgn0035011 | CG13589 | 1 | |  |
| male | FBgn0127837 | LE | E | FBgn0035012 | CG13590 | 1 | |  |
| male | FBgn0127859 | HE | E | FBgn0033381 | GstE13 | 1 | |  |
| male | FBgn0127863 | HE | E | FBgn0011704 | RnrS | 1 | |  |
| male | FBgn0127864 | HE | E | FBgn0053145 | GalT1 | 1 | |  |
| male | FBgn0127868 | HE | E | FBgn0033663 | ERp60 | 1 | |  |
| male | FBgn0127874 | HE | E | FBgn0033048 | CG7881 | 1 | |  |
| male | FBgn0127895 | HE | E | FBgn0034718 | wdp | 1 | |  |
| male | FBgn0127896 | HE | E | FBgn0034716 | Oatp58Dc | 1 | |  |
| male | FBgn0127898 | HE | E | FBgn0050277 | Oatp58Da | 1 | |  |
| male | FBgn0127899 | HE | E | FBgn0050277 | Oatp58Da | 1 | |  |
| male | FBgn0127913 | LE | E | FBgn0016754 | sba | 1 | |  |
| male | FBgn0127916 | HE | E | FBgn0034293 | CG14495 | 1 | |  |
| male | FBgn0127923 | HE | E | FBgn0029134 | Prosbeta5 | 1 | |  |
| male | FBgn0127931 | HE | E | FBgn0034733 | CG4752 | 1 | |  |
| male | FBgn0127934 | HE | E | FBgn0034736 | CG6018 | 1 | |  |
| male | FBgn0127935 | LE/HE | E | --- | --- | 1 | |  |
| male | FBgn0127948 | HE | E | FBgn0034223 | Tes | 1 | |  |
| male | FBgn0127950 | LE | E | FBgn0027506 | EDTP | 1 | |  |
| male | FBgn0127956 | LE | E | FBgn0033639 | CG9003 | 1 | |  |
| male | FBgn0127959 | LE | E | FBgn0086712 | Egm | 1 | |  |
| male | FBgn0127967 | LE | E | --- | --- | 1 | |  |
| male | FBgn0127968 | HE | E | FBgn0020907 | Scp2 | 1 | |  |
| male | FBgn0127974 | LE | E | --- | --- | 1 | |  |
| male | FBgn0127977 | LE | E | FBgn0050007 | CG30007 | 1 | |  |
| male | FBgn0127982 | LE | E | FBgn0028955 | CG8788 | 1 | |  |
| male | FBgn0127987 | HE | E | FBgn0050296 | RIC-3 | 1 | |  |
| male | FBgn0127994 | HE | E | FBgn0034729 | CG10344 | 1 | |  |
| male | FBgn0128003 | LE/HE | E | FBgn0043575 | PGRP-SC2 | 1 | |  |
| male | FBgn0128005 | HE | E | FBgn0034262 | swi2 | 1 | |  |
| male | FBgn0128008 | LE | E | FBgn0265184 | CG44249 | 1 | |  |
| male | FBgn0128015 | HE | E | --- | --- | 1 | |  |
| male | FBgn0128022 | HE | E | FBgn0010038 | GstD2 | 1 | |  |
| male | FBgn0128022 | HE | E | FBgn0010040 | GstD4 | 1 | |  |
| male | FBgn0128022 | HE | E | FBgn0010041 | GstD5 | 1 | |  |
| male | FBgn0128040 | LE | E | --- | --- | 1 | |  |
| male | FBgn0128043 | LE | E | FBgn0050380 | CG30380 | 1 | |  |
| male | FBgn0128076 | HE | E | FBgn0029155 | Men-b | 1 | |  |
| male | FBgn0128094 | LE | E | FBgn0261862 | whd | 1 | |  |
| male | FBgn0128096 | LE | E | FBgn0033524 | Cyp49a1 | 1 | |  |
| male | FBgn0128098 | LE | E | FBgn0058198 | CG40198 | 1 | |  |
| male | FBgn0128099 | HE | E | FBgn0033485 | RpLP0-like | 1 | |  |
| male | FBgn0128107 | LE | E | FBgn0034131 | CG15712 | 1 | |  |
| male | FBgn0128111 | HE | E | FBgn0261266 | zuc | 1 | |  |
| male | FBgn0128113 | HE | E | FBgn0000241 | bw | 1 | |  |
| male | FBgn0128132 | HE | E | --- | --- | 1 | |  |
| male | FBgn0128133 | LE | E | FBgn0085453 | CG34424 | 1 | |  |
| male | FBgn0128136 | HE | E | FBgn0034879 | Rrp4 | 1 | |  |
| male | FBgn0128139 | HE | E | FBgn0034709 | Swim | 1 | |  |
| male | FBgn0128144 | HE | E | FBgn0034437 | CG10051 | 1 | |  |
| male | FBgn0128146 | HE | E | --- | --- | 1 | |  |
| male | FBgn0128147 | LE | E | --- | --- | 1 | |  |
| male | FBgn0128148 | LE | E | FBgn0034440 | CG10073 | 1 | |  |
| male | FBgn0128148 | LE | E | FBgn0034441 | CG10081 | 1 | |  |
| male | FBgn0128152 | HE | E | FBgn0033720 | CG13160 | 1 | |  |
| male | FBgn0128152 | HE | E | FBgn0053012 | CG33012 | 1 | |  |
| male | FBgn0128154 | HE | E | --- | --- | 1 | |  |
| male | FBgn0128163 | HE | E | FBgn0033730 | Cpr49Ag | 1 | |  |
| male | FBgn0128164 | LE | E | FBgn0033731 | Cpr49Ah | 1 | |  |
| male | FBgn0128165 | HE | E | --- | --- | 1 | |  |
| male | FBgn0128178 | HE | E | --- | --- | 1 | |  |
| male | FBgn0128181 | HE | E | FBgn0034588 | CG9394 | 1 | |  |
| male | FBgn0128191 | HE | E | --- | --- | 1 | |  |
| male | FBgn0128208 | HE | E | FBgn0034160 | CG5550 | 1 | |  |
| male | FBgn0128210 | HE | E | FBgn0025830 | IntS8 | 1 | |  |
| male | FBgn0128217 | LE/HE | E | FBgn0033961 | ND-B15 | 1 | |  |
| male | FBgn0128222 | HE | E | FBgn0085223 | CG34194 | 1 | |  |
| male | FBgn0128225 | HE | E | FBgn0260763 | CG42561 | 1 | |  |
| male | FBgn0128231 | HE | E | FBgn0039081 | Irk2 | 1 | |  |
| male | FBgn0128232 | HE | E | --- | --- | 1 | |  |
| male | FBgn0128241 | HE | E | FBgn0034660 | lox2 | 1 | |  |
| male | FBgn0128245 | HE | E | FBgn0050404 | Tango11 | 1 | |  |
| male | FBgn0128247 | LE | E | FBgn0010228 | HmgZ | 1 | |  |
| male | FBgn0128251 | LE | E | FBgn0033943 | CG12869 | 1 | |  |
| male | FBgn0128255 | HE | E | FBgn0050077 | Blos1 | 1 | |  |
| male | FBgn0128309 | HE | E | FBgn0022238 | lolal | 1 | |  |
| male | FBgn0128315 | LE | E | FBgn0033677 | CG8321 | 1 | |  |
| male | FBgn0128343 | LE | E | FBgn0010226 | GstS1 | 1 | |  |
| male | FBgn0128351 | HE | E | FBgn0019886 | Letm1 | 1 | |  |
| male | FBgn0128359 | HE | E | --- | --- | 1 | |  |
| male | FBgn0128368 | HE | E | FBgn0005638 | slbo | 1 | |  |
| male | FBgn0128372 | HE | E | FBgn0038917 | CG6678 | 1 | |  |
| male | FBgn0128374 | LE | E | FBgn0034990 | CG11406 | 1 | |  |
| male | FBgn0128375 | HE | E | FBgn0034814 | CG9890 | 1 | |  |
| male | FBgn0128376 | HE | E | FBgn0010622 | DCTN3-p24 | 1 | |  |
| male | FBgn0128394 | HE | E | FBgn0033060 | CG7849 | 1 | |  |
| male | FBgn0128401 | LE | E | FBgn0033075 | Pld | 1 | |  |
| male | FBgn0128406 | LE | E | FBgn0262867 | Ptr | 1 | |  |
| male | FBgn0128411 | HE | E | FBgn0033062 | Ars2 | 1 | |  |
| male | FBgn0128427 | LE | E | FBgn0264089 | sli | 1 | |  |
| male | FBgn0128430 | LE | E | FBgn0261564 | Reep1 | 1 | |  |
| male | FBgn0128435 | HE | E | FBgn0265187 | CG44252 | 1 | |  |
| male | FBgn0128436 | HE | E | FBgn0259937 | Nop60B | 1 | |  |
| male | FBgn0128441 | LE | E | FBgn0050418 | nord | 1 | |  |
| male | FBgn0128443 | LE | E | FBgn0034997 | CG3376 | 1 | |  |
| male | FBgn0128444 | HE | E | --- | --- | 1 | |  |
| male | FBgn0128451 | LE | E | FBgn0004101 | bs | 1 | |  |
| male | FBgn0128453 | HE | E | FBgn0002787 | Rpn8 | 1 | |  |
| male | FBgn0128470 | LE/HE | E | FBgn0034191 | CG6984 | 1 | |  |
| male | FBgn0128485 | LE | E | FBgn0010620 | CG10939 | 1 | |  |
| male | FBgn0128498 | HE | E | FBgn0033871 | CG13339 | 1 | |  |
| male | FBgn0128502 | LE | E | FBgn0033685 | OSCP1 | 1 | |  |
| male | FBgn0128506 | LE | E | FBgn0262820 | CG43191 | 1 | |  |
| male | FBgn0128532 | HE | E | FBgn0050154 | CG30154 | 1 | |  |
| male | FBgn0128554 | HE | E | FBgn0033294 | Mal-A4 | 1 | |  |
| male | FBgn0128560 | LE | E | FBgn0033302 | Cyp6a14 | 1 | |  |
| male | FBgn0128566 | LE | E | FBgn0033984 | Lap1 | 1 | |  |
| male | FBgn0128569 | LE | E | FBgn0033988 | pcs | 1 | |  |
| male | FBgn0128582 | LE | E | FBgn0010397 | LamC | 1 | |  |
| male | FBgn0128617 | LE | E | FBgn0034903 | CG9850 | 1 | |  |
| male | FBgn0128627 | HE | E | FBgn0033169 | CG11123 | 1 | |  |
| male | FBgn0128633 | HE | E | FBgn0261067 | LSm1 | 1 | |  |
| male | FBgn0128640 | HE | E | FBgn0050016 | CG30016 | 1 | |  |
| male | FBgn0128650 | HE | E | FBgn0016131 | Cdk4 | 1 | |  |
| male | FBgn0128654 | HE | E | FBgn0028683 | spt4 | 1 | |  |
| male | FBgn0128665 | HE | E | FBgn0033734 | CG8520 | 1 | |  |
| male | FBgn0128668 | HE | E | FBgn0034877 | levy | 1 | |  |
| male | FBgn0128669 | HE | E | FBgn0038049 | CG5844 | 1 | |  |
| male | FBgn0128682 | HE | E | FBgn0034884 | CG17662 | 1 | |  |
| male | FBgn0128732 | HE | E | FBgn0033547 | CG12935 | 1 | |  |
| male | FBgn0128754 | LE | E | FBgn0003082 | phr | 1 | |  |
| male | FBgn0128761 | HE | E | FBgn0033234 | MFS12 | 1 | |  |
| male | FBgn0128770 | LE | E | FBgn0034897 | Sesn | 1 | |  |
| male | FBgn0128771 | LE | E | FBgn0016078 | wun | 1 | |  |
| male | FBgn0128776 | HE | E | FBgn0033741 | CG8545 | 1 | |  |
| male | FBgn0128788 | HE | E | FBgn0023180 | Orc6 | 1 | |  |
| male | FBgn0128822 | LE | E | FBgn0034722 | Rtf1 | 1 | |  |
| male | FBgn0128823 | HE | E | FBgn0038074 | Gnmt | 1 | |  |
| male | FBgn0128838 | HE | E | FBgn0050291 | CG30291 | 1 | |  |
| male | FBgn0128845 | HE | E | FBgn0038079 | NijC | 1 | |  |
| male | FBgn0128846 | LE | E | FBgn0033627 | CG13204 | 1 | |  |
| male | FBgn0128849 | HE | E | FBgn0033635 | CG7777 | 1 | |  |
| male | FBgn0128854 | HE | E | FBgn0050035 | Tret1-1 | 1 | |  |
| male | FBgn0128866 | HE | E | FBgn0034225 | veil | 1 | |  |
| male | FBgn0128870 | LE | E | --- | --- | 1 | |  |
| male | FBgn0128875 | LE | E | FBgn0034232 | CG4866 | 1 | |  |
| male | FBgn0128885 | LE | E | FBgn0260866 | dnr1 | 1 | |  |
| male | FBgn0128891 | LE | E | FBgn0027525 | LTV1 | 1 | |  |
| male | FBgn0128895 | LE | E | FBgn0086898 | dgo | 1 | |  |
| male | FBgn0128916 | LE | E | FBgn0034717 | CG5819 | 1 | |  |
| male | FBgn0128948 | LE | E | FBgn0034267 | CG4984 | 1 | |  |
| male | FBgn0128968 | HE | E | FBgn0033133 | Tsp42Ek | 1 | |  |
| male | FBgn0128981 | HE | E | FBgn0024294 | Spn43Aa | 1 | |  |
| male | FBgn0128990 | LE | E | FBgn0003174 | pwn | 1 | |  |
| male | FBgn0128997 | LE | E | FBgn0015801 | Reg-5 | 1 | |  |
| male | FBgn0129006 | LE | E | FBgn0028473 | Non1 | 1 | |  |
| male | FBgn0129016 | LE | E | FBgn0050183 | CG30183 | 1 | |  |
| male | FBgn0129025 | HE | E | FBgn0026261 | bonsai | 1 | |  |
| male | FBgn0129026 | LE | E | FBgn0053988 | Mid1 | 1 | |  |
| male | FBgn0129027 | HE | E | FBgn0035049 | Mmp1 | 1 | |  |
| male | FBgn0129029 | HE | E | FBgn0260456 | CG4806 | 1 | |  |
| male | FBgn0129033 | HE | E | --- | --- | 1 | |  |
| male | FBgn0129045 | LE | E | --- | --- | 1 | |  |
| male | FBgn0129054 | HE | E | FBgn0039849 | CG11334 | 1 | |  |
| male | FBgn0129082 | HE | E | FBgn0002789 | Mp20 | 1 | |  |
| male | FBgn0129083 | HE | E | --- | --- | 1 | |  |
| male | FBgn0129085 | HE | E | FBgn0259219 | CG42319 | 1 | |  |
| male | FBgn0129090 | LE | E | FBgn0033355 | CG13748 | 1 | |  |
| male | FBgn0129092 | HE | E | FBgn0033357 | Tom7 | 1 | |  |
| male | FBgn0129093 | LE | E | FBgn0011300 | babo | 1 | |  |
| male | FBgn0129102 | LE | E | FBgn0011656 | Mef2 | 1 | |  |
| male | FBgn0129106 | LE | E | FBgn0262169 | magu | 1 | |  |
| male | FBgn0129129 | HE | E | FBgn0063496 | GstE4 | 1 | |  |
| male | FBgn0129130 | LE | E | FBgn0063491-94 | GstE5 | 1 | |  |
| male | FBgn0129130 | LE | E | FBgn0063492 | GstE6 | 1 | |  |
| male | FBgn0129130 | LE | E | FBgn0063493 | GstE7 | 1 | |  |
| male | FBgn0129130 | LE | E | FBgn0063494 | GstE8 | 1 | |  |
| male | FBgn0129132 | HE | E |  | GstE9 | 1 | |  |
| male | FBgn0129133 | HE | E | FBgn0027835 | Dp1 | 1 | |  |
| male | FBgn0129142 | LE | E | FBgn0039527 | CG5639 | 1 | |  |
| male | FBgn0129147 | HE | E | FBgn0034919 | CG5569 | 1 | |  |
| male | FBgn0129152 | HE | E | FBgn0034921 | DCP1 | 1 | |  |
| male | FBgn0129158 | HE | E | FBgn0034940 | CG16787 | 1 | |  |
| male | FBgn0129163 | LE | E | FBgn0039525 | CG5646 | 1 | |  |
| male | FBgn0129188 | HE | E | FBgn0036565 | CG5235 | 1 | |  |
| male | FBgn0129197 | HE | E | FBgn0033019 | CG10395 | 1 | |  |
| male | FBgn0129199 | HE | E | --- | --- | 1 | |  |
| male | FBgn0129205 | HE | E | FBgn0033085 | CG15908 | 1 | |  |
| male | FBgn0129215 | HE | E | FBgn0042083 | CG3267 | 1 | |  |
| male | FBgn0129220 | LE | E | --- | --- | 1 | |  |
| male | FBgn0129241 | HE | E | FBgn0046114 | Gclm | 1 | |  |
| male | FBgn0129243 | HE | E | FBgn0033507 | CG12909 | 1 | |  |
| male | FBgn0129248 | LE | E | --- | --- | 1 | |  |
| male | FBgn0129249 | LE | E | --- | --- | 1 | |  |
| male | FBgn0129253 | LE | E | FBgn0033918 | CG8531 | 1 | |  |
| male | FBgn0129267 | HE | E | FBgn0010638 | Sec61beta | 1 | |  |
| male | FBgn0129268 | HE | E | FBgn0034259 | P32 | 1 | |  |
| male | FBgn0129271 | LE | E | FBgn0034253 | CG10936 | 1 | |  |
| male | FBgn0129282 | LE | E | FBgn0034497 | CG9090 | 1 | |  |
| male | FBgn0129286 | LE | E | FBgn0027529 | tapas | 1 | |  |
| male | FBgn0129287 | LE | E | --- | --- | 1 | |  |
| male | FBgn0129298 | LE | E | FBgn0003731 | Egfr | 1 | |  |
| male | FBgn0129303 | HE | E | FBgn0033903 | CG8323 | 1 | |  |
| male | FBgn0129320 | LE | E | FBgn0265191 | Glycogenin | 1 | |  |
| male | FBgn0129321 | LE | E | FBgn0265180 | CG44245 | 1 | |  |
| male | FBgn0129327 | LE | E | FBgn0037222 | CG14642 | 1 | |  |
| male | FBgn0129345 | HE | E | FBgn0033209 | CG12107 | 1 | |  |
| male | FBgn0129351 | HE | E | FBgn0037734 | trbd | 1 | |  |
| male | FBgn0129357 | HE | E | FBgn0013307 | Odc1 | 1 | |  |
| male | FBgn0129378 | HE | E | FBgn0034027 | CG8187 | 1 | |  |
| male | FBgn0129389 | HE | E | FBgn0026741 | mRpL18 | 1 | |  |
| male | FBgn0129403 | HE | E | FBgn0033600 | Cpr47Ec | 1 | |  |
| male | FBgn0129414 | LE | E | FBgn0050021 | metro | 1 | |  |
| male | FBgn0129421 | HE | E | FBgn0054026 | CG34026 | 1 | |  |
| male | FBgn0129445 | LE | E | FBgn0034529 | FAM21 | 1 | |  |
| male | FBgn0129450 | HE | E | FBgn0034535 | CG11110 | 1 | |  |
| male | FBgn0129462 | LE | E | FBgn0034554 | CG15227 | 1 | |  |
| male | FBgn0129469 | HE | E | FBgn0034564 | CG9344 | 1 | |  |
| male | FBgn0129477 | LE | E | FBgn0053349 | ppk25 | 1 | |  |
| male | FBgn0129479 | HE | E | FBgn0033241 | CG2915 | 1 | |  |
| male | FBgn0129490 | LE | E | FBgn0259211 | grh | 1 | |  |
| male | FBgn0129491 | LE | E | FBgn0015522 | olf186-M | 1 | |  |
| male | FBgn0129506 | HE | E | FBgn0035087 | CG2765 | 1 | |  |
| male | FBgn0129507 | HE | E | FBgn0027599 | CG2790 | 1 | |  |
| male | FBgn0129509 | HE | E | FBgn0035083 | Tina-1 | 1 | |  |
| male | FBgn0129549 | LE | E | FBgn0034391 | CG15080 | 1 | |  |
| male | FBgn0129552 | LE | E | FBgn0259145 | CG42260 | 1 | |  |
| male | FBgn0129557 | HE | E | FBgn0050259 | CG30259 | 1 | |  |
| male | FBgn0129563 | HE | E | FBgn0050196 | CG30196 | 1 | |  |
| male | FBgn0129567 | HE | E | FBgn0050195 | CG30195 | 1 | |  |
| male | FBgn0129573 | HE | E | FBgn0028424 | JhI-26 | 1 | |  |
| male | FBgn0129576 | LE | E | FBgn0028622 | qsm | 1 | |  |
| male | FBgn0129587 | HE | E | FBgn0010551 | Phb2 | 1 | |  |
| male | FBgn0129620 | LE | E | FBgn0039736 | CG7912 | 1 | |  |
| male | FBgn0129630 | HE | E | FBgn0039732 | CG15525 | 1 | |  |
| male | FBgn0129650 | HE | E | --- | --- | 1 | |  |
| male | FBgn0129673 | LE | E | FBgn0085405 | CG34376 | 1 | |  |
| male | FBgn0129677 | LE | E | --- | --- | 1 | |  |
| male | FBgn0129680 | LE | E | --- | --- | 1 | |  |
| male | FBgn0129681 | LE | E | --- | --- | 1 | |  |
| male | FBgn0129685 | LE | E | --- | --- | 1 | |  |
| male | FBgn0129698 | HE | E | FBgn0039259 | CG11781 | 1 | |  |
| male | FBgn0129700 | HE | E | FBgn0039258 | beta4GalT7 | 1 | |  |
| male | FBgn0129738 | HE | E | FBgn0037340 | CG14671 | 1 | |  |
| male | FBgn0129741 | LE | E | FBgn0010877 | l(3)05822 | 1 | |  |
| male | FBgn0129760 | HE | E | FBgn0038715 | CG7333 | 1 | |  |
| male | FBgn0129760 | HE | E | FBgn0038719 | CG16727 | 1 | |  |
| male | FBgn0129787 | HE | E | FBgn0262512 | Vha14-1 | 1 | |  |
| male | FBgn0129802 | HE | E | FBgn0058045 | CG40045 | 1 | |  |
| male | FBgn0129808 | HE | E | FBgn0037676 | CG8861 | 1 | |  |
| male | FBgn0129816 | HE | E | FBgn0031483 | CG9641 | 1 | |  |
| male | FBgn0129822 | HE | E | FBgn0050354 | UQCR-11L | 1 | |  |
| male | FBgn0129822 | HE | E | FBgn0260008 | UQCR-11 | 1 | |  |
| male | FBgn0129828 | HE | E | --- | --- | 1 | |  |
| male | FBgn0129845 | LE | E | FBgn0038714 | Cpr92A | 1 | |  |
| male | FBgn0129860 | LE | E | FBgn0037338 | Snm1 | 1 | |  |
| male | FBgn0129923 | LE | E | FBgn0261509 | haf | 1 | |  |
| male | FBgn0129924 | LE | E | --- | --- | 1 | |  |
| male | FBgn0129974 | HE | E | FBgn0035911 | CG6638 | 1 | |  |
| male | FBgn0129985 | HE | E | FBgn0035917 | Zasp66 | 1 | |  |
| male | FBgn0129986 | HE | E | FBgn0035917 | Zasp66 | 1 | |  |
| male | FBgn0129994 | LE | E | --- | --- | 1 | |  |
| male | FBgn0130051 | HE | E | FBgn0037327 | PEK | 1 | |  |
| male | FBgn0130070 | HE | E | ------ | ------ | 1 | |  |
| male | FBgn0130080 | LE | E | FBgn0001311 | kkv | 1 | |  |
| male | FBgn0130082 | HE | E | FBgn0004638 | drk | 1 | |  |
| male | FBgn0130102 | HE | E | FBgn0034162 | CG6426 | 1 | |  |
| male | FBgn0130109 | HE | E | FBgn0034168 | CG15614 | 1 | |  |
| male | FBgn0130111 | HE | E | FBgn0085220 | CG34191 | 1 | |  |
| male | FBgn0130113 | HE | E | FBgn0004055 | uzip | 1 | |  |
| male | FBgn0130116 | HE | E | --- | --- | 1 | |  |
| male | FBgn0130122 | HE | E | FBgn0037637 | IscU | 1 | |  |
| male | FBgn0130123 | HE | E | FBgn0037312 | CG11999 | 1 | |  |
| male | FBgn0130129 | LE | E | FBgn0002643 | mam | 1 | |  |
| male | FBgn0130138 | HE | E | FBgn0019928 | Ser8 | 1 | |  |
| male | FBgn0130162 | LE | E | FBgn0034151 | CG15617 | 1 | |  |
| male | FBgn0130196 | HE | E | FBgn0034645 | ND-B12 | 1 | |  |
| male | FBgn0130205 | LE | E | FBgn0034483 | CG16894 | 1 | |  |
| male | FBgn0130218 | LE | E | FBgn0046879 | Obp56c | 1 | |  |
| male | FBgn0130219 | HE | E | FBgn0046880 | Obp56b | 1 | |  |
| male | FBgn0130221 | LE | E | FBgn0259212 | cno | 1 | |  |
| male | FBgn0130236 | HE | E | FBgn0265177 | CG44242 | 1 | |  |
| male | FBgn0130241 | LE | E | --- | --- | 1 | |  |
| male | FBgn0130277 | HE | E | FBgn0037292 | plh | 1 | |  |
| male | FBgn0130281 | HE | E | FBgn0050481 | mRpL53 | 1 | |  |
| male | FBgn0130282 | HE | E | FBgn0053155 | CG33155 | 1 | |  |
| male | FBgn0130284 | HE | E | FBgn0275436 | PheRS-m | 1 | |  |
| male | FBgn0130291 | HE | E | FBgn0033891 | CG8067 | 1 | |  |
| male | FBgn0130310 | HE | E | FBgn0000504 | dsx | 1 | |  |
| male | FBgn0130313 | HE | E | FBgn0033437 | CG12926 | 1 | |  |
| male | FBgn0130349 | HE | E | FBgn0262736 | Vha16-1 | 1 | |  |
| male | FBgn0130359 | HE | E | FBgn0033079 | Fmo-2 | 1 | |  |
| male | FBgn0130373 | HE | E | FBgn0034909 | CG4797 | 1 | |  |
| male | FBgn0130391 | HE | E | FBgn0050338 | CG30338 | 1 | |  |
| male | FBgn0130398 | HE | E | FBgn0033443 | CG1698 | 1 | |  |
| male | FBgn0130420 | LE | E | FBgn0003067 | Pepck | 1 | |  |
| male | FBgn0130435 | HE | E | FBgn0033708 | CG8850 | 1 | |  |
| male | FBgn0130444 | HE | E | FBgn0053506 | CG33506 | 1 | |  |
| male | FBgn0130447 | HE | E | FBgn0050290 | Ppcdc | 1 | |  |
| male | FBgn0130451 | LE | E | FBgn0015295 | Shark | 1 | |  |
| male | FBgn0130459 | HE | E | FBgn0026378 | Rep | 1 | |  |
| male | FBgn0130465 | HE | E | FBgn0034460 | CG18367 | 1 | |  |
| male | FBgn0130478 | LE | E | FBgn0034476 | Toll-7 | 1 | |  |
| male | FBgn0130485 | HE | E | --- | --- | 1 | |  |
| male | FBgn0130490 | HE | E | FBgn0034485 | CG11099 | 1 | |  |
| male | FBgn0130509 | LE | E | FBgn0003009 | ord | 1 | |  |
| male | FBgn0130517 | LE | E | FBgn0265082 | Cdep | 1 | |  |
| male | FBgn0130519 | LE | E | --- | --- | 1 | |  |
| male | FBgn0130528 | HE | E | FBgn0263316 | Mrp4 | 1 | |  |
| male | FBgn0130534 | LE | E | FBgn0024232 | gprs | 1 | |  |
| male | FBgn0130554 | LE | E | FBgn0005695 | gcl | 1 | |  |
| male | FBgn0130555 | LE | E | FBgn0027585 | CG8740 | 1 | |  |
| male | FBgn0130558 | LE | E | FBgn0011286 | RyR | 1 | |  |
| male | FBgn0130563 | HE | E | --- | --- | 1 | |  |
| male | FBgn0130582 | HE | E | --- | --- | 1 | |  |
| male | FBgn0130610 | HE | E | FBgn0259725 | CG42379 | 1 | |  |
| male | FBgn0130621 | HE | E | --- | --- | 1 | |  |
| male | FBgn0130624 | HE | E | FBgn0003257 | r-l | 1 | |  |
| male | FBgn0130650 | HE | E | --- | --- | 1 | |  |
| male | FBgn0130659 | HE | E | FBgn0038774 | CG5023 | 1 | |  |
| male | FBgn0130677 | LE | E | --- | --- | 1 | |  |
| male | FBgn0130695 | LE | E | FBgn0045759 | bin | 1 | |  |
| male | FBgn0130731 | LE | E | FBgn0038753 | CG4459 | 1 | |  |
| male | FBgn0130772 | LE | E | --- | --- | 1 | |  |
| male | FBgn0130816 | LE | E | FBgn0035217 | FucTD | 1 | |  |
| male | FBgn0130847 | LE | E | FBgn0031446 | CG15398 | 1 | |  |
| male | FBgn0130885 | HE | E | FBgn0053510 | CG33510 | 1 | |  |
| male | FBgn0130886 | HE | E | FBgn0053511 | CG33511 | 1 | |  |
| male | FBgn0130903 | HE | E | FBgn0027095 | Manf | 1 | |  |
| male | FBgn0130920 | HE | E | --- | --- | 1 | |  |
| male | FBgn0130929 | LE | E | --- | --- | 1 | |  |
| male | FBgn0130930 | HE | E | FBgn0038387 | blp | 1 | |  |
| male | FBgn0130945 | LE | E | --- | --- | 1 | |  |
| male | FBgn0130952 | HE | E | FBgn0038390 | Rbf2 | 1 | |  |
| male | FBgn0130964 | LE | E | --- | --- | 1 | |  |
| male | FBgn0130972 | LE | E | --- | --- | 1 | |  |
| male | FBgn0130984 | HE | E | --- | --- | 1 | |  |
| male | FBgn0130990 | HE | E | FBgn0260743 | GC1 | 1 | |  |
| male | FBgn0131019 | LE | E | FBgn0039137 | CG13604 | 1 | |  |
| male | FBgn0131044 | HE | E | FBgn0039131 | CG12268 | 1 | |  |
| male | FBgn0131070 | HE | E | FBgn0039902 | Zip102B | 1 | |  |
| male | FBgn0131078 | HE | E | FBgn0052850 | CG32850 | 1 | |  |
| male | FBgn0131099 | HE | E | FBgn0032036 | CG13384 | 1 | |  |
| male | FBgn0131135 | LE | E | --- | --- | 1 | |  |
| male | FBgn0131158 | HE | E | FBgn0031373 | CG15358 | 1 | |  |
| male | FBgn0131176 | LE/HE | E | --- | --- | 1 | |  |
| male | FBgn0131181 | HE | E | --- | --- | 1 | |  |
| male | FBgn0131183 | HE | E | --- | --- | 1 | |  |
| male | FBgn0131214 | LE | E | FBgn0038202 | CG12402 | 1 | |  |
| male | FBgn0131216 | LE | E | --- | --- | 1 | |  |
| male | FBgn0131229 | LE | E | FBgn0004387 | Klp98A | 1 | |  |
| male | FBgn0131237 | LE | E | FBgn0051617 | His1:CG31617 | 1 | |  |
| male | FBgn0131245 | LE | E | --- | --- | 1 | |  |
| male | FBgn0131246 | LE | E | FBgn0039523 | CG12885 | 1 | |  |
| male | FBgn0131289 | HE | E | --- | --- | 1 | |  |
| male | FBgn0131306 | HE | E | --- | --- | 1 | |  |
| male | FBgn0131309 | HE | E | FBgn0004644 | hh | 1 | |  |
| male | FBgn0131329 | HE | E | --- | --- | 1 | |  |
| male | FBgn0131343 | LE | E | ------ | ------ | 1 | |  |
| male | FBgn0131348 | LE | E | --- | --- | 1 | |  |
| male | FBgn0131356 | LE | E | FBgn0032665 | CG15152 | 1 | |  |
| male | FBgn0131398 | HE | E | FBgn0024811 | Crk | 1 | |  |
| male | FBgn0131403 | HE | E | --- | --- | 1 | |  |
| male | FBgn0131408 | HE | E | FBgn0013749 | Arf102F | 1 | |  |
| male | FBgn0131410 | HE | E | FBgn0053653 | Cadps | 1 | |  |
| male | FBgn0131436 | LE | E | FBgn0037899 | RpL24-like | 1 | |  |
| male | FBgn0131445 | LE | E | FBgn0250819 | CG33521 | 1 | |  |
| male | FBgn0131447 | HE | E | FBgn0263112 | Mitf | 1 | |  |
| male | FBgn0131461 | LE | E | FBgn0039937 | fd102C | 1 | |  |
| male | FBgn0131467 | HE | E | --- | --- | 1 | |  |
| male | FBgn0131471 | HE | E | FBgn0022361 | Pur-alpha | 1 | |  |
| male | FBgn0131491 | HE | E | FBgn0031041 | CG12788 | 1 | |  |
| male | FBgn0131499 | HE | E | FBgn0015379 | dod | 1 | |  |
| male | FBgn0131514 | HE | E | FBgn0029866 | CG3842 | 1 | |  |
| male | FBgn0131515 | HE | E | FBgn0029867 | CG3847 | 1 | |  |
| male | FBgn0131519 | HE | E | FBgn0029709 | CHOp24 | 1 | |  |
| male | FBgn0131532 | LE | E | FBgn0027279 | l(1)G0196 | 1 | |  |
| male | FBgn0131550 | LE | E | FBgn0030004 | CG10958 | 1 | |  |
| male | FBgn0131556 | LE | E | FBgn0030012 | CG18262 | 1 | |  |
| male | FBgn0131557 | LE | E | FBgn0030013 | GIIIspla2 | 1 | |  |
| male | FBgn0131570 | HE | E | FBgn0030460 | CG2453 | 1 | |  |
| male | FBgn0131576 | HE | E | FBgn0029849 | Efr | 1 | |  |
| male | FBgn0131577 | HE | E | FBgn0029849 | Efr | 1 | |  |
| male | FBgn0131590 | LE | E | --- | --- | 1 | |  |
| male | FBgn0131598 | HE | E | FBgn0038446 | CG14903 | 1 | |  |
| male | FBgn0131599 | HE | E | FBgn0000221 | brn | 1 | |  |
| male | FBgn0131617 | HE | E | FBgn0004657 | mys | 1 | |  |
| male | FBgn0131662 | LE | E | FBgn0004102 | oc | 1 | |  |
| male | FBgn0131668 | HE | E | FBgn0044046 | Ilp7 | 1 | |  |
| male | FBgn0131669 | LE | E | FBgn0023215 | Mnt | 1 | |  |
| male | FBgn0131694 | HE | E | FBgn0029888 | ND-ASHI | 1 | |  |
| male | FBgn0131701 | HE | E | FBgn0026089 | CG14817 | 1 | |  |
| male | FBgn0131705 | LE | E | FBgn0040392 | CG14050 | 1 | |  |
| male | FBgn0131707 | LE | E | FBgn0023526 | CG2865 | 1 | |  |
| male | FBgn0131708 | HE | E | --- | --- | 1 | |  |
| male | FBgn0131709 | LE | E | FBgn0023529 | CG2918 | 1 | |  |
| male | FBgn0131714 | HE | E | FBgn0029942 | CG2059 | 1 | |  |
| male | FBgn0131721 | LE | E | FBgn0034122 | CG15711 | 1 | |  |
| male | FBgn0131737 | LE | E | FBgn0001565 | Hlc | 1 | |  |
| male | FBgn0131743 | HE | E | FBgn0030584 | CG14407 | 1 | |  |
| male | FBgn0131749 | HE | E | --- | --- | 1 | |  |
| male | FBgn0131752 | HE | E | FBgn0023540 | CG3630 | 1 | |  |
| male | FBgn0131768 | HE | E | FBgn0030611 | CG15027 | 1 | |  |
| male | FBgn0131769 | HE | E | FBgn0030610 | CG9065 | 1 | |  |
| male | FBgn0131773 | LE | E | FBgn0004456 | mew | 1 | |  |
| male | FBgn0131781 | HE | E | FBgn0031091 | Phf7 | 1 | |  |
| male | FBgn0131793 | HE | E | --- | --- | 1 | |  |
| male | FBgn0131805 | HE | E | FBgn0029994 | CG2254 | 1 | |  |
| male | FBgn0131807 | HE | E | FBgn0040087 | p115 | 1 | |  |
| male | FBgn0131816 | HE | E | --- | --- | 1 | |  |
| male | FBgn0131828 | LE | E | FBgn0000042 | Act5C | 1 | |  |
| male | FBgn0131828 | LE | E | FBgn0000043 | Act42A | 1 | |  |
| male | FBgn0131832 | LE | E | FBgn0029824 | CG3726 | 1 | |  |
| male | FBgn0131838 | LE | E | FBgn0029830 | Grip | 1 | |  |
| male | FBgn0131839 | LE | E | FBgn0029831 | CG5966 | 1 | |  |
| male | FBgn0131842 | HE | E | FBgn0030329 | prtp | 1 | |  |
| male | FBgn0131847 | LE | E | FBgn0004655 | wapl | 1 | |  |
| male | FBgn0131848 | LE | E | --- | --- | 1 | |  |
| male | FBgn0131859 | LE | E | FBgn0029719 | CG15473 | 1 | |  |
| male | FBgn0131861 | LE | E | FBgn0025625 | Sik2 | 1 | |  |
| male | FBgn0131866 | LE | E | FBgn0000667 | Actn | 1 | |  |
| male | FBgn0131891 | HE | E | FBgn0030676 | CG12379 | 1 | |  |
| male | FBgn0131909 | HE | E | FBgn0004924 | Top1 | 1 | |  |
| male | FBgn0131918 | HE | E | --- | --- | 1 | |  |
| male | FBgn0131923 | HE | E | FBgn0029687 | Vap-33A | 1 | |  |
| male | FBgn0131966 | HE | E | --- | --- | 1 | |  |
| male | FBgn0131968 | HE | E | FBgn0030187 | Ipod | 1 | |  |
| male | FBgn0131969 | HE | E | --- | --- | 1 | |  |
| male | FBgn0131974 | HE | E | FBgn0025637 | SkpA | 1 | |  |
| male | FBgn0131985 | HE | E | FBgn0026088 | CG14818 | 1 | |  |
| male | FBgn0131991 | LE | E | FBgn0024897 | b6 | 1 | |  |
| male | FBgn0131995 | HE | E | FBgn0031514 | CG3332 | 1 | |  |
| male | FBgn0132000 | HE | E | FBgn0030675 | CG8191 | 1 | |  |
| male | FBgn0132001 | HE | E | FBgn0026666 | MagR | 1 | |  |
| male | FBgn0132010 | LE | E | FBgn0003053 | peb | 1 | |  |
| male | FBgn0132013 | HE | E | FBgn0030882 | GSS | 1 | |  |
| male | FBgn0132013 | HE | E | FBgn0052495 | CG32495 | 1 | |  |
| male | FBgn0132031 | HE | E | FBgn0052667 | ssp7 | 1 | |  |
| male | FBgn0132039 | LE | E | --- | --- | 1 | |  |
| male | FBgn0132048 | LE | E | FBgn0085350 | inaF-C | 1 | |  |
| male | FBgn0132055 | LE | E | --- | --- | 1 | |  |
| male | FBgn0132069 | HE | E | FBgn0024332 | Mcm3 | 1 | |  |
| male | FBgn0132073 | LE | E | --- | --- | 1 | |  |
| male | FBgn0132074 | LE | E | FBgn0029761 | SK | 1 | |  |
| male | FBgn0132098 | HE | E | FBgn0030612 | CG5599 | 1 | |  |
| male | FBgn0132115 | HE | E | --- | --- | 1 | |  |
| male | FBgn0132116 | LE | E | FBgn0030245 | CG1637 | 1 | |  |
| male | FBgn0132117 | HE | E | --- | --- | 1 | |  |
| male | FBgn0132120 | HE | E | FBgn0030583 | CG14410 | 1 | |  |
| male | FBgn0132122 | HE | E | FBgn0040907 | mRpL33 | 1 | |  |
| male | FBgn0132135 | HE | E | FBgn0039930 | CG11077 | 1 | |  |
| male | FBgn0132140 | LE | E | FBgn0003028 | ovo | 1 | |  |
| male | FBgn0132141 | HE | E | FBgn0261284 | bou | 1 | |  |
| male | FBgn0132145 | LE | E | --- | --- | 1 | |  |
| male | FBgn0132162 | HE | E | FBgn0014411 | Vps26 | 1 | |  |
| male | FBgn0132170 | HE | E | FBgn0025815 | Mcm6 | 1 | |  |
| male | FBgn0132177 | LE | E | FBgn0261446 | CG13377 | 1 | |  |
| male | FBgn0132183 | HE | E | FBgn0030976 | CG7378 | 1 | |  |
| male | FBgn0132185 | HE | E | FBgn0264090 | CG43759 | 1 | |  |
| male | FBgn0132191 | HE | E | FBgn0030809 | Ubr1 | 1 | |  |
| male | FBgn0132193 | HE | E | FBgn0030799 | CG4872 | 1 | |  |
| male | FBgn0132196 | LE | E | FBgn0027375 | RecQ5 | 1 | |  |
| male | FBgn0132198 | LE | E | FBgn0025864 | Crag | 1 | |  |
| male | FBgn0132211 | HE | E | --- | --- | 1 | |  |
| male | FBgn0132227 | HE | E | FBgn0029980 | CG10778 | 1 | |  |
| male | FBgn0132232 | LE | E | FBgn0030685 | Graf | 1 | |  |
| male | FBgn0132242 | HE | E | FBgn0043001 | Chrac-16 | 1 | |  |
| male | FBgn0132256 | HE | E | FBgn0029854 | CG3566 | 1 | |  |
| male | FBgn0132285 | LE | E | FBgn0030403 | CG1824 | 1 | |  |
| male | FBgn0132311 | LE | E | --- | --- | 1 | |  |
| male | FBgn0132343 | HE | E | FBgn0030791 | CG9132 | 1 | |  |
| male | FBgn0132346 | HE | E | FBgn0011742 | Arp2 | 1 | |  |
| male | FBgn0132351 | LE | E | FBgn0030528 | CG11095 | 1 | |  |
| male | FBgn0132365 | LE | E | --- | --- | 1 | |  |
| male | FBgn0132375 | LE | E | FBgn0033807 | AQP | 1 | |  |
| male | FBgn0132378 | HE | E | FBgn0029778 | RhoGAP5A | 1 | |  |
| male | FBgn0132401 | LE | E | --- | --- | 1 | |  |
| male | FBgn0132418 | HE | E | --- | --- | 1 | |  |
| male | FBgn0132434 | LE | E | FBgn0024179 | wit | 1 | |  |
| male | FBgn0132441 | LE | E | --- | --- | 1 | |  |
| male | FBgn0132444 | HE | E | --- | --- | 1 | |  |
| male | FBgn0132450 | HE | E | --- | --- | 1 | |  |
| male | FBgn0132456 | LE | E | --- | --- | 1 | |  |
| male | FBgn0132471 | HE | E | FBgn0031378 | CG15362 | 1 | |  |
| male | FBgn0132483 | LE | E | FBgn0032587 | CG5953 | 1 | |  |
| male | FBgn0132487 | HE | E | FBgn0025678 | CaBP1 | 1 | |  |
| male | FBgn0132529 | LE | E | FBgn0032138 | CG4364 | 1 | |  |
| male | FBgn0132539 | HE | E | FBgn0032082 | CG18088 | 1 | |  |
| male | FBgn0132550 | LE | E | FBgn0051728 | CG31728 | 1 | |  |
| male | FBgn0132576 | HE | E | --- | --- | 1 | |  |
| male | FBgn0132577 | HE | E | FBgn0032079 | CG31886 | 1 | |  |
| male | FBgn0132581 | HE | E | FBgn0283531 | Duox | 1 | |  |
| male | FBgn0132592 | HE | E | FBgn0015393 | hoip | 1 | |  |
| male | FBgn0132596 | HE | E | FBgn0032140 | CG13117 | 1 | |  |
| male | FBgn0132629 | LE | E | --- | --- | 1 | |  |
| male | FBgn0132659 | LE | E | FBgn0051665 | wry | 1 | |  |
| male | FBgn0132661 | LE | E | --- | --- | 1 | |  |
| male | FBgn0132662 | LE | E | FBgn0053673 | CG33673 | 1 | |  |
| male | FBgn0132671 | LE | E | --- | --- | 1 | |  |
| male | FBgn0132725 | LE | E | FBgn0034768 | Obp58b | 1 | |  |
