# Supplementary material for: Hawaiian picture‐winged Drosophila exhibit adaptive population divergence along a narrow climatic gradient on Hawaii Island
Source: Ecol Evol. 2019 Feb 18;9(5):2436–48. doi: 10.1002/ece3.4844 (PMC6405895; doi:10.1002/ece3.4844)
Supplement: Supplementary file 2 [file ECE3-9-2436-s002.docx]

| GO Category | Overenriched GO-Slim Term | [*Dmel*  #](http://pantherdb.org/tools/compareToRefList.jsp?sortOrder=2&sortList=Drosophila%20melanogaster) | DEG # | | [Expected DEG #](http://pantherdb.org/tools/compareToRefList.jsp?sortOrder=2&sortList=Client%20Text%20Box%20Input&sortField=exp) | | [Fold Enrichment](http://pantherdb.org/tools/compareToRefList.jsp?sortOrder=2&sortList=Client%20Text%20Box%20Input&sortField=foldEnrich) | | [+/-](http://pantherdb.org/tools/compareToRefList.jsp?sortOrder=1&sortList=Client%20Text%20Box%20Input&sortField=rep) | | [Raw P value](http://pantherdb.org/tools/compareToRefList.jsp?sortOrder=1&sortList=Client%20Text%20Box%20Input&sortField=pval) | | [FDR](http://pantherdb.org/tools/compareToRefList.jsp?sortOrder=2&sortList=Client%20Text%20Box%20Input&sortField=fdr) |
| --- | --- | --- | --- | --- | --- | --- | --- | --- | --- | --- | --- | --- | --- |
| **High Elevation Population** | |  |  |  | |  | |  | |  | |  | |
| BP | [carbohydrate transport](http://pantherdb.org/panther/category.do?categoryAcc=GO:0008643) | [30](http://pantherdb.org/tools/gxIdsList.do?acc=GO:0008643&reflist=1) | [9](http://pantherdb.org/tools/gxIdsList.do?acc=GO:0008643&list=gene_symbols_HE&organism=Drosophila%20melanogaster) | 1.72 | | 5.22 | | + | | 1.91E-04 | | 4.31E-03 | |
|  | [transport](http://pantherdb.org/panther/category.do?categoryAcc=GO:0006810) | [1012](http://pantherdb.org/tools/gxIdsList.do?acc=GO:0006810&reflist=1) | [86](http://pantherdb.org/tools/gxIdsList.do?acc=GO:0006810&list=gene_symbols_HE&organism=Drosophila%20melanogaster) | 58.11 | | 1.48 | | + | | 5.10E-04 | | 9.60E-03 | |
|  | [localization](http://pantherdb.org/panther/category.do?categoryAcc=GO:0051179) | [1108](http://pantherdb.org/tools/gxIdsList.do?acc=GO:0051179&reflist=1) | [92](http://pantherdb.org/tools/gxIdsList.do?acc=GO:0051179&list=gene_symbols_HE&organism=Drosophila%20melanogaster) | 63.63 | | 1.45 | | + | | 6.66E-04 | | 1.08E-02 | |
|  | [oxidative phosphorylation](http://pantherdb.org/panther/category.do?categoryAcc=GO:0006119) | [56](http://pantherdb.org/tools/gxIdsList.do?acc=GO:0006119&reflist=1) | [13](http://pantherdb.org/tools/gxIdsList.do?acc=GO:0006119&list=gene_symbols_HE&organism=Drosophila%20melanogaster) | 3.22 | | 4.04 | | + | | 7.40E-05 | | 1.86E-03 | |
|  | [generation of precursor metabolites and energy](http://pantherdb.org/panther/category.do?categoryAcc=GO:0006091) | [186](http://pantherdb.org/tools/gxIdsList.do?acc=GO:0006091&reflist=1) | [27](http://pantherdb.org/tools/gxIdsList.do?acc=GO:0006091&list=gene_symbols_HE&organism=Drosophila%20melanogaster) | 10.68 | | 2.53 | | + | | 4.14E-05 | | 1.34E-03 | |
|  | [metabolic process](http://pantherdb.org/panther/category.do?categoryAcc=GO:0008152) | [3558](http://pantherdb.org/tools/gxIdsList.do?acc=GO:0008152&reflist=1) | [285](http://pantherdb.org/tools/gxIdsList.do?acc=GO:0008152&list=gene_symbols_HE&organism=Drosophila%20melanogaster) | 204.32 | | 1.39 | | + | | 9.01E-10 | | 2.04E-07 | |
|  | [sulfur compound metabolic process](http://pantherdb.org/panther/category.do?categoryAcc=GO:0006790) | [77](http://pantherdb.org/tools/gxIdsList.do?acc=GO:0006790&reflist=1) | [16](http://pantherdb.org/tools/gxIdsList.do?acc=GO:0006790&list=gene_symbols_HE&organism=Drosophila%20melanogaster) | 4.42 | | 3.62 | | + | | 3.68E-05 | | 1.39E-03 | |
|  | [respiratory electron transport chain](http://pantherdb.org/panther/category.do?categoryAcc=GO:0022904) | [128](http://pantherdb.org/tools/gxIdsList.do?acc=GO:0022904&reflist=1) | [23](http://pantherdb.org/tools/gxIdsList.do?acc=GO:0022904&list=gene_symbols_HE&organism=Drosophila%20melanogaster) | 7.35 | | 3.13 | | + | | 6.73E-06 | | 3.04E-04 | |
|  | [mitochondrion organization](http://pantherdb.org/panther/category.do?categoryAcc=GO:0007005) | [92](http://pantherdb.org/tools/gxIdsList.do?acc=GO:0007005&reflist=1) | [14](http://pantherdb.org/tools/gxIdsList.do?acc=GO:0007005&list=gene_symbols_HE&organism=Drosophila%20melanogaster) | 5.28 | | 2.65 | | + | | 1.75E-03 | | 2.33E-02 | |
|  | [cellular component organization or biogenesis](http://pantherdb.org/panther/category.do?categoryAcc=GO:0071840) | [1089](http://pantherdb.org/tools/gxIdsList.do?acc=GO:0071840&reflist=1) | [91](http://pantherdb.org/tools/gxIdsList.do?acc=GO:0071840&list=gene_symbols_HE&organism=Drosophila%20melanogaster) | 62.54 | | 1.46 | | + | | 6.03E-04 | | 1.05E-02 | |
|  | [rRNA metabolic process](http://pantherdb.org/panther/category.do?categoryAcc=GO:0016072) | [104](http://pantherdb.org/tools/gxIdsList.do?acc=GO:0016072&reflist=1) | [15](http://pantherdb.org/tools/gxIdsList.do?acc=GO:0016072&list=gene_symbols_HE&organism=Drosophila%20melanogaster) | 5.97 | | 2.51 | | + | | 1.96E-03 | | 2.46E-02 | |
|  | [nucleobase-containing compound metabolic process](http://pantherdb.org/panther/category.do?categoryAcc=GO:0006139) | [1589](http://pantherdb.org/tools/gxIdsList.do?acc=GO:0006139&reflist=1) | [118](http://pantherdb.org/tools/gxIdsList.do?acc=GO:0006139&list=gene_symbols_HE&organism=Drosophila%20melanogaster) | 91.25 | | 1.29 | | + | | 5.28E-03 | | 4.97E-02 | |
|  | [primary metabolic process](http://pantherdb.org/panther/category.do?categoryAcc=GO:0044238) | [2916](http://pantherdb.org/tools/gxIdsList.do?acc=GO:0044238&reflist=1) | [235](http://pantherdb.org/tools/gxIdsList.do?acc=GO:0044238&list=gene_symbols_HE&organism=Drosophila%20melanogaster) | 167.45 | | 1.4 | | + | | 4.23E-08 | | 3.19E-06 | |
|  | [cellular amino acid metabolic process](http://pantherdb.org/panther/category.do?categoryAcc=GO:0006520) | [191](http://pantherdb.org/tools/gxIdsList.do?acc=GO:0006520&reflist=1) | [24](http://pantherdb.org/tools/gxIdsList.do?acc=GO:0006520&list=gene_symbols_HE&organism=Drosophila%20melanogaster) | 10.97 | | 2.19 | | + | | 7.19E-04 | | 1.08E-02 | |
|  | [anion transport](http://pantherdb.org/panther/category.do?categoryAcc=GO:0006820) | [168](http://pantherdb.org/tools/gxIdsList.do?acc=GO:0006820&reflist=1) | [21](http://pantherdb.org/tools/gxIdsList.do?acc=GO:0006820&list=gene_symbols_HE&organism=Drosophila%20melanogaster) | 9.65 | | 2.18 | | + | | 1.74E-03 | | 2.46E-02 | |
|  | [cellular component biogenesis](http://pantherdb.org/panther/category.do?categoryAcc=GO:0044085) | [463](http://pantherdb.org/tools/gxIdsList.do?acc=GO:0044085&reflist=1) | [47](http://pantherdb.org/tools/gxIdsList.do?acc=GO:0044085&list=gene_symbols_HE&organism=Drosophila%20melanogaster) | 26.59 | | 1.77 | | + | | 4.37E-04 | | 8.98E-03 | |
|  | [nitrogen compound metabolic process](http://pantherdb.org/panther/category.do?categoryAcc=GO:0006807) | [1550](http://pantherdb.org/tools/gxIdsList.do?acc=GO:0006807&reflist=1) | [128](http://pantherdb.org/tools/gxIdsList.do?acc=GO:0006807&list=gene_symbols_HE&organism=Drosophila%20melanogaster) | 89.01 | | 1.44 | | + | | 5.73E-05 | | 1.62E-03 | |
|  | [protein metabolic process](http://pantherdb.org/panther/category.do?categoryAcc=GO:0019538) | [1047](http://pantherdb.org/tools/gxIdsList.do?acc=GO:0019538&reflist=1) | [85](http://pantherdb.org/tools/gxIdsList.do?acc=GO:0019538&list=gene_symbols_HE&organism=Drosophila%20melanogaster) | 60.12 | | 1.41 | | + | | 2.06E-03 | | 2.45E-02 | |
|  | [biosynthetic process](http://pantherdb.org/panther/category.do?categoryAcc=GO:0009058) | [1081](http://pantherdb.org/tools/gxIdsList.do?acc=GO:0009058&reflist=1) | [87](http://pantherdb.org/tools/gxIdsList.do?acc=GO:0009058&list=gene_symbols_HE&organism=Drosophila%20melanogaster) | 62.08 | | 1.4 | | + | | 2.38E-03 | | 2.69E-02 | |
|  | [cellular process](http://pantherdb.org/panther/category.do?categoryAcc=GO:0009987) | [4086](http://pantherdb.org/tools/gxIdsList.do?acc=GO:0009987&reflist=1) | [308](http://pantherdb.org/tools/gxIdsList.do?acc=GO:0009987&list=gene_symbols_HE&organism=Drosophila%20melanogaster) | 234.64 | | 1.31 | | + | | 6.85E-08 | | 3.87E-06 | |
|  | [neurological system process](http://pantherdb.org/panther/category.do?categoryAcc=GO:0050877) | [399](http://pantherdb.org/tools/gxIdsList.do?acc=GO:0050877&reflist=1) | [10](http://pantherdb.org/tools/gxIdsList.do?acc=GO:0050877&list=gene_symbols_HE&organism=Drosophila%20melanogaster) | 22.91 | | 0.44 | | - | | 5.26E-03 | | 5.17E-02 | |
|  | [single-multicellular organism process](http://pantherdb.org/panther/category.do?categoryAcc=GO:0044707) | [550](http://pantherdb.org/tools/gxIdsList.do?acc=GO:0044707&reflist=1) | [16](http://pantherdb.org/tools/gxIdsList.do?acc=GO:0044707&list=gene_symbols_HE&organism=Drosophila%20melanogaster) | 31.58 | | 0.51 | | - | | 3.28E-03 | | 3.53E-02 | |
|  | [multicellular organismal process](http://pantherdb.org/panther/category.do?categoryAcc=GO:0032501) | [574](http://pantherdb.org/tools/gxIdsList.do?acc=GO:0032501&reflist=1) | [17](http://pantherdb.org/tools/gxIdsList.do?acc=GO:0032501&list=gene_symbols_HE&organism=Drosophila%20melanogaster) | 32.96 | | 0.52 | | - | | 3.86E-03 | | 3.97E-02 | |
| MF    \|  \| \| --- \| \| | Top of Form  [carbohydrate transmembrane transporter activity](http://pantherdb.org/panther/category.do?categoryAcc=GO:0015144)  Bottom of Form | [24](http://pantherdb.org/tools/gxIdsList.do?acc=GO:0015144&reflist=1) | [61](http://pantherdb.org/tools/gxIdsList.do?acc=GO:0005215&list=Client%20Text%20Box%20Input&organism=Drosophila%20melanogaster) | 37.14 | | 5.07 | | + | | 1.14E-03 | | 2.33E-02 | |
|  | [transporter activity](http://pantherdb.org/panther/category.do?categoryAcc=GO:0005215) | [646](http://pantherdb.org/tools/gxIdsList.do?acc=GO:0005215&reflist=1) | [23](http://pantherdb.org/tools/gxIdsList.do?acc=GO:0008324&list=Client%20Text%20Box%20Input&organism=Drosophila%20melanogaster) | 10.29 | | 1.64 | | + | | 3.26E-04 | | 1.07E-02 | |
|  | [cation transmembrane transporter activity](http://pantherdb.org/panther/category.do?categoryAcc=GO:0008324) | [179](http://pantherdb.org/tools/gxIdsList.do?acc=GO:0008324&reflist=1) | [55](http://pantherdb.org/tools/gxIdsList.do?acc=GO:0022857&list=Client%20Text%20Box%20Input&organism=Drosophila%20melanogaster) | 32.03 | | 2.23 | | + | | 7.85E-04 | | 1.13E-05 | |
|  | [transmembrane transporter activity](http://pantherdb.org/panther/category.do?categoryAcc=GO:0022857) | [557](http://pantherdb.org/tools/gxIdsList.do?acc=GO:0022857&reflist=1) | [59](http://pantherdb.org/tools/gxIdsList.do?acc=GO:0016491&list=Client%20Text%20Box%20Input&organism=Drosophila%20melanogaster) | 26.97 | | 1.72 | | + | | 2.40E-04 | | 2.22E-05 | |
|  | [oxidoreductase activity](http://pantherdb.org/panther/category.do?categoryAcc=GO:0016491) | [469](http://pantherdb.org/tools/gxIdsList.do?acc=GO:0016491&reflist=1) | [230](http://pantherdb.org/tools/gxIdsList.do?acc=GO:0003824&list=Client%20Text%20Box%20Input&organism=Drosophila%20melanogaster) | 167.43 | | 2.19 | | + | | 1.27E-07 | | 6.25E-07 | |
|  | [catalytic activity](http://pantherdb.org/panther/category.do?categoryAcc=GO:0003824) | [2912](http://pantherdb.org/tools/gxIdsList.do?acc=GO:0003824&reflist=1) | [414](http://pantherdb.org/tools/gxIdsList.do?acc=UNCLASSIFIED&list=Client%20Text%20Box%20Input&organism=Drosophila%20melanogaster) | 497.93 | | 1.37 | | + | | 3.75E-07 | | 5.23E-03 | |
|    \| CC \| \| --- \| \| | [mitochondrial inner membrane](http://pantherdb.org/panther/category.do?categoryAcc=GO:0005743) | [106](http://pantherdb.org/tools/gxIdsList.do?acc=GO:0005743&reflist=1) | [16](http://pantherdb.org/tools/gxIdsList.do?acc=GO:0005743&list=gene_symbols_HE&organism=Drosophila%20melanogaster) | 6.09 | | 2.63 | | + | | 9.17E-04 | | 5.17E-03 | |
|  | [mitochondrion](http://pantherdb.org/panther/category.do?categoryAcc=GO:0005739) | [270](http://pantherdb.org/tools/gxIdsList.do?acc=GO:0005739&reflist=1) | [37](http://pantherdb.org/tools/gxIdsList.do?acc=GO:0005739&list=gene_symbols_HE&organism=Drosophila%20melanogaster) | 15.5 | | 2.39 | | + | | 4.99E-06 | | 4.42E-05 | |
|  | [organelle](http://pantherdb.org/panther/category.do?categoryAcc=GO:0043226) | [2272](http://pantherdb.org/tools/gxIdsList.do?acc=GO:0043226&reflist=1) | [190](http://pantherdb.org/tools/gxIdsList.do?acc=GO:0043226&list=gene_symbols_HE&organism=Drosophila%20melanogaster) | 130.47 | | 1.46 | | + | | 1.57E-07 | | 1.95E-06 | |
|  | [membrane](http://pantherdb.org/panther/category.do?categoryAcc=GO:0016020) | [1088](http://pantherdb.org/tools/gxIdsList.do?acc=GO:0016020&reflist=1) | [97](http://pantherdb.org/tools/gxIdsList.do?acc=GO:0016020&list=gene_symbols_HE&organism=Drosophila%20melanogaster) | 62.48 | | 1.55 | | + | | 4.09E-05 | | 2.82E-04 | |
|  | [endoplasmic reticulum](http://pantherdb.org/panther/category.do?categoryAcc=GO:0005783) | [251](http://pantherdb.org/tools/gxIdsList.do?acc=GO:0005783&reflist=1) | [36](http://pantherdb.org/tools/gxIdsList.do?acc=GO:0005783&list=gene_symbols_HE&organism=Drosophila%20melanogaster) | 14.41 | | 2.5 | | + | | 2.45E-06 | | 2.54E-05 | |
|  | [cytoplasm](http://pantherdb.org/panther/category.do?categoryAcc=GO:0005737) | [2010](http://pantherdb.org/tools/gxIdsList.do?acc=GO:0005737&reflist=1) | [196](http://pantherdb.org/tools/gxIdsList.do?acc=GO:0005737&list=gene_symbols_HE&organism=Drosophila%20melanogaster) | 115.42 | | 1.7 | | + | | 3.85E-13 | | 2.39E-11 | |
|  | [intracellular](http://pantherdb.org/panther/category.do?categoryAcc=GO:0005622) | [3160](http://pantherdb.org/tools/gxIdsList.do?acc=GO:0005622&reflist=1) | [267](http://pantherdb.org/tools/gxIdsList.do?acc=GO:0005622&list=gene_symbols_HE&organism=Drosophila%20melanogaster) | 181.46 | | 1.47 | | + | | 1.87E-11 | | 5.81E-10 | |
|  | [cell part](http://pantherdb.org/panther/category.do?categoryAcc=GO:0044464) | [3347](http://pantherdb.org/tools/gxIdsList.do?acc=GO:0044464&reflist=1) | [273](http://pantherdb.org/tools/gxIdsList.do?acc=GO:0044464&list=gene_symbols_HE&organism=Drosophila%20melanogaster) | 192.2 | | 1.42 | | + | | 4.16E-10 | | 6.45E-09 | |
|  | [protein complex](http://pantherdb.org/panther/category.do?categoryAcc=GO:0043234) | [1096](http://pantherdb.org/tools/gxIdsList.do?acc=GO:0043234&reflist=1) | [92](http://pantherdb.org/tools/gxIdsList.do?acc=GO:0043234&list=gene_symbols_HE&organism=Drosophila%20melanogaster) | 62.94 | | 1.46 | | + | | 4.88E-04 | | 3.02E-03 | |
|  | [macromolecular complex](http://pantherdb.org/panther/category.do?categoryAcc=GO:0032991) | [1372](http://pantherdb.org/tools/gxIdsList.do?acc=GO:0032991&reflist=1) | [120](http://pantherdb.org/tools/gxIdsList.do?acc=GO:0032991&list=gene_symbols_HE&organism=Drosophila%20melanogaster) | 78.79 | | 1.52 | | + | | 9.67E-06 | | 7.49E-05 | |
|  |  |  |  |  | |  | |  | |  | |  | |
| **Low Elevation** | | | | | | | | | | | | | |
|    \| BP \| \| --- \| \| | [negative regulation of apoptotic process](http://pantherdb.org/panther/category.do?categoryAcc=GO:0043066) | [28](http://pantherdb.org/tools/gxIdsList.do?acc=GO:0043066&reflist=1) | [6](http://pantherdb.org/tools/gxIdsList.do?acc=GO:0043066&list=gene_symbols_LE&organism=Drosophila%20melanogaster) | 0.97 | | 6.18 | | + | | 8.30E-04 | | 9.38E-02 | |
|  | [regulation of biological process](http://pantherdb.org/panther/category.do?categoryAcc=GO:0050789) | [882](http://pantherdb.org/tools/gxIdsList.do?acc=GO:0050789&reflist=1) | [50](http://pantherdb.org/tools/gxIdsList.do?acc=GO:0050789&list=gene_symbols_LE&organism=Drosophila%20melanogaster) | 30.58 | | 1.63 | | + | | 9.15E-04 | | 5.17E-02 | |
|  | [biological regulation](http://pantherdb.org/panther/category.do?categoryAcc=GO:0065007) | [1100](http://pantherdb.org/tools/gxIdsList.do?acc=GO:0065007&reflist=1) | [60](http://pantherdb.org/tools/gxIdsList.do?acc=GO:0065007&list=gene_symbols_LE&organism=Drosophila%20melanogaster) | 38.14 | | 1.57 | | + | | 8.33E-04 | | 6.28E-02 | |
|  | [cellular protein modification process](http://pantherdb.org/panther/category.do?categoryAcc=GO:0006464) | [504](http://pantherdb.org/tools/gxIdsList.do?acc=GO:0006464&reflist=1) | [5](http://pantherdb.org/tools/gxIdsList.do?acc=GO:0006464&list=gene_symbols_LE&organism=Drosophila%20melanogaster) | 17.48 | | 0.29 | | - | | 9.60E-04 | | 4.34E-02 | |
|  | [protein metabolic process](http://pantherdb.org/panther/category.do?categoryAcc=GO:0019538) | [1047](http://pantherdb.org/tools/gxIdsList.do?acc=GO:0019538&reflist=1) | [17](http://pantherdb.org/tools/gxIdsList.do?acc=GO:0019538&list=gene_symbols_LE&organism=Drosophila%20melanogaster) | 36.3 | | 0.47 | | - | | 5.03E-04 | | 1.14E-01 | |
| MF | [receptor activity](http://pantherdb.org/panther/category.do?categoryAcc=GO:0004872) | [291](http://pantherdb.org/tools/gxIdsList.do?acc=GO:0004872&reflist=1) | [24](http://pantherdb.org/tools/gxIdsList.do?acc=GO:0004872&list=gene_symbols_LE&organism=Drosophila%20melanogaster) | 10.09 | | 2.38 | | + | | 1.80E-04 | | 3.20E+00 | |
| CC | N/A |  |  |  | |  | |  | |  | |  | |
