# Supplementary material for: Hawaiian picture‐winged Drosophila exhibit adaptive population divergence along a narrow climatic gradient on Hawaii Island
Source: Ecol Evol. 2019 Feb 18;9(5):2436–48. doi: 10.1002/ece3.4844 (PMC6405895; doi:10.1002/ece3.4844)
Supplement: Supplementary file 3 [file ECE3-9-2436-s003.docx]

**Supporting Information:**

**Table S1:** Characteristic male courtship and aggression displays of *D. sproati* closely resembling *D. grimshawi* behaviors and named following Ringo and Hodosh 1978. The sex of the flies participating in each behavioral display is described in parentheses as M=male and F=female.

| **Name** | **Description** |
| --- | --- |
| Abdomen Drag *(M)* | A solitary male performs a staggered walk while flicking his wings out at 180 degrees and intermittently crouching to drag his abdomen on the ground, presumably to secret a pheromone. |
| Approach  *(M:M or M:F)* | A male walks towards another fly, male or female, with his head lowered and wings held out from his abdomen occasionally flicking them forward. Can initiate courtship or aggression displays. |
| Joust  *(M:M)* | An aggressive display where one male approaches another from the side, raises himself into a taller stance, and lashes out with kicks. It often follows an M:M approach display and typically provokes a similar response from the other male. |
| Pursuit  *(M:F)* | Following a M:F approach display and if a female does not move away or defend herself with a lunge, the approaching male will give chase to the female and attempt to circle around behind her. |
| Head-under-wing  *(M:F)* | If the pursuit is successful, the male moves behind a female, places his head under the females’ wings, and grasps her ovipositor with his mandibles. This precedes but does not necessarily lead to copulation. |
| Copulation  *(M:F)* | The male mounts the female from the head-under-wing position. The full copulation can take several minutes and males are generally inactive and may appear fully comatose for some time after, while females often continue moving during and immediately after copulation. |

**Table S2:** Intra-population characteristics of High and Low elevation populations at COII and YP1. Transitions, transversions, and amino acid changes are characterized relative to the dominant haplotype/allele.

| **Gene region** | **Elevation** | **Sequenced individuals / alleles** | **# Unique haplotypes / alleles** | **Maximum internal distance** | **Gene Diversity** | **# Mutations in intron** | **# Transitions / Transversions / Amino acid changes** |
| --- | --- | --- | --- | --- | --- | --- | --- |
| **COII** | **Low** | 34 | 6 | 3 | 0.512 +/- 0.089 | N/A | 5 / 0 / 0 |
|  | **High** | 32 | 5 | 3 | 0.587 +/- 0.090 | N/A | 4 / 0 / 0 |
| **YP1** | **Low** | 35 / 49 | 6 | 2 | 0.468 +/- 0.079 | 4 | 0 / 1 / 0 |
|  | **High** | 24 / 38 | 6 | 3 | 0.577 +/- 0.062 | 3 | 2 / 1 / 1 |

**Table S3.** Seventeen KEGG pathways identified using DAVID 6.8 (Huang et al. 2009) as over-enriched in high (HE) and low (LE) populations. Corrections for multiple tests were procured using built-in Benjamini-Hochberg FDR corrections.

| Population | [Term](https://david.ncifcrf.gov/chartReport.jsp?d-16544-s=2&d-16544-o=2&d-16544-p=1&annot=55) | Count | [%](https://david.ncifcrf.gov/chartReport.jsp?d-16544-s=6&d-16544-o=1&d-16544-p=1&annot=55) | [P-Value](https://david.ncifcrf.gov/chartReport.jsp?d-16544-s=7&d-16544-o=1&d-16544-p=1&annot=55) | FDR |
| --- | --- | --- | --- | --- | --- |
| HE | [Protein processing in endoplasmic reticulum](https://david.ncifcrf.gov/kegg.jsp?path=dme04141$Protein%20processing%20in%20endoplasmic%20reticulum&termId=550019318&source=kegg) | 28 | 3 | 3.70E-05 | 4.00E-03 |
|  | [Metabolic pathways](https://david.ncifcrf.gov/kegg.jsp?path=dme01100$Metabolic%20pathways&termId=550019285&source=kegg) | 113 | 12.3 | 4.90E-04 | 2.70E-02 |
|  | [Oxidative phosphorylation](https://david.ncifcrf.gov/kegg.jsp?path=dme00190$Oxidative%20phosphorylation&termId=550019214&source=kegg) | 27 | 2.9 | 5.30E-04 | 1.90E-02 |
|  | [Glutathione metabolism](https://david.ncifcrf.gov/kegg.jsp?path=dme00480$Glutathione%20metabolism&termId=550019238&source=kegg) | 16 | 1.7 | 5.50E-04 | 1.50E-02 |
|  | [Valine, leucine and isoleucine degradation](https://david.ncifcrf.gov/kegg.jsp?path=dme00280$Valine,%20leucine%20and%20isoleucine%20degradation&termId=550019222&source=kegg) | 9 | 1 | 7.30E-03 | 1.50E-01 |
|  | [Protein export](https://david.ncifcrf.gov/kegg.jsp?path=dme03060$Protein%20export&termId=550019303&source=kegg) | 7 | 0.8 | 1.50E-02 | 2.40E-01 |
|  | [Tryptophan metabolism](https://david.ncifcrf.gov/kegg.jsp?path=dme00380$Tryptophan%20metabolism&termId=550019230&source=kegg) | 6 | 0.7 | 3.00E-02 | 3.80E-01 |
|  | [Butanoate metabolism](https://david.ncifcrf.gov/kegg.jsp?path=dme00650$Butanoate%20metabolism&termId=550019264&source=kegg) | 5 | 0.5 | 7.30E-02 | 6.50E-01 |
|  | [Propanoate metabolism](https://david.ncifcrf.gov/kegg.jsp?path=dme00640$Propanoate%20metabolism&termId=550019263&source=kegg) | 5 | 0.5 | 7.30E-02 | 6.50E-01 |
| LE | [Dorso-ventral axis formation](https://david.ncifcrf.gov/kegg.jsp?path=dme04320$Dorso-ventral%20axis%20formation&termId=550019325&source=kegg) | 6 | 1.1 | 3.90E-03 | 2.80E-01 |
|  | [FoxO signaling pathway](https://david.ncifcrf.gov/kegg.jsp?path=dme04068$FoxO%20signaling%20pathway&termId=550019311&source=kegg) | 8 | 1.5 | 5.50E-03 | 2.10E-01 |
|  | [Tyrosine metabolism](https://david.ncifcrf.gov/kegg.jsp?path=dme00350$Tyrosine%20metabolism&termId=550019228&source=kegg) | 4 | 0.7 | 2.70E-02 | 5.40E-01 |
|  | [Synthesis and degradation of ketone bodies](https://david.ncifcrf.gov/kegg.jsp?path=dme00072$Synthesis%20and%20degradation%20of%20ketone%20bodies&termId=550019211&source=kegg) | 3 | 0.5 | 3.20E-02 | 5.00E-01 |
|  | [Metabolism of xenobiotics by cytochrome P450](https://david.ncifcrf.gov/kegg.jsp?path=dme00980$Metabolism%20of%20xenobiotics%20by%20cytochrome%20P450&termId=550019280&source=kegg) | 7 | 1.3 | 4.10E-02 | 5.10E-01 |
|  | [Drug metabolism - cytochrome P450](https://david.ncifcrf.gov/kegg.jsp?path=dme00982$Drug%20metabolism%20-%20cytochrome%20P450&termId=550019282&source=kegg) | 7 | 1.3 | 4.10E-02 | 5.10E-01 |
|  | [Ether lipid metabolism](https://david.ncifcrf.gov/kegg.jsp?path=dme00565$Ether%20lipid%20metabolism&termId=550019253&source=kegg) | 4 | 0.7 | 7.00E-02 | 6.40E-01 |
|  | [One carbon pool by folate](https://david.ncifcrf.gov/kegg.jsp?path=dme00670$One%20carbon%20pool%20by%20folate&termId=550019265&source=kegg) | 3 | 0.5 | 8.80E-02 | 6.70E-01 |

**Appendix 1.** List of *Drosophila sproati* genes in males and females significant for factor population and/or the interaction between population and temperature treatment. Gene expression was measured using an Agilent microarray designed for *D. grimshawi* (Design id Agilent-023805, GEO_20090429). The FlyBase *D. grimshawi* gene identifiers were matched to *D. melanogaster* orthologs and gene symbols for annotation purposes. Male and female flies were run on separate microarrays.

**Appendix 2.** Overenriched gene ontology (GO) categories identified from genes differentially expressed in low and high elevation *Drosophila sproati* populations. Gene expression was measured using a microarray designed for *D. grimshawi,* with GO analysis performed in PANTHER by matching *D. grimshawi* FlyBase gene identifiers to D. melanogaster (Dmel) gene symbols (see text for details). GO categories include biological process (BP), molecular function (MF), and Cellular Component (CC).
